# New Triazine Derivatives as Serotonin 5-HT_6_ Receptor Ligands

**DOI:** 10.3390/molecules28031108

**Published:** 2023-01-22

**Authors:** Dorota Łażewska, Małgorzata Więcek, Grzegorz Satała, Paulina Chałupnik, Ewa Żesławska, Ewelina Honkisz-Orzechowska, Monika Tarasek, Gniewomir Latacz, Wojciech Nitek, Ewa Szymańska, Jadwiga Handzlik

**Affiliations:** 1 Department of Technology and Biotechnology of Drugs, Faculty of Pharmacy, Jagiellonian University Medical College in Kraków, Medyczna 9, 30-688 Kraków, Poland; 2 Department of Medicinal Chemistry, Maj Institute of Pharmacology, Polish Academy of Sciences, Smętna 12, 31-343 Kraków, Poland; 3 Institute of Biology, Pedagogical University of Krakow, Podchorążych 2, 30-084 Kraków, Poland; 4 Faculty of Chemistry, Jagiellonian University in Kraków, Gronostajowa 2, 30-387 Kraków, Poland

**Keywords:** serotonin 5-HT_6_ receptor, 5-HT_6_R ligands, 1,3,5-triazine derivatives, solubility, crystal structure

## Abstract

Since the number of people with Alzheimer’s disease (AD) continues to rise, new and effective drugs are urgently needed to not only slow down the progression of the disease, but to stop or even prevent its development. Serotonin 5-HT_6_ receptor (5-HT_6_R) ligands are still a promising therapeutic target for the treatment of AD. 1,3,5-Triazine derivatives, as novel structures lacking an indole or a sulfone moiety, have proven to be potent ligands for this receptor. In present work, new derivatives of the compound MST4 (4-((2-isopropyl-5-methylphenoxy)methyl)-6-(4-methylpiperazin-1-yl)-1,3,5-triazin-2-amine), the potent 5-HT_6_R antagonist (K_i_ = 11 nM) with promising ADMET and in vivo properties, were designed. The synthesized compounds were tested for their affinity towards 5-HT_6_R and other receptor (off)targets (serotonin 5-HT_2A_, 5-HT_7_ and dopamine D_2_). Based on the new results, 4-(2-tert-butylphenoxy)-6-(4-methylpiperazin-1-yl)-1,3,5-triazin-2-amine (**3**) was selected for extended in vitro studies as a potent and selective 5-HT_6_R ligand (K_i_ = 13 nM). Its ability to permeate the blood–brain barrier (BBB) and its hepatotoxicity were evaluated. In addition, X-ray crystallography and solubility studies were also performed. The results obtained confirm that 6-(4-methylpiperazin-1-yl)-1,3,5-triazin-2-amine derivatives, especially compound **3**, are promising structures for further pharmacological studies as 5-HT_6_R ligands.

## 1. Introduction

**Figure 1:**
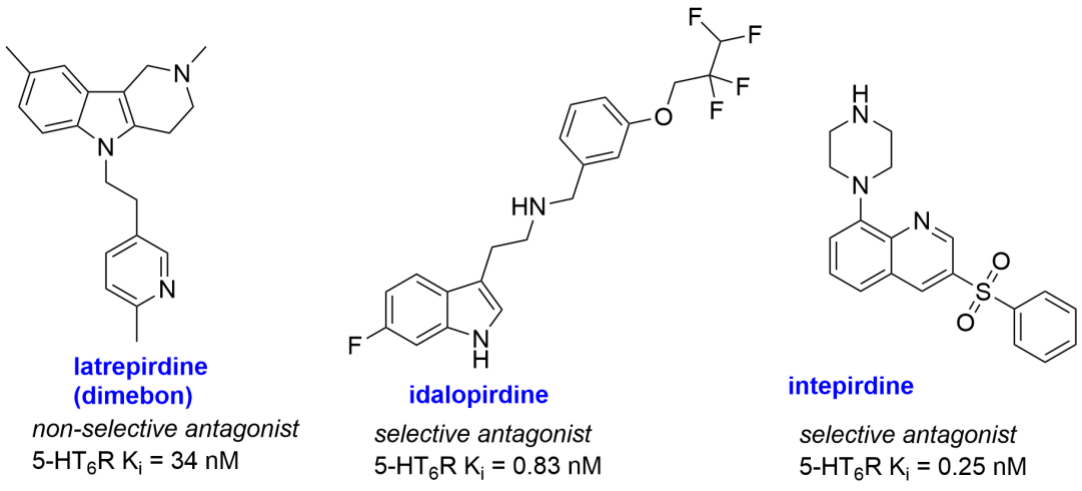
Structure and 5-HT_6_ receptor affinity of compounds that reached phase III. Data from [8].

Alzheimer’s disease (AD) is a progressive, neurodegenerative disorder with a complex etiology. It is currently estimated that the number of people with the disease in Europe will double by 2050 compared to today [1]. The largest increase is expected in poor and middle-income countries. The average time from the onset of the disease, which is not noticeable, through the onset of cognitive impairment to dementia is between 15 and 25 years. AD most often affects people over 65 years of age and depends on genetic predisposition (60–80%). Moreover, women are more likely to develop this disease than men. The search for effective drugs for AD is not easy. There are currently three acetylcholinesterase inhibitors (donepezil, rivastigmine and galantamine) and one NMDA antagonist (memantine) in therapeutic use. Memantine was, for 18 years (from 2003), the last new chemical drug registered for the treatment of this disease [2]. Quite recently, in 2021, the FDA approved, through a fast-track approval pathway and despite doubts about its efficacy, a new biological drug to fight AD—aducanumab (Aduhelm^®^), which is a monoclonal antibody [3]. It is the first drug to slow the progression of the disease by acting on beta-amyloid deposits. The deposition of beta-amyloid plaques is one of the characteristic features of AD. The plaques play a role in damaging nerve cells and causing other undesirable changes in the brain. Aducanumab is registered for patients with an early diagnosis of AD (mild cognitive impairment and mild dementia) [4]. In addition to the search for new drugs, other forms of administration are being sought to facilitate their use while limiting gastrointestinal side effects, such as donepezil transdermal patches (Adlarity^®^) delivering 5 or 10 mg/day of the drug for seven days, approved in March of this year by the FDA (14 March 2022) [5]. Although the variety of drugs on the market for the treatment of AD is small, the number of compounds reaching clinical trials is still high. More recently, Cumming et al. conducted an analysis of clinical trials registered on the website https://clinicaltrials.gov that were ongoing in January of this year (as of 25 January 2022) in the United States, where the target was AD therapy [6]. In the investigated time, 143 agents were involved in 172 clinical trials belonging to four groups: memory enhancers, biological disease modifiers, small-molecule disease-modifying compounds and compounds treating neuropsychiatric and behavioral symptoms. Most of the tested compounds were in phase II clinical trials and were classified as disease-modifying substances (83.2%). This ongoing work creates opportunities to bring new therapeutic agents to the market in the future. Currently, the rational design of such compounds based on molecular modelling plays an important role in obtaining new therapeutic products. Furthermore, the use of disease biomarkers facilitates diagnosis and is helpful in assessing the course of the disease. Interestingly, serotonin 5-HT_6_ receptor (5-HT_6_R) antagonists, i.e., idalopirdine, intepirdine or latrepirdine (Figure 1), were an interesting group of compounds investigated for potential use in the treatment of AD. These compounds reached phase III of clinical trials. However, the studies did not show a positive effect of the tested compounds on cognition when used as adjuncts to cholinesterase inhibitors [7]. Although these trials have not been successful, 5-HT_6_R, with its many promising preclinical studies, continues to be a hopeful therapeutic target for the treatment of neurodegenerative diseases. Much research is being performed now to find new potent ligands and to study the role of this receptor in the development and progression of diseases [7,8,9].

**Figure 2:**
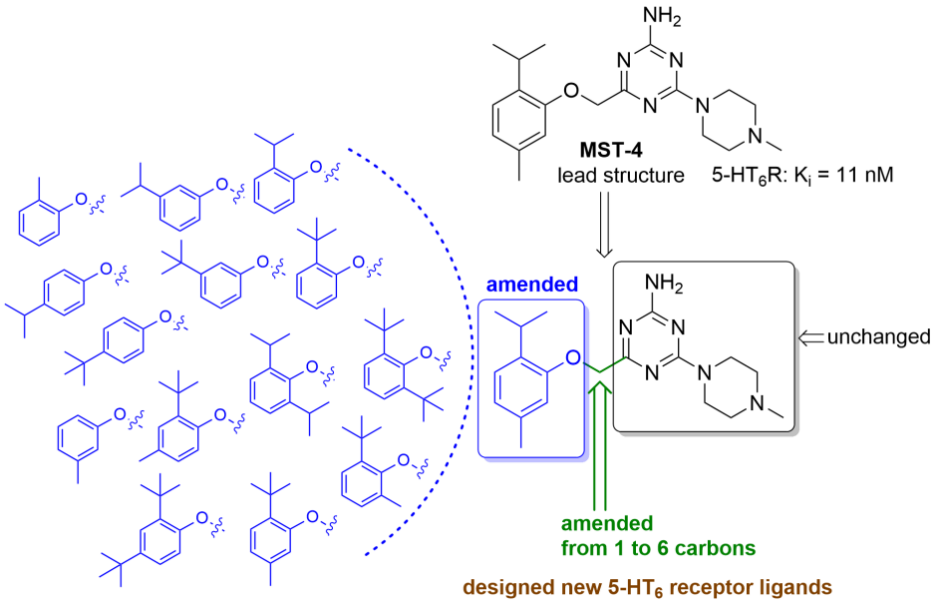
Design of novel compounds based on promising lead compound **MST4**.

5-HT_6_R is found almost exclusively in the central nervous system, where it particularly moderates GABA and glutamate levels and facilitates the release of neurotransmitters such as dopamine, norepinephrine and acetylcholine, all of which are compromised in AD. A recent in vitro (postmortem) study using the PET radiotracer [^18^F]2FNQ1P showed a decrease in 5-HT_6_R density in AD patients in the caudate nucleus, a region normally rich in this receptor [10]. Furthermore, a systematic analysis of animal and human studies by Corey and Quednow on the role of serotonin in declarative memory showed that there is a marked decrease in serotonin in the brain and that 5-HT_1A_R antagonists, 5-HT_4_R agonists and 5-HT_6_R antagonists may be helpful in improving this memory [11]. Over the past several years, our research group has been involved in the search for new 5-HT_6_R ligands among 1,3,5-triazine derivatives [12,13,14,15,16,17,18]. Such derivatives represent an original chemical group, lacking sulfonic and/or indole moieties, which are characteristic elements of the majority of 5-HT_6_R ligands [8]. As a result of our previous work, we have described many promising compounds; among them was **MST4**, a compound with a high affinity for 5-HT_6_R, promising ADMET (absorption, distribution, metabolism, excretion, and toxicity) properties and in vivo pro-cognitive activity (Figure 2) [16]. In the present study, this compound was chosen as the lead structure, and the modifications were designed as shown in Figure 2. The synthesized compounds were evaluated in vitro for receptor affinity, cell membrane permeability, and hepatotoxicity. In addition, the most promising molecule was transformed into different salts to check its 5-HT_6_R affinity, solubility and crystallographic structure.

## 2. Results and Discussion

### 2.1. Synthesis of Compounds

**Scheme 1:**
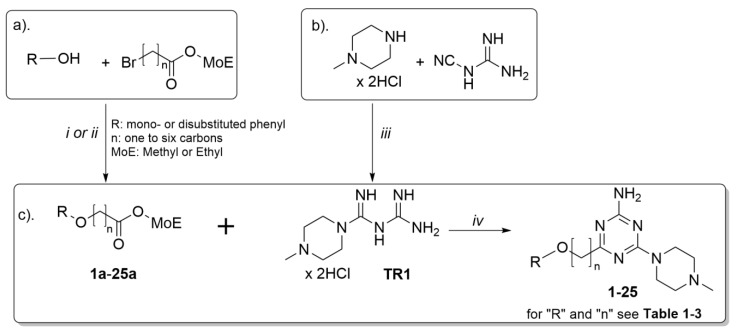
Synthetic way of designed compounds **1**–**25**. (**a**) Synthesis of esters **1a**-**25a**; (**b**) Synthesis of **TR1**; (**c**) Synthesis of final triazines **1**-**25**. Reagents and conditions: (i) K_2_CO_3_, acetone or acetonitrile, reflux 12–24h; (ii) Cs_2_CO_3_, acetone, reflux 12–24 h; (iii) butanol, temperature gradually increased from 50 to 90 °C during 1 h, 12–24 h reflux; (iv) CH_3_ONa (freshly prepared), room temp 12–48 h or reflux 15–30 h.

Esters (reaction a; Scheme 1) were prepared and purified as previously described by Ali et al. [15]. The exact synthetic process and physicochemical data for the selected esters are presented in the Supplementary Material S1. Unfortunately, under these reaction conditions, we could not obtain esters via *O*-alkylation of various substituted phenols (2-isopropyl, 2-isopropyl-5-methyl and 3-methyl) with methyl 3-bromopropionate. TR1 was obtained through the condensation of piperazine dihydrochloride with cyanoguanidine (reaction b; Scheme 1) [19]. Next, the obtained esters were cyclized with TR-1 (reaction c; Scheme 1). Equimolar amounts of reagents were taken for this reaction, and it was carried out in an alkaline medium of sodium methanolate. The course of the reaction was controlled via thin layer chromatography. The final products were obtained as free bases or carried out as hydrochlorides. The purity and identity of the compounds were confirmed using spectral analysis (^1^H NMR, ^13^C NMR, and LC/MS) (Supplementary Material S2).

### 2.2. Pharmacology

#### 2.2.1. In Vitro Affinity for 5-HT_6_ Receptor

The affinity of compounds (**1**–**25**) for 5-HT_6_R was evaluated in the radioligand binding assay in HEK293 cells stably expressing human 5-HT_6_R. [^3^H]-LSD as a radioligand was used [12]. All compounds showed affinity for 5-HT_6_R, but the strength of the interaction with this receptor depended on the type of substituent in the phenyl ring, the type of substitution (mono- or di-) and the length of the linker between the triazine ring and the phenyl substituent. All results are collected in Table 1, Table 2 and Table 3.

First (series 1), modifications were carried out in the **MST4** molecule involving the removal of an isopropyl group from position-2 (compound **1**) and, conversely, the removal of the methyl group from position-5 (compound **2**). These modifications showed that the substituent at position-2 was important, as removal of a 2-isopropyl substituent resulted in a 19-fold decrease in affinity for 5-HT_6_R compared to **MST4** (K_i_ = 207 nM for **1** vs. K_i_ = 11 nM for **MST4**). In contrast, removal of the methyl group from position-5 (compound **2**) resulted in only a 2-fold decrease in activity (K_i_ = 21 nM). On the other hand, replacing the 2-isopropyl substituent with the 2-*tert*-butyl substituent led to compound **3**, which had comparable activity to **MST4** (K_i_ = 13 nM for **3** vs. K_i_ = 11 nM for **MST4**). Furthermore, changing the position in the phenyl ring of both the isopropyl and the *tert*-butyl substituents from position-2 to positions-3 or -4 generally resulted in a decrease in activity (compare **2** vs. **4** vs. **6** and **3** vs. **5** vs. **7**). Compounds **6** and **7** with substituents in position-4 had the lowest affinity (K_i_ = 416 nM and K_i_ =150 nM, respectively) in this series.

In series 2, disubstituted compounds such as **MST4** (**8**–**13**; Table 2) were obtained, which had one or two *tert*-butyl substituents in the phenyl ring (except compound **11** with 2,6-di-isopropyl groups). Compound **11** was the least active in this series (K_i_ = 825 nM), and its affinity at 5-HT_6_R was almost two times weaker than an analogue compound with 2,6-di-*tert*-butyl substituents (compound **12**: K_i_ = 379 nM). Among compounds **8**–**10**, where the methyl substituent at position-5 (a direct analogue of **MST4**) was shifted to another position (4 or 6), the affinity for 5-HT_6_R decreased in the order of the position of the methyl substituent: 5 (compound **8**) > 4 (compound **9**) > 6 (compound **10**) with K_i_ values of 6 nM vs. 48 nM vs. 236 nM, respectively. Compound **8**, the direct analogue of **MST4**, proved to be the most potent 5-HT_6_R agent in that series as well as among all designed compounds.

The introduction of the second substituent in position-6 had negative influence on 5-HT_6_R affinity, and the increased volume of this substituent (from a methyl to a *tert*-butyl) decreased affinity; compare **10** (6-methyl; K_i_ = 236 nM) vs. **12** (6-*tert-*butyl; K_i_ = 379 nM).

In series 3, the influence on elongation of the carbon chain (from two to six) was investigated (Table 3). A variable impact of this effect on activity was observed. An even number of carbon atoms seems to be more favorable than an odd number. The hexyl derivative (compound **25**), with a K_i_ of 78 nM, had the highest affinity in this series. In the group of 2-isopropyl-5-methylphenyl derivatives (compounds **14**, **19**, **22** and **25**), the affinity depending on the number of carbon atoms in the linker is arranged as follows: 6 > 4 > 5 > 3 atoms. Similarly, in the group of 2-isopropylphenyl (compounds **15**, **20** and **23**) and 3-methylphenyl (compounds **17**, **21** and **24**) derivatives, the affinity increase was observed in the order 4 > 5 > 3 linkers.

#### 2.2.2. In Vitro Affinity at Other Tested Receptors

Affinity for serotonin 5-HT_2A_, 5-HT_7_ and dopamine D_2_ receptors (D_2_R) as protein off-targets was evaluated in the radioligand binding assays. In CHO-K1 cells, it was stably expressed human 5-HT_2A_R, whereas human 5-HT_7_R and D_2_R were stably expressed in HEK293 cells [12]. Most compounds showed a much weaker affinity for other receptors than for 5-HT_6_R, especially for 5-HT_7_R. A particularly weak affinity towards this receptor was shown through compounds of series 1 (compounds **1**–**7**) and series 2 (compounds **8**–**13**). Among series 3 (compounds **14**–**25**), there were compounds that showed submicromolar affinity for this receptor (i.e., compound **19**: K_i_ = 638 nM; compound **20**: K_i_ = 665 nM; and compound **22**: K_i_ = 771 nM). With regards to the affinity for 5-HT_2A_R, among the tested compounds, there were some that showed good affinity for this receptor with K_i_ < 1000 nM. The largest number of such derivatives was in series 3 (i.e., compounds **15**, **19**–**25**). There were also compounds with a good affinity for this receptor with K_i_ < 500 nM, such as compound **6** (K_i_ = 248 nM), compound **15** (K_i_ = 489 nM) and compound **25** (K_i_ = 364 nM).

Concerning the affinity for D_2_R, compounds of series 3 also showed the highest affinity (compounds: **16**, **21**–**25**), including compound **23,** where the potency of the interaction with 5-HT_6_R was comparable to that with D_2_R: K_i_ = 196 nM vs. 5-HT_6_R: K_i_ = 189 nM. To sum up, carbon-chain elongation, although having a variable effect on affinity for 5-HT_6_R, definitively causes a decrease in selectivity.

### 2.3. Additional Studies for Compound ***3***

#### 2.3.1. Permeability of Compound **3**

The PAMPA assay was used to test the ability of compound **3** to cross the blood–brain barrier (BBB). This test is a very popular method for assessing BBB penetration via passive transport. Caffeine was used as the high-permeable compound, and results from previous studies [16] for **MST4** were added for comparison. All results are summarized in Table 4, and they show that compound **3** has the ability to passively penetrate through biological membranes with P_e_ of 4.7 × 10^−6^ cm/s. Although this value is lower than for caffeine (CFN) (study a: P_e_ = 9.8 × 10^−6^ cm/s), it is still high. An earlier study for **MST4** (study b) showed that this compound demonstrated high permeability with P_e_ of 12.3 × 10^−6^ cm/s. The penetration value in that study (study b) for CFN was higher than in the current study (study a). The apparent difference in these values makes it impossible to directly compare the permeation capacity of compound **3** with that of **MST4**. However, it appears to be only a little smaller, which indicates that the modifications that were introduced into the structure did not significantly influence the permeation capacity.

#### 2.3.2. Hepatotoxicity of Compound **3**

**Figure 3:**
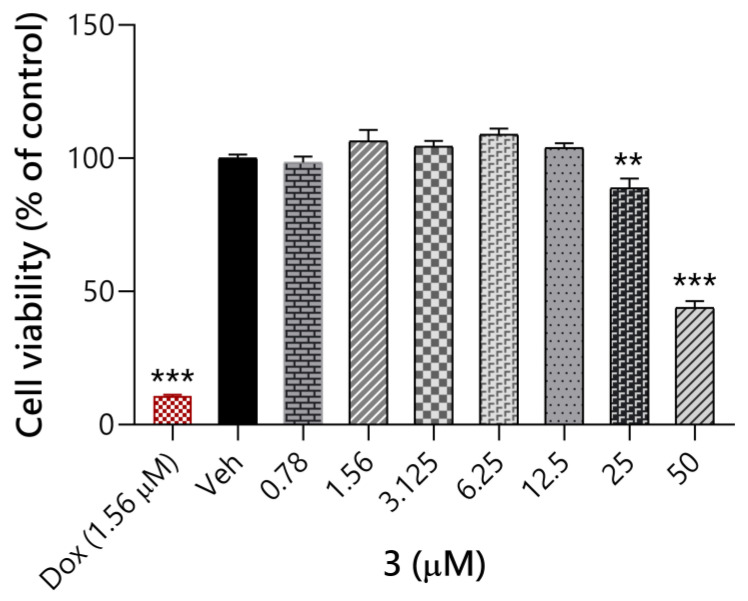
The effect of compound **3** on HepG2 cells’ viability. The graphs were generated using GraphPad Prism software for IC_50_ calculations based on MTS data. Each point represents the mean ± SEM of two independent experiments, each of which consisted of three replicates per treatment group and is expressed as a percentage of control cells treated with 0.1% of DMSO (Veh). Doxorubicin (DOX) serves as a common chemotherapeutic agent, which induced hepatotoxicity. All statistical analyses were performed using GraphPad Prism software 8.0. Statistical significance was evaluated via one-way ANOVA with a post-hoc Dunnett test at significance level α = 0.05 (** *p* = 0.003, *** *p* < 0.001).

Some substances can lead to liver damage. To eliminate this risk, hepatotoxicity tests are carried out as early as the pre-clinical development phase. One of the methods commonly used to assess the adverse effect of tested compounds on cell viability is the MTT assay, and the most popular cell lines are HepG2 and HepaRG (the human hepatocellular carcinoma cells) [20]. To evaluate the toxic effect of compound **3**, the MTS assay (one-step MTT assay variant) was performed on the HepG2 cells. HepG2 cells were incubated with increasing concentrations (0.78–50 µM) of compound **3** for 48 h. After that time, the MTS reagent was added and incubated for 1 h. Next the absorbance was read at 490 nm. A dose–response effect is shown in Figure 3. The calculated IC_50_ value was 46.60 µM, which gave us reason to conclude that compound **3** exhibited moderate hepatotoxicity and was appropriate for further development.

### 2.4. Salts of Compound ***3***

#### 2.4.1. Pharmacological Evaluation

The salt form in which a drug is present also influences its physicochemical and biological properties. The right salt form can either have a beneficial effect on the pharmacological action or, on the contrary, be detrimental [21].

Compound **3** has been converted into two salts, hydrochloride (**3-HCl**) and succinate (**3-SA**). The resulting compounds were tested for affinity to 5-HT_6_R and 5-HT_7_R. The data are presented in Table 5. The results showed that the type of salt influenced pharmacological activity. Both salts had a lower affinity for 5-HT_6_R than the free base, although in the case of hydrochloride (**3-HCl**), the decrease was only 3-fold (K_i_ = 35 nM vs. K_i_ = 13 nM for **3**), and in the case of succinate (**3-SA**), as much as 10-fold (K_i_ = 135 nM). In contrast, the affinity for 5-HT_7_R was different. It was still weak in the micromole range, but in the case of succinate (**3-SA**), an increase in activity was observed (K_i_ = 3.67 µM vs. K_i_ = 6.34 µM for 3). Hydrochloride (**3-HCl**), on the other hand, showed an even greater decrease in activity at 5-HT_7_R (K_i_ = 15.50 µM) than compound **3**.

#### 2.4.2. Salts of Compound **3**—Solubility Evaluation

The aqua solubility of compound **3** and its salts (**3-HCl** and **3-SA**) was assessed experimentally using the UV spectroscopic method described previously [22,23] (See Supplementary S1, pp. 16–17). Results are shown in Table 6. Under neutral pH conditions (pH = 7 ± 1), the piperazine moiety undergoes protonation and exists as a mixture of cation and free base, which can affect the solubility of the tested derivatives. The experimental results clearly indicate that the conversion of compound **3** into its salt form results in an improvement in solubility. In the case of the hydrochloric acid salt (**3-HCl**), the increase is quite significant (>1 mg/mL) compared to the parent compound. Meanwhile, the determined solubility value of the succinic acid salt (**3-SA**) was noticeably higher compared to compound **3**, but still relatively low (<1 mg/mL). Furthermore, when analyzing the in vitro pharmacological results, no clear correlation between the affinity and the determined solubility of the tested compounds was found. However, these preliminary results showed that, if the final product had to be carried into the salt, the formation of hydrochlorides could be beneficial, as increasing solubility had only a slight effect on affinity.

#### 2.4.3. Crystallographic Studies of Compound **3** and Its Salts

Attempts were made to crystallize compound **3** and its salts to obtain suitable crystals for X-ray analysis. Crystals were successfully obtained for compound **3** and its succinic acid salt (**3-SA**). Unfortunately, for the hydrochloride of compound **3** (**3-HCl**), the attempts were unsuccessful.

**Figure 4:**
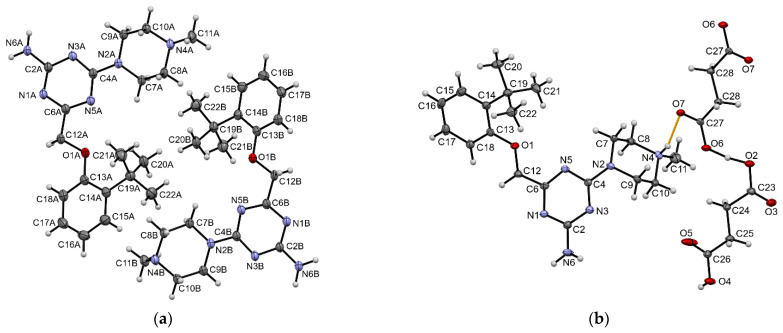
The contents of the asymmetric units of (**a**) compound **3** and (**b**) compound **3-SA** (the whole anion is drawn), showing the atom-numbering schemes. Displacement ellipsoids are drawn at the 50% probability level. The charge-assisted hydrogen bond in compound **3-SA** is depicted in orange.

The projections of molecular geometry in the crystals of compound **3** and its succinic acid salt (**3-SA**) with atom-numbering schemes are presented in Figure 4. Compound **3** crystallizes with two molecules in the asymmetric unit (labeled A and B). The salt **3-SA** crystallizes with one cation derived from protonation of the N4 atom of compound **3**, one molecule of succinic acid and half of the succinate anion in the asymmetric unit.

The triazine ring is planar, with an r.m.s. deviation from the planarity of the fitted atoms of 0.0031 Å and 0.004 Å for molecules A and B of compound **3**, respectively. In the structure of salt 3-SA, this ring is less planar; an r.m.s. deviation from the planarity of the fitted atoms is 0.0189. The values of the bond lengths of C2-N6 and C4-N2 suggest conjugation of nitrogen atoms with the triazine ring (Table 7). The piperazine ring adopts chair conformation with an equatorial position of the methyl group for molecule A of compound **3** and the cation of compound **3-SA**, while for molecule B of compound **3,** the axial position is observed. This is the first time we have noticed such an arrangement of the *N*-methylpiperazine moiety in the so-far determined crystal structures containing (4’-methylpiperazin-1′-yl)-1,3,5-triazine moiety [17,24]. Therefore, we searched the Cambridge Structural Database (CSD, Version 5.43; [25]) for crystal structures containing the *N*-methylpiperazine moiety. The search resulted in 311 hits, with all structures containing the methyl group in the equatorial position. The presented crystal structure of compound **3** is the first structure containing in the axial position a methyl group at nitrogen atom of the piperazine ring.

**Figure 5:**
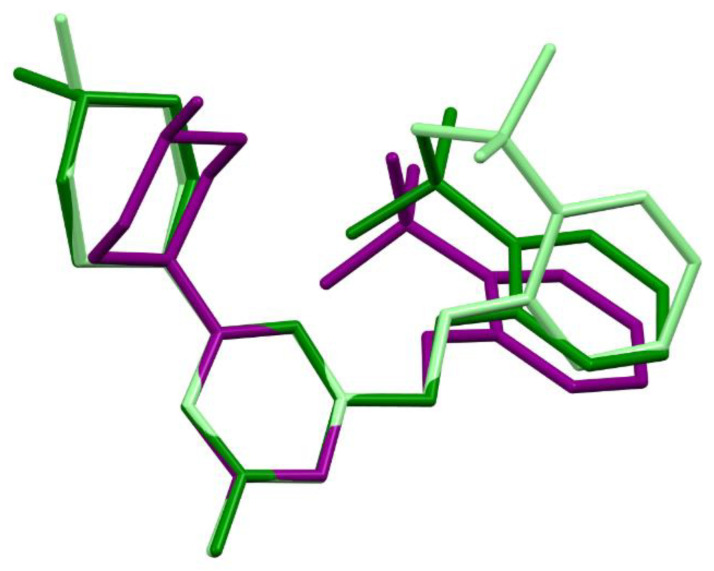
The overlap of the triazine rings of molecule A (light green) and molecule B (green) of compound **3** and the cation of salt **3-SA** (purple). H atoms have been omitted for clarity.

The 4-(piperazin-1′-yl)-1,3,5-triazine moiety shows similar geometry in two molecules of compound **3**, while in compound **3-SA** it is different. The interplanar angle between the triazine and piperazine rings is 36.5(1)° (molecule A of **3**), 39.5(1)° (molecule B of **3**) and 81.92(4)° (**3-SA**) (Figure 5). More diverse geometries are observed in 2-*tert*-butylphenoxy fragment. Thus, these differences are best illustrated by the values of torsion angles C6-C12-O1-C13 and C12-O1-C13-C18 (Table 7). The oxygen atom stabilizes the geometry of molecules in both structures using intramolecular hydrogen bonds C-H···O with two methyl groups of the 2-*tert*-butyl substituent.

The intermolecular interactions of the crystals of compound **3** are dominated by N-H···N and C-H···N intermolecular hydrogen bonds. A greater diversity of intermolecular interactions is observed in the crystals of salt **3-SA**. Due to the protonation of the N4 atom by proton transfer from molecules of succinic acid, this atom is involved in the charge-assisted N+-H···O¯ hydrogen bond (Figure 4). Furthermore, the N-H···O, O-H···N, O-H···O and C-H···O intermolecular hydrogen bonds are observed.

## 3. Materials and Methods

### 3.1. Chemistry

Reagents and solvents were purchased from commercial suppliers (Sigma–Aldrich, Alfa Aesar). Reactions were carried out in an air atmosphere and monitored using thin-layer chromatography (Merck silica gel 60 F254 plates). Visualization of the spots was achieved using a UV lamp and Dragendorff reagent (solvent system: methylene chloride or methylene chloride: methanol 1:1). Melting points (mp) were determined using MEL-TEMP II apparatus (LD Inc., Long Beach, CA, USA) or a Büchi M-565 apparatus (Büchi Labortechnik AG, Switzerland), and are uncorrected. The purity of the compounds was confirmed using an NMR spectra (^1^H and ^13^C) in DMSO-d_6_ using a Mercury 300 MHz PFG spectrometer (Varian, Palo Alto, CA, USA) or FTNMR 500 MHz spectrometer (Joel Ltd., Akishima, Tokyo, Japan). Chemical shifts (δ) are given with respect to the solvent signal, and the coupling constants (*J*) are expressed in Hz. Multiplicities of signals are given as br s (broad singlet), d (dublet), dd (doublet of doublets), def t (deformated triplet), m (multiplet), quin (quintet), s (singlet), spt (septet), and t (triplet). The following abbreviations are used to report data: a (axial), e (equatorial), def (deformed), Pp (piperazine), Tr (triazine) (as shown in the Supplementary Material S2). Mass spectra (LC/MS) were performed using a Waters TQ Detector mass spectrometer (Water Corporation, Milford, CT, USA). Retention times (t_R_) are given in minutes. UPLC/MS analysis confirmed the purity of the compounds ≥ 95%.

#### 3.1.1. Synthesis of Esters

Esters were obtained according to the method described previously [19]. More information can be found in the Supplementary Material S1.

#### 3.1.2. Synthesis of 1,3,5-Triazines

1,3,5-Triazines were obtained according to the method described previously [19].

General procedure: To a freshly prepared sodium methanolate solution (10 mL) was added TR1 (5 mmol), which was stirred at room temperature for 1 to 3 h, and then the appropriate ester (5 mmol) was added in one portion and stirred at room temperature for 12 to 48 h (**3**, **5**, **7**–**10**, **12**, **13**, **17**, **22**–**25**) or heated to boiling for 15 to 30 h (**2**, **4**, **6**, **11**, **14**-**16**, **18**–**21**). The solvent was evaporated, and 10 mL of water was added to the residue and stirred at room temperature for 24 h. The precipitate was isolated by filtration and purified via crystallization. In the case of a lack of desirable precipitate in the water solution, the product was extracted using dichloromethane and converted into a hydrochloric salt form using a solution of HCl in diethyl ether.

##### 4-((2-Isopropylphenoxy)methyl)-6-(4-methylpiperazin-1-yl)-1,3,5-triazin-2-amine (**2**)

Crystallization: ethanol, white solid, yield 29%, mp 140–142 °C. C_18_H_26_N_6_O (MW 342.45). LC/MS^+^: purity: 100%, t_R_ = 4.18, (ESI) *m/z* [M+H]^+^ 343.25. ^1^H NMR (300 MHz, DMSO-d_6_) δ: 7.16 (d, *J* = 7.03 Hz, 1H, Ph-3-*H*), 7.06 (t, *J* = 7.62 Hz, 1H, Ph-4-*H*), 6.85 (m, 3H, Ph-2,6-*H*, N*H*_2_), 4.78 (s, 2H, OC*H_2_*), 3.64 (br s, 4H, Pp-3,5-*H*), 3.32 (m, 2H, C*H*(CH_3_)_2_ + *H*_2_O), 2.23 (br s, 4H, Pp-2,6-*H*), 2.15 (s, 3H, Pp-C*H*_3_), 1.17 (d, *J* = 6.45 Hz, 6H, CH(C*H_3_*)*_2_*). ^13^C NMR (75 MHz, DMSO-d_6_) δ: 173.7, 167.3, 164.9, 156.1, 136.7, 127.0, 126.3, 121.1, 112.4, 70.2, 54.8, 46.3, 27.0, 23.0.

##### 4-((2-(*tert*-Butyl)phenoxy)metyl)-6-(4-methylpiperazin-1-yl)-1,3,5-triazin-2-amine (**3**)

Crystallization: acetonitrile, white solid, yield 42%, mp 107–109 °C. C_19_H_28_N_6_O (MW 356.47). LC/MS^+^: purity: 100%, t_R_ = 4.57, (ESI) *m/z* [M+H]^+^ 357.28.^1^H NMR (300 MHz, DMSO-d_6_) δ: 7.20 (d, *J* = 7.62 Hz, 1H, Ph-3-*H*), 7.12 (def t, 1H, Ph-5-*H*), 6.77–7.00 (m, 4H, N*H_2_* + Ph-4,6-*H*), 4.77 (s, 2H, OC*H_2_*), 3.68 (br s, 4H, Pp-3,5-*H*), 2.25 (br s, 4H, Pp-2,6-*H*), 2.15 (s, 3H, Pp-C*H_3_*), 1.37 (s, 9H, C(C*H_3_*)*_3_*). ^13^C NMR (126 MHz, DMSO-d_6_) δ: 173.5, 167.2, 165.0, 157.5, 137.8, 127.6, 126.7, 120.8, 112.9, 69.9, 54.8, 46.3, 43.1, 35.1, 30.2, 30.1.

##### 4-((3-Isopropylphenoxy)methyl)-6-(4-methylpiperazin-1-yl)-1,3,5-triazin-2-amine (**4**)

White solid, yield 44%, mp 103–105 °C. C_18_H_26_N_6_O (MW 342.45). LC/MS^+^: purity: 100%, t_R_ = 5.08, (ESI) *m/z* [M+H]^+^ 343.20. ^1^H NMR (500 MHz, DMSO-d_6_) δ: 7.12 (t, *J* = 7.88 Hz, 1H, Ph-5-*H*), 6.80–7.04 (m, 2H, N*H_2_*) 6.73–6.79 (m, 2H, Ph-4,6-*H*), 6.67 (dd, *J* = 8.02, 2.00 Hz, 1H, Ph-2-*H*), 4.71 (s, 2H, OC*H_2_*), 3.62 (d, *J* = 4.30 Hz, 4H, Pp-3,5-*H*), 2.78 (spt, *J* = 6.87 Hz, 1H, C*H*(CH_3_)_2_), 2.23 (br s, 4H, Pp-2,6-*H*), 2.14 (s, 3H, Pp-C*H_3_*), 1.12 (d, *J* = 6.87 Hz, 6H, CH(C*H_3_*)_2_ ). ^13^C NMR (126 MHz, DMSO-d_6_) δ: 168.1, 162.2, 158.0, 150.7, 129.8, 120.2, 114.0, 112.6, 67.3, 51.7, 42.4, 34.0, 24.3.

##### 4-((3-(*tert*-Butyl)phenoxy)methyl)-6-(4-methylpiperazin-1-yl)-1,3,5-triazin-2-amine (**5**)

Crystallization: ethanol/water, white solid, yield 16%, mp 101.2–104.1 °C. C_19_H_28_N_6_O (MW 356.47). LC/MS^+^: purity: 100%, t_R_ = 4.44, (ESI) *m/z* [M+H]^+^ 357.28. ^1^H NMR (300 MHz, DMSO-d_6_) δ: 7.08–7.22 (m, 1H, Ph-5-*H*), 6.77–7.08 (m, 4H, N*H_2_* + Ph-2,4-*H*), 6.64–6.77 (m, 1H, Ph-6-*H*), 4.74 (s, 2H, OC*H_2_*), 3.65 (br s, 4H, Pp-3,5-*H*), 2.25 (br s, 4H, Pp-2,6-*H*), 2.16 (s, 3H, Pp-C*H_3_*), 1.22 (s, 9H, C(C*H_3_)_3_*). ^13^C NMR (126 MHz, DMSO-d_6_) δ: 173.7, 167.4, 164.8, 158.8, 152.7, 129.4, 118.1, 112.8, 111.8, 70.3, 54.8, 46.3, 42.9, 34.9, 31.6.

##### 4-((4-Isopropylphenoxy)methyl)-6-(4-methylpiperazin-1-yl)-1,3,5-triazin-2-amine (**6**)

Crystallization: methanol, white solid, yield 26%, mp 133–135 °C. C_18_H_26_N_6_O (MW 342.45). LC/MS^+^: purity: 100%, t_R_ = 4.06, (ESI) *m/z* [M+H]^+^ 343.19.^1^H NMR (300 MHz, DMSO-d_6_) δ: 7.10 (d, *J* = 8.20 Hz, 2H, Ph-3,5-*H*), 7.00 (br s, 2H, N*H_2_*), 6.80 (d, *J* = 8.79 Hz, 2H, Ph-2,6-*H*), 4.71 (s, 2H, OC*H_2_*), 3.64 (br s, 4H, Pp-3,5-*H*), 2.79 (spt, *J* = 7.04 Hz, 1H, C*H*(CH_3_)_2_), 2.25 (br s, 4H, Pp-2,6-*H*), 2.16 (s, 3H, Pp-C*H_3_*), 1.13 (d, *J* = 7.03 Hz, 6H, CH(C*H_3_*)*_2_*). ^13^C NMR (126 MHz, DMSO-d_6_) δ: 173.6, 167.3, 164.8, 157.1, 141.0, 127.5, 114.9, 70.2, 54.8, 46.3, 42.9, 33.1, 24.6.

##### 4-((4-(*tert*-Butyl)phenoxy)methyl)-6-(4-methylpiperazin-1-yl)-1,3,5-triazin-2-amine (**7**)

Crystallization: methanol/water, white solid, yield 35%, mp 104.4 °C dec. C_19_H_28_N_6_O (MW 356.47). LC/MS^+^: purity: 100%, t_R_ = 4.39, (ESI) *m*/*z* [M+H]^+^ 357.28. ^1^H NMR (300 MHz, DMSO-d_6_) δ: 7.24 (d, *J* = 8.79 Hz, 2H, 2,6-H), 7.14-6.71 (m, 4H, Ph-3,5-H + NH_2_), 4.72 (s, 2H, OCH_2_), 3.64 (br s, 4H, Pp-3,5-H), 2.07–2.32 (m, 7H, Pp-2,6-H + Pp-CH_3_), 1.22 (s, 9H, C(CH_3_)_3_). ^13^C NMR (126 MHz, DMSO-d_6_) δ: 173.6, 167.3, 164.8, 156.7, 143.2, 126.5, 114.6, 70.1, 54.8, 46.3, 42.8, 34.3, 31.9.

##### 4-((2-(*tert*-Butyl)-5-methylphenoxy)methyl)-6-(4-methylpiperazin-1-yl)-1,3,5-triazin-2-amine hydrochloride (**8**)

Crystallization: methanol/diethyl ether, white solid, yield 19%, mp 259 °C dec. C_20_H_30_N_6_O x HCl (MW 406.99). LC/MS^+^: purity: 100%, t_R_ = 5.10, (ESI) *m/z* [M+H]^+^ 371.30. ^1^H NMR (500 MHz, DMSO-d_6_) δ: 11.58 (br s, 1H, N*H*^+^), 7.26–7.89 (m, 2H, N*H_2_*), 7.06 (d, *J* = 8.02 Hz, H, Ph-*3*-*H*), 6.76 (br s, 1H, Ph-*6*-*H*), 6.66 (d, *J* = 7.45 Hz, 1H, Ph-*4*-*H*), 4.88 (br s, 2H, Ph-O-C*H_2_*−), 4.30–4.75 (m, 4H, Pp-*3,5*-*H_2_*), 3.43 (br s, 2H, Pp-*2,6*-*H_2e_*), 2.98 (br s, 2H, Pp-*2,6*-*H_2a_*), 2.69 (s, 3H, PhC*H_3_*), 2.20 (s, 3H, Pp-C*H_3_*), 1.33 (s, 9H, C(C*H_3_)_3_*). ^13^C NMR (126 MHz, DMSO-d_6_) δ: 163.5, 156.9, 136.9, 135.0, 126.7, 121.9, 114.1, 68.2, 51.9, 42.4, 34.8, 30.3, 21.2.

##### 4-((2-(*tert*-Butyl)-4-methylphenoxy)methyl)-6-(4-methylpiperazin-1-yl)-1,3,5-triazin-2-amine (**9**)

Crystallization: methanol/water, white solid, yield 56%, mp 146.5 °C dec. C_20_H_30_N_6_O (MW 370.49). LC/MS^+^: purity: 100%, t_R_ = 5.01, (ESI) *m/z* [M+H]^+^ 371.30. ^1^H NMR (400 MHz, DMSO-d_6_) δ: 7.02 (s, 1H, Ph-3-*H*), 6.92 (d, *J* = 8.61 Hz, 1H, Ph-*5*-H), 6.87 (br s, 2H, N*H_2_*), 6.81 (d, *J* = 8.22 Hz, 1H, Ph-*6*-H), 4.74 (s, 2H, C*H_2_*O), 3.71 (br s, 4H, Pp-3,5-*H*), 2.09–2.36 (m, 10H, Pp-2,6-*H* + Pp-C*H_3_* + Ph-C*H_3_*), 1.38 (s, 9H, C(C*H_3_)_3_*). ^13^C NMR (101 MHz, DMSO-d_6_) δ: 173.6, 167.2, 164.9, 155.3, 137.5, 129.1, 127.5, 127.5, 112.8, 70.0, 54.7, 46.2, 42.9, 34.9, 30.1, 20.9.

##### 4-((2-(*tert*-Butyl)-6-methylphenoxy)methyl)-6-(4-methylpiperazin-1-yl)-1,3,5-triazin-2-amine (**10**)

Crystallization: acetone/diethyl ether, creamy solid, yield 24%, mp 177–179 °C dec. C_20_H_30_N_6_O (MW 370.49). LC/MS^+^: purity: 99.55%, t_R_ = 5.52, (ESI) *m/z* [M+H]^+^ 371.16. ^1^H NMR (500 MHz, DMSO-d_6_) δ: 7.09 (d, *J* = 7.73 Hz, 1H, Ph-*3-H*), 7.03 (d, *J* = 6.87 Hz, 1H, Ph-*5-H*), 6.97 (br s, 1H, N*H*_2_), 6.91 (t, *J* = 7.59, 1H, Ph-*4*-*H*), 6.84 (br s, 1H, N*H*_2_), 4.50 (s, 2H, C*H_2_*O), 3.70 (br s, 4H, Pp-3,5-*H*), 2.27 (s, 7H, Pp-2,6-*H* + Ph-C*H_3_*), 2.15 (s, 3H, Pp-C*H_3_*), 1.33 (s, 9H, C(C*H_3_)_3_*). ^13^C NMR (126 MHz, DMSO-d_6_) δ: 173.3, 167.4, 165.0, 156.8, 142.7, 131.6, 130.4, 125.1, 123.9, 74.4, 54.9, 46.3, 43.0, 35.3, 31.3, 17.5.

##### 4-((2,6-Diisopropylphenoxy)methyl)-6-(4-methylpiperazin-1-yl)-1,3,5-triazin-2-amine (**11**)

Crystallization: methanol, white solid, yield 47%, mp 160–163 °C. C_21_H_32_N_6_O (MW 384.53). LC/MS^+^: purity: 100%, t_R_ = 4.86, (ESI) *m/z* [M+H]^+^ 385.33. ^1^H NMR (300 MHz, DMSO-d_6_) δ: 7.09 (m, 4H, Ph-3,4,5-*H* + N*H*_2_), 6.98 (br s, 1H, N*H*_2_), 4.40 (s, 2H, OC*H_2_*), 3.72 (br s, 4H, Pp-3,5-*H*), 3. 46 (spt, *J* = 7.03 Hz, 2H, 2x C*H*(CH_3_)_2_), 2.29 (br s, 4H, Pp-2,6-*H*), 2.18 (s, 3H, Pp-C*H_3_*), 1.12 (d, *J* = 6.44 Hz, 12H, 2x CH(C*H_3_*)*_2_*). ^13^C NMR (126 MHz, DMSO-d_6_) δ: 173.0, 167.5, 165.1, 153.6, 141.9, 125.2, 124.5, 77.6, 54.9, 46.3, 42.9, 26.2, 24.5.

##### 4-((2,6-(di-*tert*-Butyl)phenoxy)methyl)-6-(4-methylpiperazin-1-yl)-1,3,5-triazin-2-amine hydrochloride (**12**)

Crystallization: acetonitrile/water, beige solid, yield 4%, mp 244 dec °C. C_23_H_36_N_6_O x HCl (MW 449.04). LC/MS^+^: purity: 96.19%, t_R_ = 6.70, (ESI) *m/z* [M+H]^+^ 413.25. ^1^H NMR (500 MHz, DMSO-d_6_) δ: 11.40 (br s, 1H, N*H*^+^), 7.47–7.73 (m, 2H, N*H_2_*), 7.24 (d, *J* = 7.73 Hz, 2H, Ph-*3,5*-*H*), 6.99 (s, 1H, Ph-*4*-*H*), 4.57–4.82 (m, 2H, OC*H_2_*), 4.53 (br s, 2H, Pp-*3,5-H_e_*), 3.32–3.54 (m, 4H, Pp-*2,6*-*H*), 3.02 (br s, 2H, Pp-*3,5-H_a_*), 2.72 (d, *J* = 2.58 Hz, 3H, Pp-C*H_3_*), 1.30–1.43 (m, 18H, 2x C(C*H_3_)_3_*).

##### 4-((2,4-(di-*tert*-Butyl)phenoxy)methyl)-6-(4-methylpiperazin-1-yl)-1,3,5-triazin-2-amine hydrochloride (**13**)

Crystallization: acetone/diethyl ether, beige solid, yield 10%, mp 284–287 °C. C_23_H_36_N_6_O x HCl (MW 449.04). LC/MS^+^: purity: 98.56%, t_R_ = 7.01, (ESI) *m/z* [M+H]^+^ 413.35. ^1^H NMR (500 MHz, DMSO-d_6_) δ: 11.58 (s, 1H, N*H*^+^), 7.31–7.90 (m, 1H, N*H*_2_), 7.21 (d, *J* = 2.58 Hz, 1H, Ph-3-*H*), 7.11 (dd, *J* = 2.43, 8.45 Hz, 1H, Ph-5-*H*), 6.83 (d, *J* = 8.59 Hz, 1H, Ph-6-*H*), 4.86 (s, 2H, OC*H_2_*), 4.50–4.75 (m, 2H, Pp-3,5-*H_a_*), 3.83–4.45 (m, 4H, Pp-2,6-*H*), 2.84–3.13 (m, 2H, Pp-3,5-*H_e_*), 2.69 (br s, 3H, Pp-C*H_3_*), 1.36 (s, 9H, C(C*H_3_)_3_*), 1.21 (s, 9H, C(C*H_3_)_3_*). ^13^C NMR (126 MHz, DMSO-d_6_) δ: 164.0, 155.0, 143.0, 137.1, 124.0, 123.6, 112.8, 68.8, 51.9, 42.5, 35.2, 34.5, 31.9, 30.2.

##### 4-(3-(2-Isopropyl-5-methylphenoxy)propyl)-6-(4-methylpiperazin-1-yl)-1,3,5-triazin-2-amine (**14**)

Crystallization: methanol, white solid, yield 6%, mp 77 °C. C_21_H_32_N_6_O (MW 384.53). LC/MS^+^: purity: 100%, t_R_ = 4.41, (ESI) *m/z* [M+H]^+^ 385.33. ^1^H NMR (500 MHz, DMSO-d_6_) δ: 6.98 (d, *J* = 7.45 Hz, 1H, Ph-4-*H*), 6.74 (br s, 1H, Ph-3-*H*), 6.65 (s, 1H, Ph-6-*H*), 6.63 (d, *J* = 7.45 Hz, 2H, N*H*_2_), 3.94 (t, *J* = 6.30 Hz, 2H, OC*H_2_*), 3.63 (s, 4H, Pp-3,5-*H*), 3.12 (spt, *J* = 6.95 Hz, 1H, C*H*(CH_3_)_2_), 2.52 (t, *J* = 7.45 Hz, 2H, C*H_2_*Tr), 2,22 (br s, 4H, Pp-2,6-*H*), 2.19 (s, 3H, Ph-C*H_3_*), 2.13 (s, 3H, Pp-C*H_3_*), 2.07 (quin *J* = 6.70 Hz, 2H, C*H_2_*CH_2_Tr), 1.08 (d, *J* = 6.87 Hz, 6H, CH(C*H_3_)_2_*). ^13^C NMR (126 MHz, DMSO-*d_6_*) δ: 177.4, 167.3, 165.0, 156.1, 136.3, 133.53, 126.0, 121.3, 112.7, 67.5, 54.9, 46.3, 42.7, 35.3, 27.0, 23.6, 23.2, 21.5.

##### 4-(3-(2-Isopropylphenoxy)propyl)-6-(4-methylpiperazin-1-yl)-1,3,5-triazin-2-amine hydrochloride (**15**)

White solid, yield 9%, mp 235 °C. C_20_H_30_N_6_O × HCl (MW 406.96). LC/MS^+^: purity: 96.60%, t_R_ = 4.08, (ESI) *m/z* [M+H]^+^ 371.30.^1^H NMR (500 MHz, DMSO-d_6_) δ: 11.99 (br s, 1H, N*H*^+^), 8.83 (br s, 1H, N*H*_2_), 7.91 (br s, 1H, N*H*_2_), 7.20-7.02 (m, 2H, Ph-3,4-*H*), 6.93-6.78 (m, 2H, Ph-5,6-*H*), 4.46-4.81 (m, 2H, Pp-3,5-*H_e_*), 4.01 (br s, 2H, OC*H_2_*), 3.30-3.66 (m, 4H, Pp-2,6-*H*) 2.92-3.18 (m, 3H, Pp-3,5-*H_a_* + C*H*(CH_3_)_2_), 2.83 (br s, 2H, C*H_2_*Tr), 2.70 (br s, 3H, Pp-C*H_3_*), 2.19 (br s, 2H, C*H_2_*CH_2_Tr), 1.09 (d, *J* = 6.30 Hz, 6H, CH(C*H_3_)_2_*). ^13^C NMR (126 MHz, DMSO-d_6_) δ: 173.7, 169.8, 161.9, 157.8, 155.9, 136.5, 127.2, 126.2, 121.1, 112.0, 67.0, 59.9, 51.7, 42.4, 31.8, 26.6, 25.6, 23.2.

##### 4-(3-(4-Isopropylphenoxy)propyl)-6-(4-methylpiperazin-1-yl)-1,3,5-triazin-2-amine (**16**)

White solid, yield 49%, mp 92 °C. C_20_H_30_N_6_O (MW 370.50). LC/MS^+^: purity: 98.43%, t_R_ = 3.83, (ESI) *m/z* [M+H]^+^ 371.23. ^1^H NMR (500 MHz, DMSO-d_6_) δ: 7.06 (d, *J* = 8.02 Hz, 2H, Ph-3,5-*H*), 6.77 (d, *J* = 7.45 Hz, 2H, Ph-2,6-*H*), 6.72-6.62 (m, 2H, N*H_2_*), 3.92 (br s, 2H, OC*H_2_*), 3.63 (br s, 4H, Pp-3,5-*H*), 2.81-2.70 (m, 1H, C*H*(CH_3_)_2_), 2.52-2.43 (m, 2H, OCH_2_CH_2_C*H_2_*), 2,22 (br s, 4H, Pp-2,6-*H*), 2,12 (br s, 3H, Pp-C*H_3_*), 2.05-1.97 (br s, 2H, OCH_2_C*H_2_*CH_2_), 1.10 (d, *J* = 6.30 Hz, 6H, CH(C*H_3_)_2_*). ^13^C NMR (126 MHz, DMSO-d_6_) δ: 177.4, 167.3, 165.0, 157.2, 140.7, 127.6, 126.2, 114.7, 67.4, 54.9, 46.3, 42.9, 35.0, 33.1, 26.9, 24.6.

##### 4-(4-Methylpiperazin-1-yl)-6-(3-(m-tolyloxy)propyl)-1,3,5-triazin-2-amine (**17**)

Crystallization: acetonitrile, white solid, yield 12%, mp 114–117 °C. C_19_H_26_N_6_O (MW 342.45). LC/MS^+/-^: purity: 100%, t_R_ = 3.24, (ESI) m/z [M+H]^+^ 343.30. ^1^H NMR (500 MHz, DMSO-d_6_) δ: 7.09 (s, 1H, Ph-5-H), 6.57–6.85 (m, 5H, NH_2_ + Ph-2,4,6-H), 3.94 (t, *J* = 6.30 Hz, 2H, OCH_2_), 3.63 (br s, 4H, Pp-3,5-H), 2.48 (s, 2H, CH_2_Tr), 2.19–2.29 (m, 7H, Pp-2,6-H + PhCH_3_), 2.14 (s, 3H, Pp-CH_3_), 2.02 (s, 2H, OCH_2_CH_2_). ^13^C NMR (126 MHz, DMSO-d_6_) δ: 177.4, 167.3, 165.0, 159.1, 139.4, 129.7, 121.6, 115.5, 112.0, 67.3, 54.9, 46.3, 42.9, 35.0, 26.9, 21.7.

##### 4-(4-Methylpiperazin-1-yl)-6-(3-(o-tolyloxy)propyl)-1,3,5-triazin-2-amine (**18**)

White solid, yield 43%, mp 83 °C. C_18_H_26_N_6_O (MW 342.45). LC/MS^+^: purity: 100%, t_R_ = 3.00, (ESI) *m/z* [M+H]^+^ 343.25. ^1^H NMR (500 MHz, DMSO-d_6_) δ: 7.06 (d, *J* = 7.45 Hz, 2H, Ph-3,5-*H*), 6.83 (d, *J* = 8.02 Hz, 1H, Ph-6-*H*), 6.80-6.62 (m, 3H, Ph-4-*H*, N*H*_2_), 3.96 (t, *J* = 6.01 Hz, 2H, OC*H_2_*), 3.63 (br s, 4H, Pp-3,5-*H*), 2.52 (t, *J* = 7.45 Hz, 2H, C*H_2_*Tr), 2.22 (br s, 4H, Pp-2,6-*H*), 2,13 (br s, 3H, Pp-C*H_3_*), 2.08 (s, 3H, PhC*H_3_*), 2.06-2.03 (m, 2H, C*H_2_*CH_2_Tr). ^13^C NMR (126 MHz, DMSO-d_6_) δ: 177.4, 167.3, 165.0, 157.1, 130.8, 127.4, 126.2, 120.5, 111.7, 67.5, 54.9, 46.3, 42.9, 35.2, 27.0, 16.4.

##### 4-(4-(2-Isopropyl-5-methylphenoxy)butyl)-6-(4-methylpiperazin-1-yl)-1,3,5-triazin-2-amine hydrochloride (**19**)

White solid, yield 9%, mp 239 °C. C_22_H_34_N_6_O × HCl (MW 435.02). LC/MS^+^: purity: 100%, t_R_ = 4.71, (ESI) *m/z* [M+H]^+^ 399.35. ^1^H NMR (500 MHz, DMSO-d_6_) δ: 11.79 (br s, 1H, N*H*^+^), 8.69 (br s, 1H, N*H*_2_), 7.80 (br s, 1H, N*H*_2_), 7.00 (d, *J* = 8.02 Hz, 1H, Ph-3-*H*), 6.70 (s, 1H, Ph-6-*H*), 6.65 (d, *J* = 7.45 Hz, 1H, Ph-4-*H*), 4.78-4.50 (m, 2H, OC*H_2_*), 3.94 (t, *J* = 6.01 Hz, 4H, Pp-3,5-*H*), 3.14 (spt, *J* = 6.87 Hz, 1H, C*H*(CH_3_)_2_), 3.03 (br s, 2H, C*H_2_*Tr), 2.73–2.65 (m, 7H, PhC*H_3_* + Pp-2,6-*H*), 2.21 (s, 3H, Pp-C*H_3_*), 1.90-1.82 (m, 2H, OCH_2_C*H_2_*), 1.81–1.74 (m, 2H, C*H_2_*CH_2_Tr), 1.09 (d, *J* = 6.87 Hz, 6H, CH(C*H_3_)_2_*). ^13^C NMR (126 MHz, DMSO-d_6_) δ: 170.3, 162.1, 156.0, 136.7, 133,4, 126.0, 121.4, 67.4, 51.7, 42.4, 34.3, 28.6, 26.7, 23.2, 21.5.

##### 4-(4-(2-Isopropylphenoxy)butyl)-6-(4-methylpiperazin-1-yl)-1,3,5-triazin-2-amine (**20**)

White solid, yield 41%, mp 59 °C. C_21_H_32_N_6_O (MW 384.53). LC/MS^+^: purity: 95.85%, t_R_ = 4.23, (ESI) *m/z* [M+H]^+^ 385.33. ^1^H NMR (500 MHz, DMSO-d_6_) δ: 7.11 (d, *J* = 7.45 Hz, 1H, Ph-3-*H*), 7.07 (t, *J* = 7.73 Hz, 1H, Ph-5-*H*), 6.84 (t, *J* = 8.02 Hz, 2H, Ph-4,6-*H*), 6.73-6.68 (m, 2H, N*H_2_*), 3.92 (t, *J* = 6.01 Hz, 2H, OC*H_2_*), 3.64 (br s, 4H, Pp-3,5-*H*), 3.19 (spt, *J* = 6.87 Hz, 1H, C*H*(CH_3_)_2_), 2.40 (t, *J* = 7.16 Hz, 2H, C*H_2_*Tr), 2.22 (br s, 4H, Pp-2,6-*H*), 2.12 (s, 3H, Pp-C*H_3_*), 1.84-1.66 (m, 4H, OCH_2_(C*H_2_*)_2_ CH_2_), 1.10 (d, *J* = 6.87 Hz, 6H, CH(C*H_3_)_2_*). ^13^C NMR (126 MHz, DMSO-d_6_) δ: 177.8, 167.3, 165.0, 156.2, 136.5, 127.2, 126.2, 120.8, 111.9, 67.7, 54.9, 46.3, 42.8, 38.2, 29.1, 26.9, 24.1, 23.0.

##### 4-(4-Methylpiperazin-1-yl)-6-(4-(m-tolyloxy)butyl)-1,3,5-triazin-2-amine (**21**)

Crystallization: acetonitrile/water, white solid, yield 23%, mp 99–101 °C. C_19_H_28_N_6_O (MW 356.47). LC/MS^+/−^: purity: 100%, t_R_ = 3.75, (ESI) *m/z* [M+H]^+^ 357.47. ^1^H NMR (500 MHz, DMSO-d_6_) δ: 7.09 (t, *J* = 8.02 Hz, 1H, Ph-5-*H*), 6.58–6.84 (m, 5H, Ph-2,4,6-*H* + N*H_2_*), 3.90 (t, *J* = 6.16 Hz, 2H, OC*H_2_*), 3.63 (br s, 4H, Pp-3,5-*H*), 2.38 (t, *J* = 7.30 Hz, 2H, C*H_2_*Tr), 2.18–2.27 (m, 7H, Pp-2,6-*H* + PhC*H_3_*), 2.14 (s, 3H, Pp-C*H_3_*), 1.63–1.80 (m, 4H, (C*H_2_*)_2_). ^13^C NMR (DMSO-d_6_, 126 MHz) δ: 177.8, 167.3, 165.0, 159.2, 139.4, 129.7, 121.6, 115.6, 111.9, 67.5, 54.9, 46.3, 42.9, 38.3, 29.0, 24.0, 21.6.

##### 4-(5-(2-Isopropyl-5-methylphenoxy)pentyl)-6-(4-methylpiperazin-1-yl)-1,3,5-triazin-2-amine (**22**)

Crystallization: acetonitrile/water, white solid, yield 6%, mp 75 °C dec. C_23_H_36_N_6_O (MW 412.58). LC/MS^+/−^: purity: 100%, t_R_ = 5.64, (ESI) *m/z* [M+H]^+^ 413.38. ^1^H NMR (500 MHz, DMSO-d_6_) δ: 6.97 (d, *J* = 7.73 Hz, 1H, Ph-3-*H*), 6.53–6.77 (m, 4H, Ph-4,6-*H* + N*H_2_*), 3.88 (t, *J* = 6.16 Hz, 2H, OC*H_2_*), 3.62 (br s, 4H, Pp-3,5-*H*), 3.10 (spt, *J* = 6.83, 1H, C*H*(CH_3_)_2_), 2.34 (t, *J* = 7.45 Hz, 2H, C*H_2_*Tr), 2.17–2.25 (m, 7H, Pp-2,6-*H* + PhC*H_3_*), 2.13 (s, 3H, Pp-C*H_3_*), 1.58–1.76 (m, 4H, OCH_2_C*H_2_* + C*H_2_*CH_2_Tr), 1.42 (quin, *J* = 7.52 Hz, 2H, O(CH_2_)_2_C*H_2_*), 1.06 (d, *J* = 6.87 Hz, 6H, CH(C*H_3_*)*_2_*). ^13^C NMR (126 MHz, DMSO-d_6_) δ: 177.8, 167.3, 165.0, 156.2, 136.3, 133.5, 126.0, 121.2, 112.7, 67.7, 54.9, 46.3, 42.8, 38.6, 29.2, 27.1, 26.7, 26.0, 23.1, 21.5.

##### 4-(5-(2-Isopropylphenoxy)pentyl)-6-(4-methylpiperazin-1-yl)-1,3,5-triazin-2-amine (**23**)

Crystallization: acetonitrile/water, white solid, yield 28%, mp 63–65 °C. C_22_H_34_N_6_O (MW 398.55). LC/MS^+/−^: purity: 100%, t_R_= 4.95, (ESI) *m/z* [M+H]^+^ 399.37. ^1^H NMR (500 MHz, DMSO-d_6_) δ: 7.01–7.16 (m, 2H, Ph-3,4-*H*), 6.77–6.88 (m, 2H, Ph-5,6-*H*), 6.51-6.77 (br s, 2H, N*H_2_*), 3.89 (t, *J* = 6.16 Hz, 2H, OC*H_2_*), 3.62 (br s, 4H, Pp-3,5-*H*), 3.16 (def spt, 1H, C*H*(CH_3_)*_2_*), 2.34 (t, *J* = 7.45 Hz, 2H, C*H_2_*Tr), 2.22 (br s, 4H, Pp-2,6-*H*), 2.12 (s, 3H, Pp-C*H_3_*), 1.59–1.78 (m, 4H, OCH_2_C*H_2_* + C*H_2_*CH_2_Tr), 1.43 (quin, *J* = 7.45 Hz, 2H, O(CH_2_)_2_C*H_2_*), 1.09 (d, *J* = 6.87 Hz, 6H, CH(C*H_3_*)*_2_*). ^13^C NMR (126 MHz, DMSO-d_6_) δ: 177.8, 167.3, 165.0, 156.3, 136.5, 127.2, 126.2, 120.8, 111.9, 67.7, 54.9, 46.3, 42.9, 38.6, 29.2, 27.1, 26.9, 26.0, 23.0.

##### 4-(4-Methylpiperazin-1-yl)-6-(5-(m-tolyloxy)pentyl) 1,3,5-triazin-2-amine (**24**)

Crystallization: acetonitrile/water, white solid, yield 17%, mp 83–85 °C. C_20_H_30_N_6_O (MW 370.50). LC/MS^+/−^: purity: 100%, t_R_= 4.20, (ESI) *m/z* [M+H]^+^ 371.33. ^1^H NMR (500 MHz, DMSO-d_6_) δ: 7.05–7.13 (m, 1H, Ph-5-*H*), 6.57–6.79 (m, 5H, Ph-2,4,6-*H* + N*H_2_*), 3.87 (t, *J* = 6.44 Hz, 2H, OC*H_2_*), 3.63 (br s, 4H, Pp-3,6-*H*), 2.33 (t, *J* = 7.45 Hz, 2H, C*H_2_*Tr), 2.19–2.27 (m, 7H, Pp-3,5-*H* + PhC*H_3_*), 2.13 (s, 3H, Pp-C*H_3_*), 1.65 (m, 4H, OCH_2_C*H_2_* + C*H_2_*CH_2_Tr), 1.33–1.43 (m, 2H, O(CH_2_)_2_C*H_2_*). ^13^C NMR (126 MHz, DMSO-d_6_) δ: 177.9, 167.3, 165.0, 159.2, 139.4, 129.7, 121.6, 115.6, 111.9, 67.6, 54.9, 46.3, 42.9, 38.6, 29.1, 27.2, 25.9, 21.6.

##### 4-(6-(2-Isopropyl-5-methylphenoxy)hexyl)-6-(4-methylpiperazin-1-yl)-1,3,5-triazin-2-amine (**25**)

Crystallization: acetonitrile, white solid, yield 5%, mp 59 °C dec. C_24_H_38_N_6_O (MW 426.61). LC/MS^+/-^: purity: 100%, t_R_= 6.46, (ESI) *m/z* [M+H]^+^ 427.18. ^1^H NMR (500 MHz, DMSO-d_6_) δ: 6.98 (d, *J* = 7.45 Hz, 1H, Ph-3-*H*), 6.55–6.77 (m, 4H, Ph-4,6-*H* + N*H_2_*), 3.88 (t, *J* = 6.16 Hz, 2H, OC*H_2_*), 3.63 (br s, 4H, Pp-3,5-*H*), 3.13 (spt, *J* = 6.92, 1H, C*H*(CH_3_)*_2_*), 2.32 (t, *J* = 7.59 Hz, 2H, C*H_2_*Tr), 2.17–2.25 (m, 7H, Pp-2,6-*H* + PhC*H_3_*), 2.13 (s, 3H, Pp-C*H_3_*), 1.55–1.74 (m, 4H, OCH_2_C*H_2_* + C*H_2_*CH_2_Tr), 1.42 (quin, *J* = 7.37 Hz, 2H, O(CH_2_)_2_C*H_2_*), 1.26–1.35 (m, 2H, C*H_2_*CH_2_Tr), 1.08 (d, *J* = 6.87 Hz, 6H, CH(C*H_3_*)*_2_*). ^13^C NMR (126 MHz, DMSO-d_6_) δ: 178.0, 167.3, 165.0, 156.2, 136.3, 133.5, 126.0, 121.2, 112.8, 67.7, 54.9, 46.3, 42.8, 38.6, 29.3, 29.0, 27.4, 26.7, 26.0, 23.1, 21.5.

### 3.2. In Vitro Pharmacological Studies

Radioligand binding assays were used to determine the affinity of the synthesized compounds for human serotonin 5-HT6R, 5-HT_2A_R, 5-HT_7_R and dopamine D_2_R, which were stably expressed in HEK293 cells or CHOk1 cells (5-HT_2A_R). This was done as described previously [12].

### 3.3. PAMPA Assay

Evaluation of cell membrane permeation of compound **3** was performed on PAMPA Plate System Gentest™ plates from Corning (Tewksbury, MA, USA), as described previously [16]. The permeability coefficient P_e_ was calculated by formulas described in the literature [26] and compared with a high-permeable caffeine (CFN).

### 3.4. Hepatotoxicity

The hepatoma cell line HepG2 (ATCC^®^ HB-8065TM) was used to assess the hepatotoxicity of the compounds according to previously described protocols [16]. The CellTiter 96^®^ AQueous Non-Radioactive Cell Proliferation Assay was purchased from Promega (Madison, WI, USA). Compound **3** was tested in two independent experiments in triplicate at seven concentrations (0.78, 1.56, 3.125, 6.25, 12.5, 25 and 50 µM) for 72 h.

### 3.5. Water Solubility Determination

The water solubility of the selected compounds was determined using UV spectroscopy following previously described methods [22,23]. The calibration curves were determined using a series of dilutions for each compound. Stock solutions in methanol, with the concentration of 1 mg/mL, were further diluted to produce seven solutions with concentrations in the range of 10^−3^–10^−1^ mg/mL. Saturated solutions of tested compounds were prepared by suspending each compound (10 mg) in H_2_O (2 mL). The suspensions were refluxed for 5 min, then left overnight at 20 °C and filtered off using a Macherey–Nagel MN 619 de filter. Individual filtrates were diluted in methanol (from 10 to 160 times) and analyzed using UV spectroscopy as a solution in methanol/water (90% *v/v*). The concentrations of saturated solutions were calculated using MS Excel by linear regression of the two vicinal points of the calibration curves and multiplication by the dilution rate.

More information about this experiment and calibration curves for compounds can be found in the Supplementary Material S1.

### 3.6. Crystal Structures of Compounds ***3*** and ***3-SA***

Crystallization attempts to obtain suitable crystals for X-ray analysis for hydrochloride of compound **3** failed. Therefore, we prepared other salts for compound **3** using various organic acids. Suitable crystals of compound **3** were obtained from a mixture of propan-2-ol and decane (1:1, *v*:*v*) and for salt of compound **3** with succinic acid (SA) from ethyl acetate, in both cases through slow evaporation of the solvent at room temperature.

Data for single crystals were collected using the XtaLAB Synergy-S diffractometer, equipped with the Cu (1.54184 Å) Kα radiation source and graphite monochromator. The structures were solved via direct methods using a SIR-2014 program [27], and all non-hydrogen atoms were refined anisotropically using weighted, full-matrix least squares on F^2^. Refinement and further calculations were carried out using the SHELXL program [28]. Hydrogen atoms bonded to carbons were included in the structure at idealized positions and were refined using a riding model with U_iso_(H) fixed at 1.5 U_eq_(C) for methyl groups and 1.2 U_eq_(C) for the other hydrogen atoms. Hydrogen atoms attached to nitrogen atoms were found from the difference Fourier map and refined without any restraints.

During the structure refinement of 3-SA, some strong residual electron density peaks were present. Because any attempts to refine this as a chemically rational particle have given non-satisfactory results, the solvent mask option, implemented in Olex2 as an alternative to SQUEEZE, was used [29]. This left in unit cells a cavity with a volume of about 222.5 Å^3^ in the structure containing about 54 electrons (respectively 111.25 Å^3^ and 27 e for ASU). This allowed us to assume, statistically, that the cavity was filled with about one strongly disordered molecule of ethyl acetate (respectively, 1/2 molecule for ASU), which was used as a solvent during crystallization.

For molecular graphics the MERCURY [30] program was used.

Crystallographic data:

**3**: 2C_19_H_29_N_6_O, M_r_ = 714.96, wavelength 1.54184 Å, crystal size = 0.04 × 0.07 × 0.36 mm^3^, monoclinic, space group Pn, a = 17.4856(3) Å, b = 6.0535(1) Å, c = 18.3357(4) Å, β = 98.738(2)°, V = 1918.06(7) Å^3^, Z = 2, T = 100(2) K, 16519 reflections collected, 6293 unique reflections (R_int_ = 0.0353), R1 = 0.0356, wR2 = 0.0962 [I > 2σ(I)], R1 = 0.0368, wR2 = 0.0973 [all data].

**3-SA**: C_19_H_30_N_6_O^+^·1.5C_6_H_8_O_6_¯, M_r_ = 533.60, wavelength 1.54184 Å, crystal size = 0.04 × 0.15 × 0.47 mm^3^, triclinic, space group $P\bar{1}$, a = 9.0639(1) Å, b = 12.7333(1) Å, c = 14.7101(2) Å, α = 108.461(1)°, β = 92.440(1)°, γ = 110.419(1)°, V = 1486.64(3) Å^3^, Z = 2, T = 100(2) K, 32337 reflections collected, 5544 unique reflections (R_int_ = 0.0337), R1 = 0.0343, wR2 = 0.0967 [I > 2σ(I)], R1 = 0.0363, wR2 = 0.0984 [all data].

CCDC 2222391-2222392 contain the supplementary crystallographic data. These data can be obtained free of charge from The Cambridge Crystallographic Data Centre via www.ccdc.cam.ac.uk/data_request/cif (accessed since 26 November 2022).

## 4. Conclusions

In summary, three series of novel 4-(piperazin-1-yl)-1,3,5-triazine derivatives (mono-substituted, di-substituted and with a longer linker) were designed and synthesized.

The compounds obtained showed variable affinity for 5-HT_6_R. Some of them, especially from series 1, were characterized by a very high interaction strength with this receptor (K_i_ < 150 nM). Compound **3** (4-(2-*tert*-butylphenoxy)-6-(4-methylpiperazin-1-yl)-1,3,5-triazin-2-amine), which had a high affinity for 5-HT_6_R and selectivity for other receptors tested (5-HT_2A_R_,_ 5-HT_7_R and D_2_R), was selected for further studies. In vitro evaluation proved its good ability for passive permeability and moderate hepatotoxicity. Solubility tests showed that converting a free base into salts increased its solubility, but the amount depended on the type of salt into which the compound was made. The best solubility was that of hydrochloride, which was not surprising. The results also showed that the type of salt in which the compound was carried out influenced not only its solubility but also its binding to the investigated receptors, and this strength was usually lower than for the free base alone. For such 1,3,5-triazine derivatives, hydrochloride seemed to be the most favorable salt, as this formulation did not affect the affinity for 5-HT_6_R as much, but this finding still needs further research.

Moreover, crystallographic studies of compounds **3** and **3-SA** showed that the piperazine ring always adopted a chair conformation. In the crystal structure of compound **3,** two molecules of this compound were presented. In one of them, there was an unusual position (axial) of the methyl substituent at the piperazine ring, while in the other molecule (as in the structure of the salt of **3-SA**), the methyl group at the piperazine occupied an equatorial position.

Furthermore, the modifications introduced to the lead **MST4** led also to promising multi-target structures acting on several targets simultaneously, e.g., three targets (5-HT_6_R, 5-HT_2A_R and D_2_R, such as in compound **25** (K_i_ = 78 nM vs. K_i_ = 364 nM vs. K_i_ = 149 nM, respectively)). Compounds acting on two such targets, i.e., 5-HT_6_R and D_2_R [31] or 5-HT_6_R and 5-HT_2A_R [32], are described in the literature, but such three-target ligands are not yet available.

To sum up, the work carried out shows that 1,3,5-triazine derivatives are promising structures for further research. New modifications may in the future lead not only to highly potent and selective 5-HT_6_R ligands, but also to multi-targeted compounds, with a potential for more effective therapeutic use, e.g., in the treatment of AD.

## Figures and Tables

**Table 1:** Structures and biological activity of tested compounds **1**–**7** (series 1) ^1^.

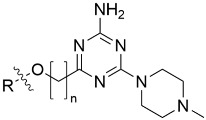						
**K_i_ [nM]**						
**Compound**	**R**	**n**	**5-HT_6_R**	**5-HT_2A_R**	**5-HT_7_R**	**D_2_R**
**MST4**	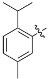	1	11 ^a^	430 ^a^	11,950 ^a^	1094 ^a^
**1 ^a^**	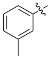	1	207 ^a^	2274 ^a^	10,660 ^a^	740 ^a^
**2**	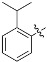	1	21	1267	16,260	nt ^b^
**3**	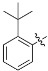	1	13	2183	6336	1616
**4**	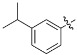	1	41	2613	5726	1790
**5**	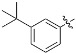	1	108	3112	15,460	nt ^b^
**6**	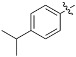	1	416	248	10,130	nt ^b^
**7**	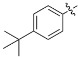	1	150	549	4138	3134
Reference ligands			Olanzapine 7 ^a^	Aripiprazole 21 ^c^	Clozapine 62 ^a^	Olanzapine 9 ^a^

^1^ Tested experimentally in the radioligand binding assay; binding affinity, K_i_, expressed as the average of at least two independent experiments. Used radioligands: [^3^H]-LSD (5-HT_6_R), [^3^H]-ketanserin (5-HT_2A_R), [^3^H]-5-CT (5-HT_7_R) and [^3^H]-raclopride (D_2_R); ^a^ data from Ali et al. [15]; ^b^ nt: not tested; ^c^ data from Kucwaj-Brysz et al. [8].

**Table 2:** Structures and biological activity of tested compounds **8**–**13** (series 2) ^1^.

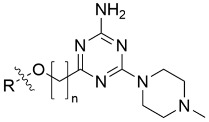						
			**K_i_ [nM]**			
**Compound**	**R**	**n**	**5-HT_6_R**	**5-HT_2A_R**	**5-HT_7_R**	**D_2_R**
**MST4**	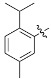	1	11 ^a^	430 ^a^	11,950 ^a^	1094 ^a^
**8**	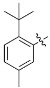	1	6	1245	15,090	1570
**9**	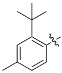	1	48	1933	10,920	2295
**10**	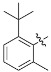	1	236	nt ^b^	16,570	nt ^b^
**11**	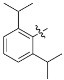	1	825	7799	33,580	nt ^b^
**12**	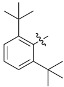	1	379	nt ^b^	8085	nt ^b^
**13**	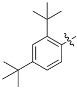	1	162	760	8475	2743

^1^ Tested experimentally in the radioligand binding assay in human cells stably transfected with a proper receptor; binding affinity, K_i_, expressed as the average of at least two independent experiments. Used radioligands: [^3^H]-LSD (5HT_6_R), [^3^H]-ketanserin (5HT_2A_R), [^3^H]-5-CT (%HT_7_R) and [^3^H]-raclopride (D_2_R); ^a^ data from Ali et al. [15]; ^b^ nt: not tested.

**Table 3:** Structures and biological activity of tested compounds **14**–**25** (series 3) ^1^.

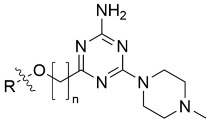						
**K_i_ [nM]**						
**Compound**	**R**	**n**	**5-HT_6_R**	**5-HT_2A_R**	**5-HT_7_R**	**D_2_R**
**MST4**	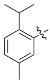	1	11 ^a^	430 ^a^	11,950 ^a^	1094 ^a^
**14**		3	412	1695	6660	1592
**15**	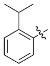	3	221	489	8870	2429
**16**	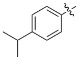	3	1107	1169	5125	704
**17**	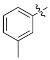	3	1455	3846	30,250	2600
**18**	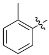	3	574	1445	14,880	1760
**19**	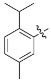	4	142	687	638	3198
**20**	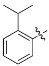	4	43	677	665	5689
**21**	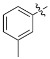	4	559	952	6254	612
**22**	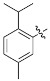	5	282	709	771	837
**23**	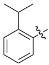	5	189	597	3431	196
**24**	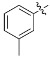	5	644	854	1886	481
**25**	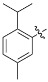	6	78	364	1031	149

^1^ Tested experimentally in the radioligand binding assay in human cells stably transfected with a proper receptor; binding affinity, K_i_, expressed as the average of at least two independent experiments. Used radioligands: [^3^H]-LSD (5-HT_6_R), [^3^H]-ketanserin (5-HT_2A_R), [^3^H]-5-CT (5-HT_7_R) and [^3^H]-raclopride (D_2_R); ^a^ data from Ali et al. [15].

**Table 4:** Result of BBB penetration of compound **3** evaluated in PAMPA assay.

	Study a		Study b ^b^	
	3	CFN ^a^	MST4	CFN ^a^
*Pe* (×10^−6^ cm/s) ± SD	4.7 ± 0.2	9.8 ± 1.8	12.3 ± 2.0	15.1 ± 0.4

^a^ Caffeine; ^b^ data from ref [16].

**Table 5:** Affinity results for the serotonin 5-HT_6_ and 5-HT_7_ receptors of salts of compound **3**.

	K_i_ [nM] ^a^	
	5-HT_6_R	5-HT_7_R
**3**	13	6336
**3-HCl**	35	15,500
**3-SA**	135	3674

^a^ Tested experimentally in the radioligand binding assay in human cells stably transfected with 5-HT_6_ or 5-HT_7_ receptors; binding affinity, K_i_, expressed as the average of at least two independent experiments. Used radioligands: [^3^H]-LSD (5-HT_6_R) and [^3^H]-5-CT (5-HT_7_R).

**Table 6:** Water solubility of tested compounds.

Compound	Solubility in Water [mg/mL]	Solubility in Water [µmol/L]
**3**	0.0160	45
**3-HCl**	6.8017	17,310
**3-SA**	0.3529	618

**Table 7:** The comparison of selected bond lengths [Å] and torsion angles [°] in compounds **3** and **3-SA**.

	Compound 3 Molecule A	Compound 3 Molecule B	Compound 3-SA
C2-N6	1.345 (3)	1.347 (3)	1.329 (2)
C4-N2	1.355 (3)	1.355 (3)	1.364 (1)
C6-C12-O1-C13	168.4 (2)	−174.5 (2)	−172.2 (2)
C12-O1-C13-C18	30.4 (3)	−19.2 (3)	−6.0 (2)

## Data Availability

Data are available from the authors upon request.

## Supplementary Materials

The following supporting information can be downloaded at https://www.mdpi.com/article/10.3390/molecules28031108/s1: Supplementary Materials S1 (synthesis of intermediates, ^1^H NMR of selected intermediates, solubility determination with calibration curves) and S2 (^1^H NMR and ^13^C NMR spectra of compounds **1**–**25**).

## Sample Availability

Samples of the compounds **1**–**25** could be available from the authors.
