# Supplementary material for: New Triazine Derivatives as Serotonin 5-HT6 Receptor Ligands
Source: Molecules. 2023 Jan 22;28(3):1108. doi: 10.3390/molecules28031108 (PMC9919591; doi:10.3390/molecules28031108)
Supplement: Supplementary file 1 [file molecules-28-01108-s001.zip › Supplementary material S2.pdf]

New triazine derivatives as serotonin 5-HT$_6$ receptor ligands

Dorota Łażewska[1*], Małgorzata Więcek[1], Grzegorz Satała[2], Paulina Chałupnik[1], Ewa Żesławska[3], Ewelina Honkisz-Orzechowska[1], Monika Tarasek[1], Gniewomir Latacz[1], Wojciech Nitek[4], Ewa Szymańska[1] and Jadwiga Handzlik[1*]

[1]Department of Technology and Biotechnology of Drugs, Faculty of Pharmacy, Jagiellonian University Medical College in Kraków, Medyczna 9, 30-688 Krakow, Poland;
[2]Department of Medicinal Chemistry, Maj Institute of Pharmacology, Polish Academy of Sciences, Smętna 12, 31-343 Kraków, Poland;
[3] Institute of Biology, Pedagogical University of Kraków, Podchorążych 2, 30-084 Kraków, Poland;
[4]Faculty of Chemistry, Jagiellonian University in Kraków, Gronostajowa 2, 30-387 Kraków, Poland

* Correspondence: dorota.lazewska@uj.edu.pl (D.Ł.); j.handzlik@uj.edu.pl (J.H.)

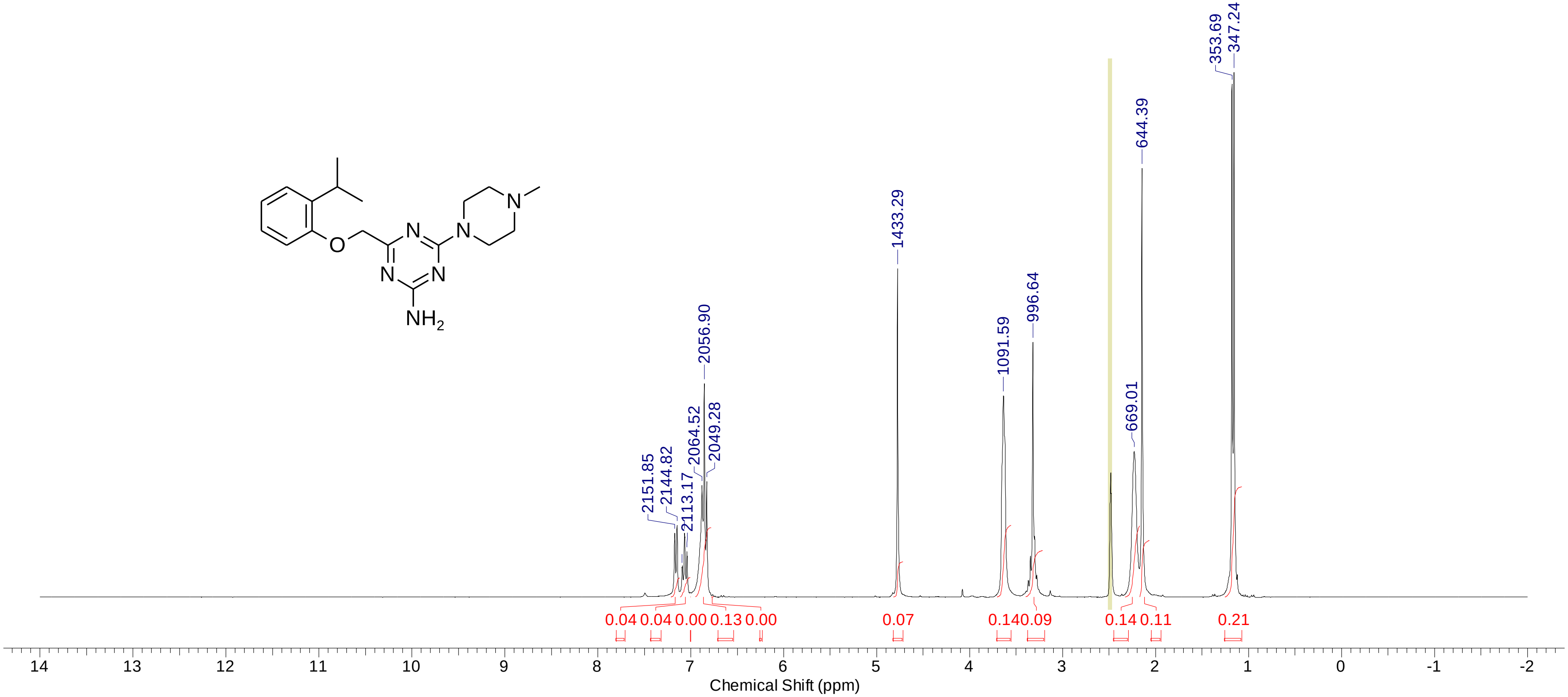

^{1}H NMR spectrum of 4-((2-isopropylphenoxy)methyl)-6-(4-methylpiperazin-1-yl)-1,3,5-triazin-2-amine (**2**)

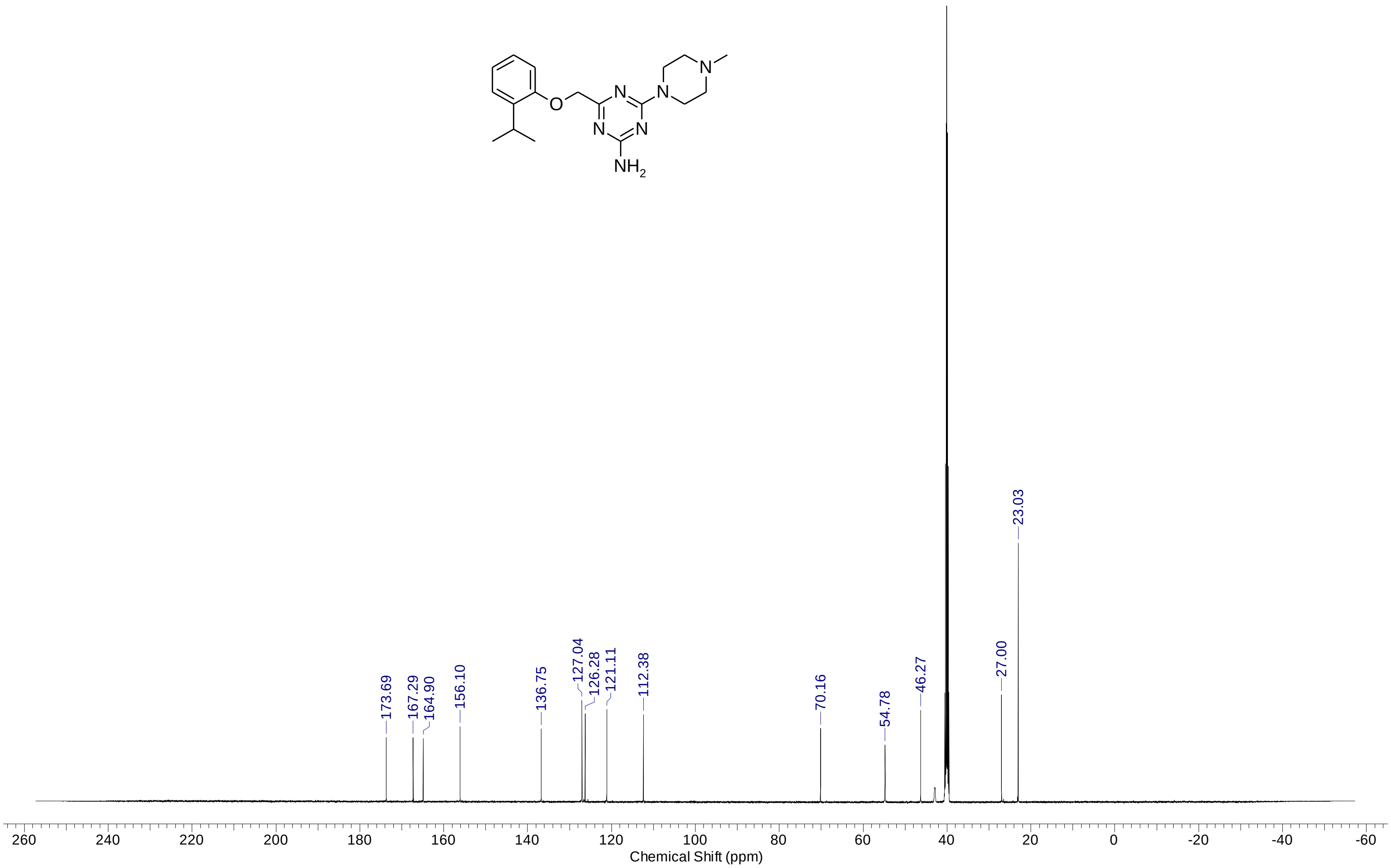

^{13}C NMR spectrum of 4-((2-isopropylphenoxy)methyl)-6-(4-methylpiperazin-1-yl)-1,3,5-triazin-2-amine (**2**)

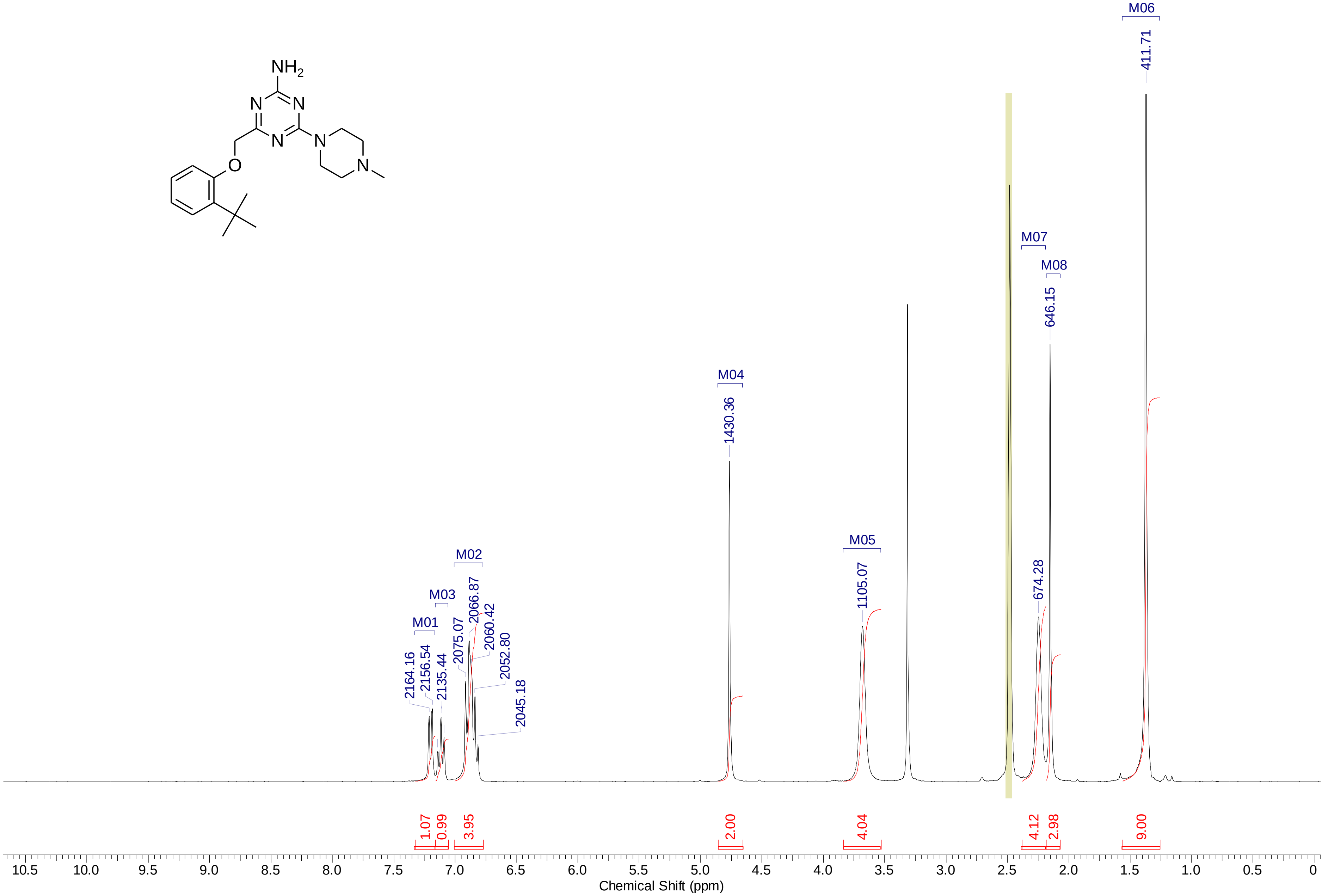

^{1}H NMR spectrum of 4-((2-*tert*-butylphenoxy)methyl)-6-(4-methylpiperazin-1-yl)-1,3,5-triazin-2-amine (**3**)

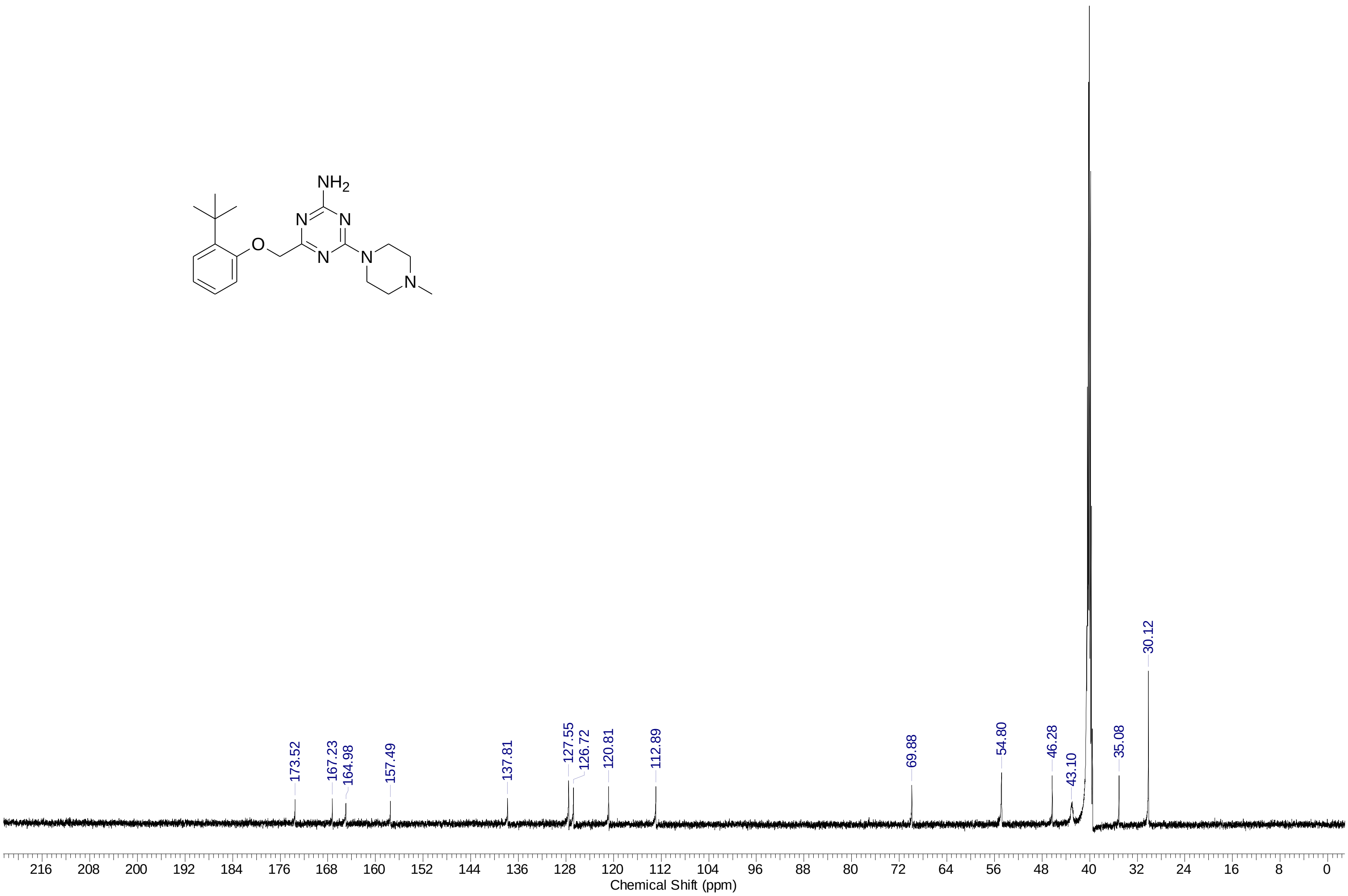

^{13}C NMR spectrum of 4-((2-*tert*-butylphenoxy)methyl)-6-(4-methylpiperazin-1-yl)-1,3,5-triazin-2-amine (**3**)

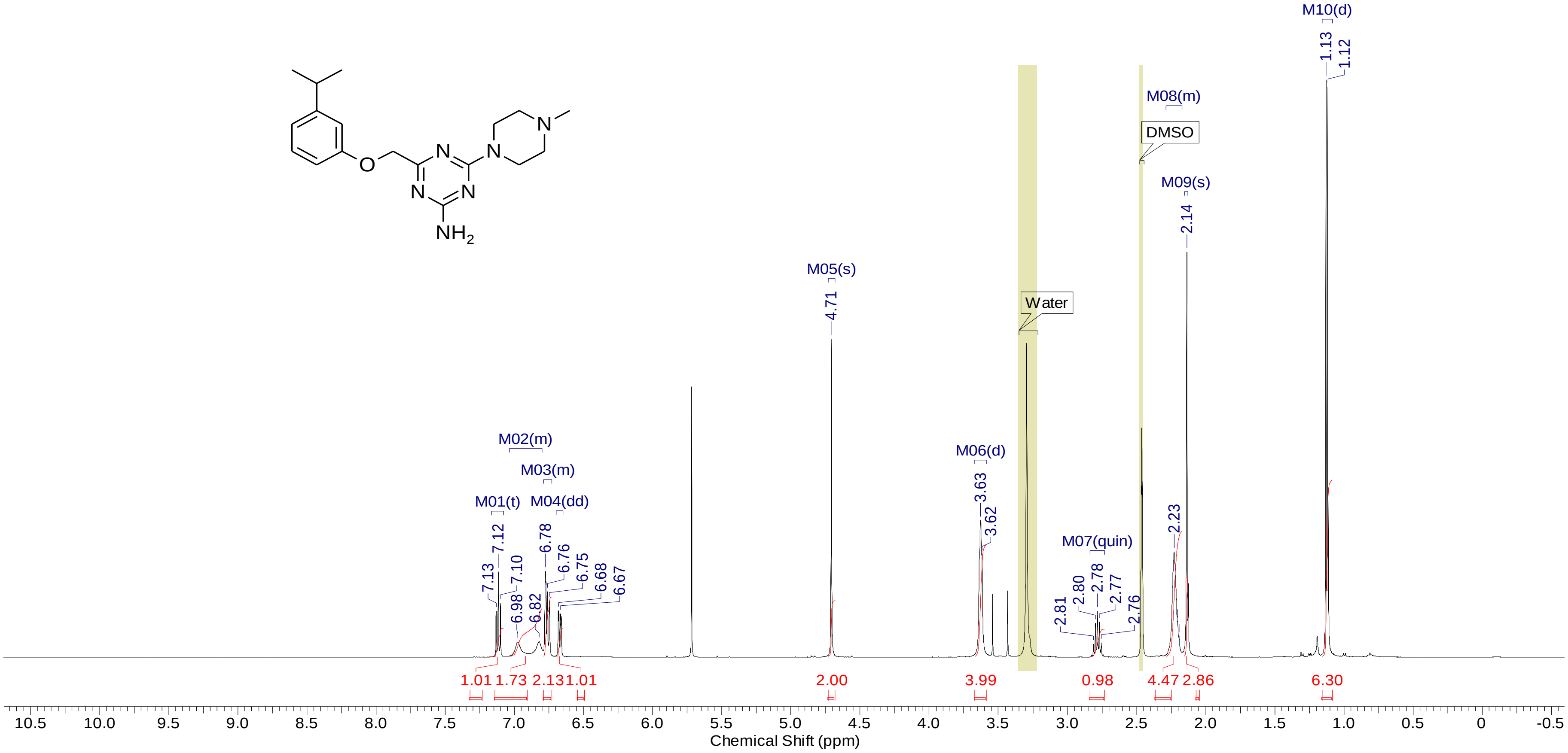

^{1}H NMR spectrum of 4-((3-isopropylphenoxy)methyl)-6-(4-methylpiperazin-1-yl)-1,3,5-triazin-2-amine (**4**)

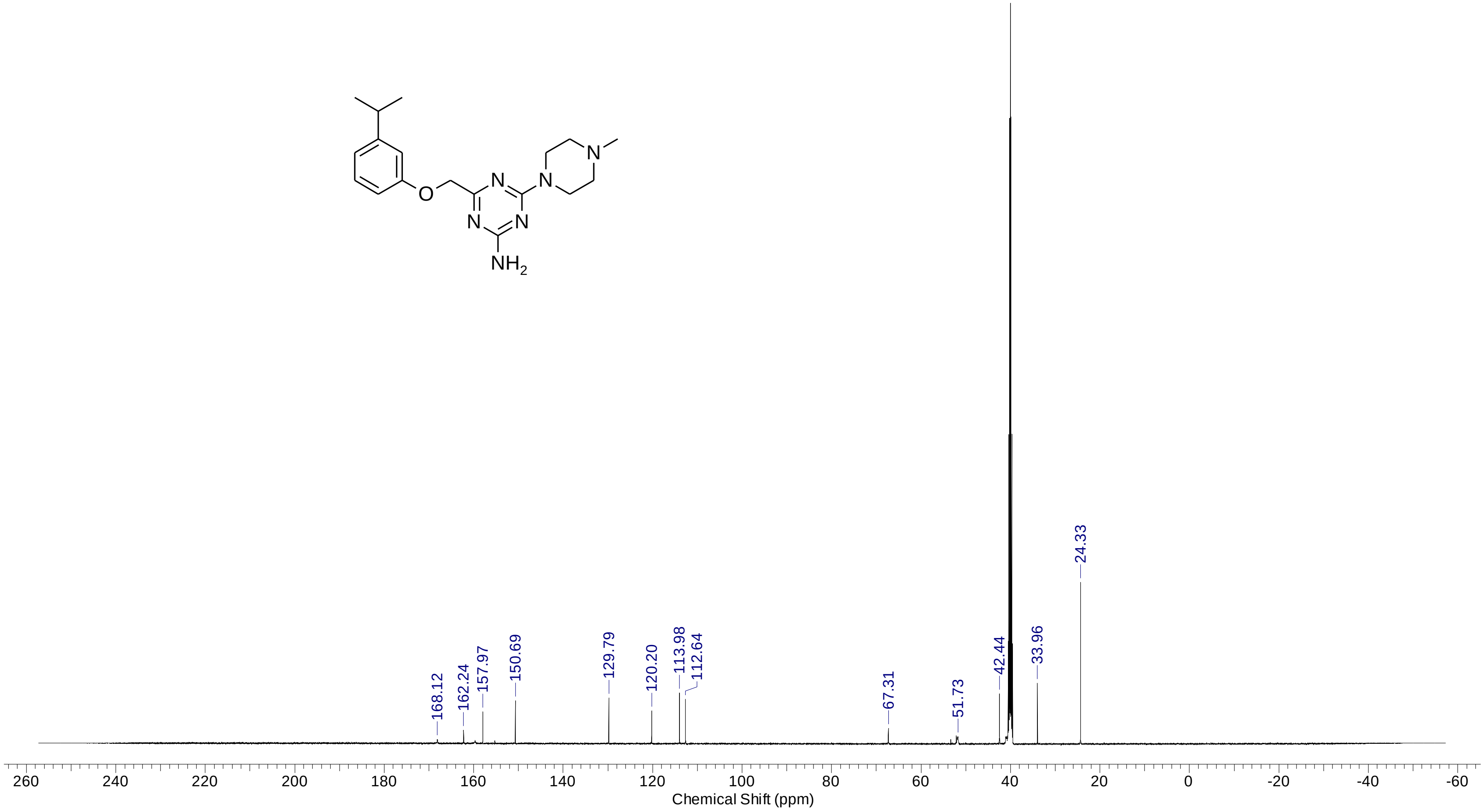

^{13}C NMR spectrum of 4-((3-isopropylphenoxy)methyl)-6-(4-methylpiperazin-1-yl)-1,3,5-triazin-2-amine (**4**)

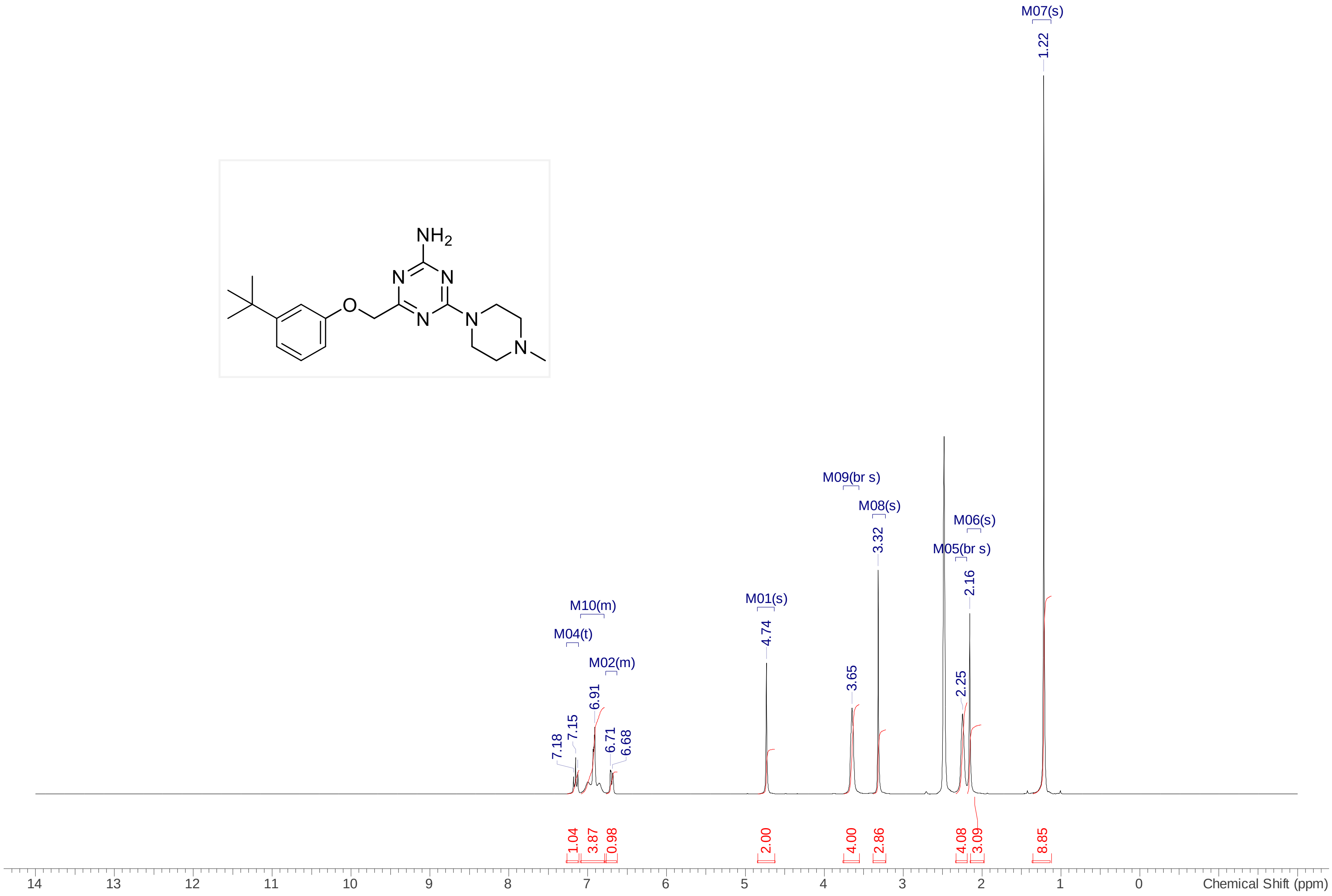

^{1}H NMR spectrum of 4-((3-*tert*-butylphenoxy)methyl)-6-(4-methylpiperazin-1-yl)-1,3,5-triazin-2-amine (**5**)

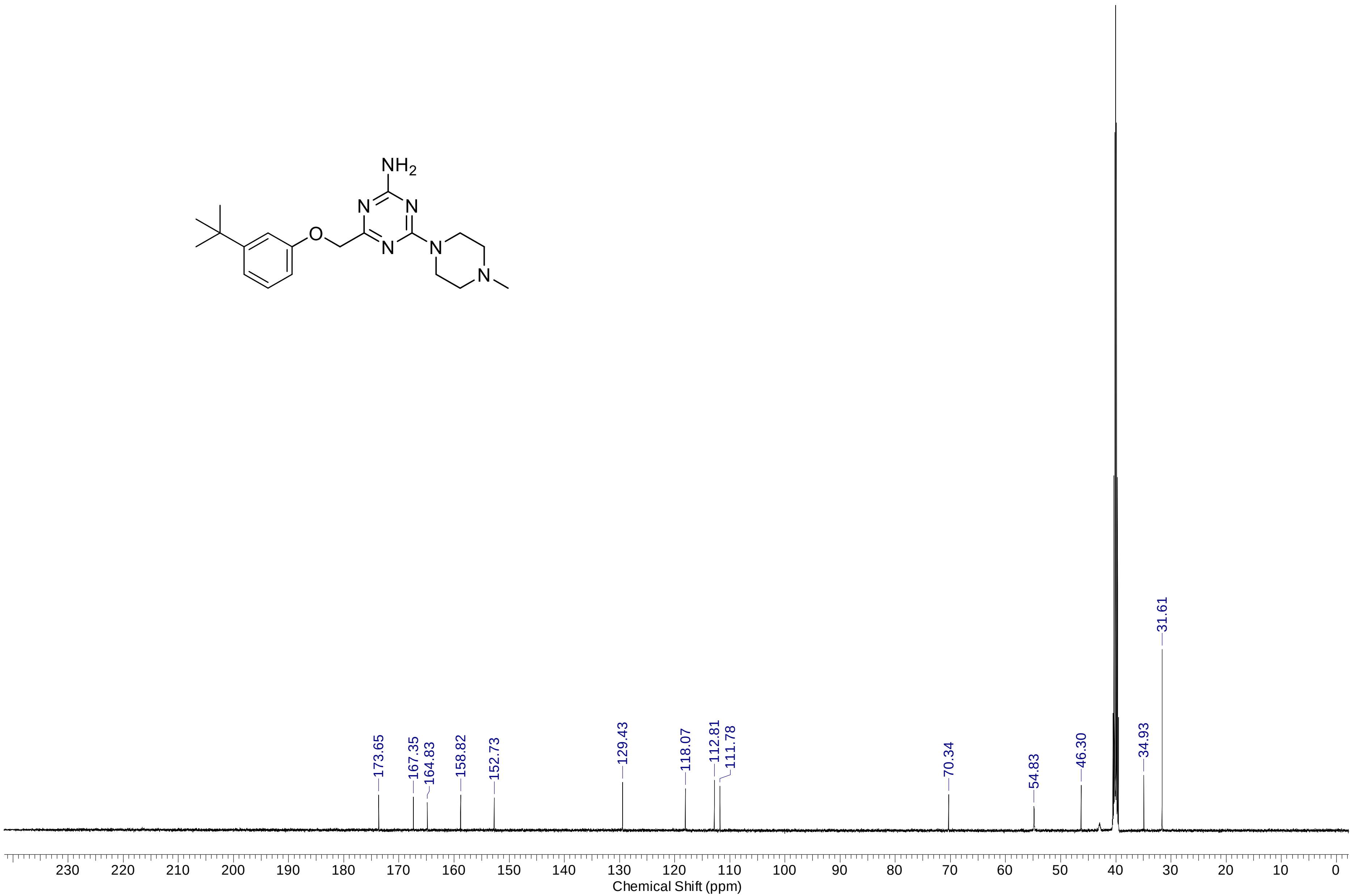

^{13}C NMR spectrum of 4-((3-*tert*-butylphenoxy)methyl)-6-(4-methylpiperazin-1-yl)-1,3,5-triazin-2-amine (**5**)

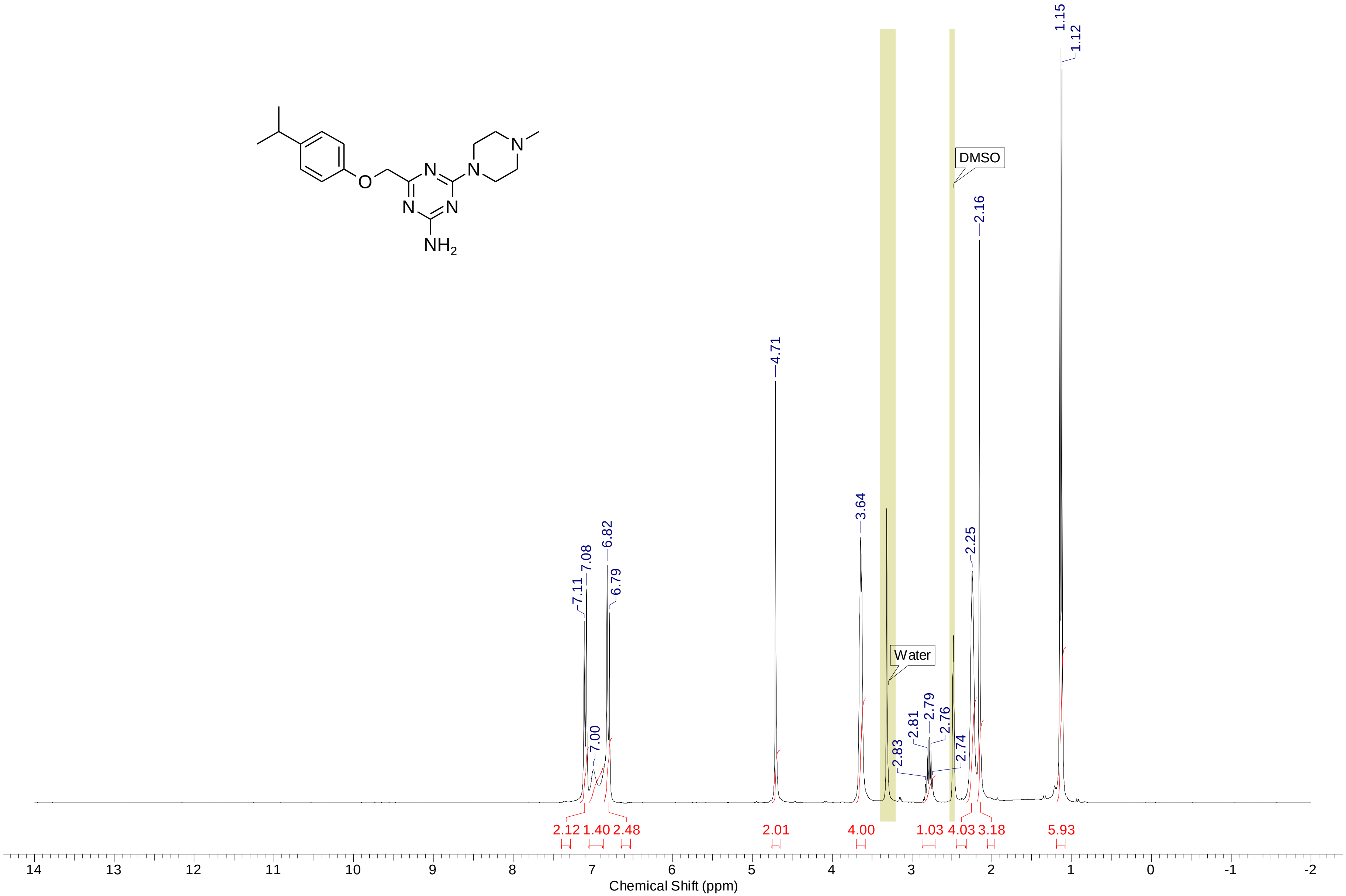

^{1}H NMR spectrum of 4-((4-isopropylphenoxy)methyl)-6-(4-methylpiperazin-1-yl)-1,3,5-triazin-2-amine (**6**)

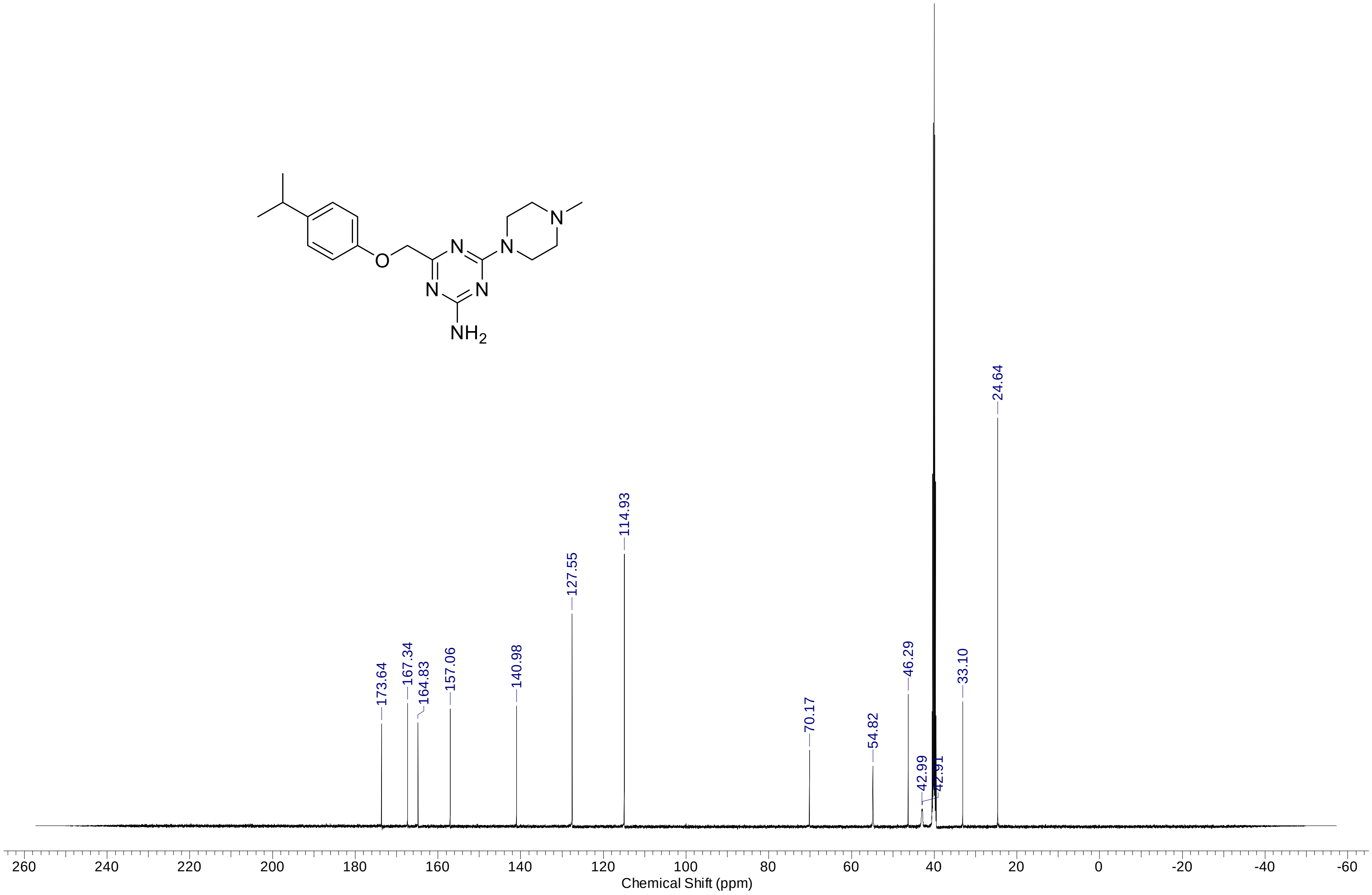

^{13}C NMR spectrum of 4-((4-isopropylphenoxy)methyl)-6-(4-methylpiperazin-1-yl)-1,3,5-triazin-2-amine (**6**)

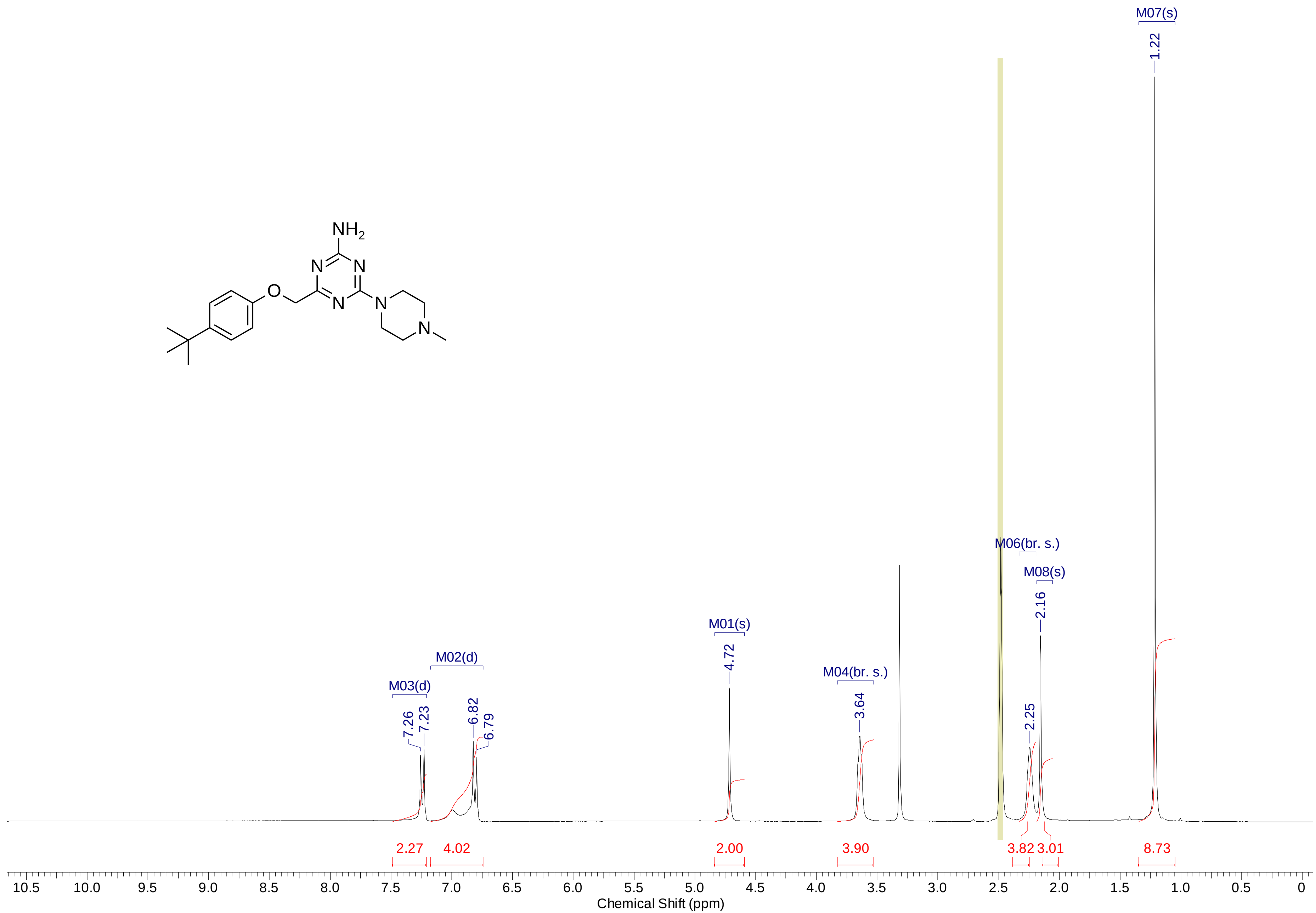

^{1}H NMR spectrum of 4-((4-*tert*-butylphenoxy)methyl)-6-(4-methylpiperazin-1-yl)-1,3,5-triazin-2-amine (**7**)

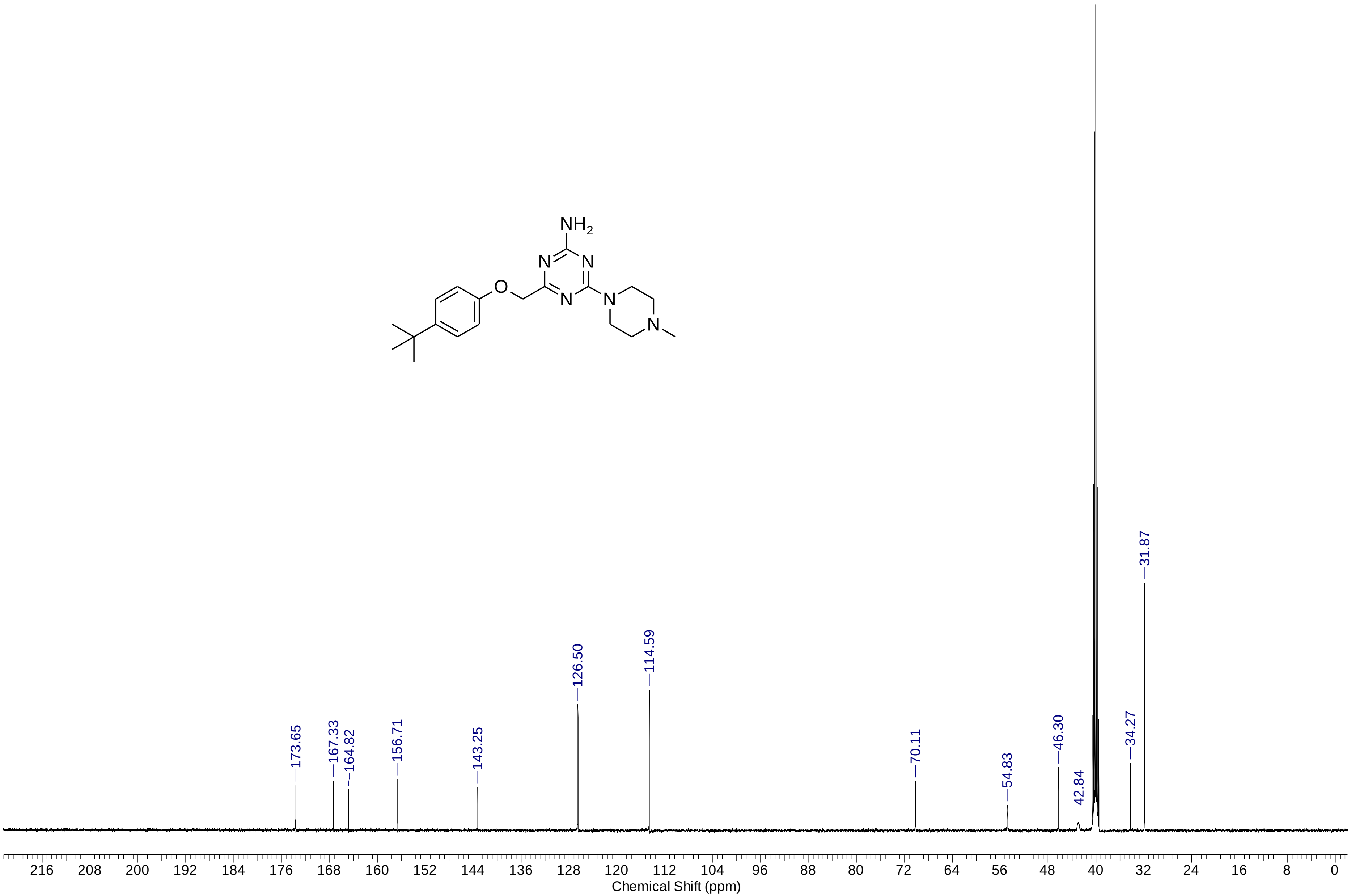

^{13}C NMR spectrum of 4-((4-*tert*-butylphenoxy)methyl)-6-(4-methylpiperazin-1-yl)-1,3,5-triazin-2-amine (**7**)

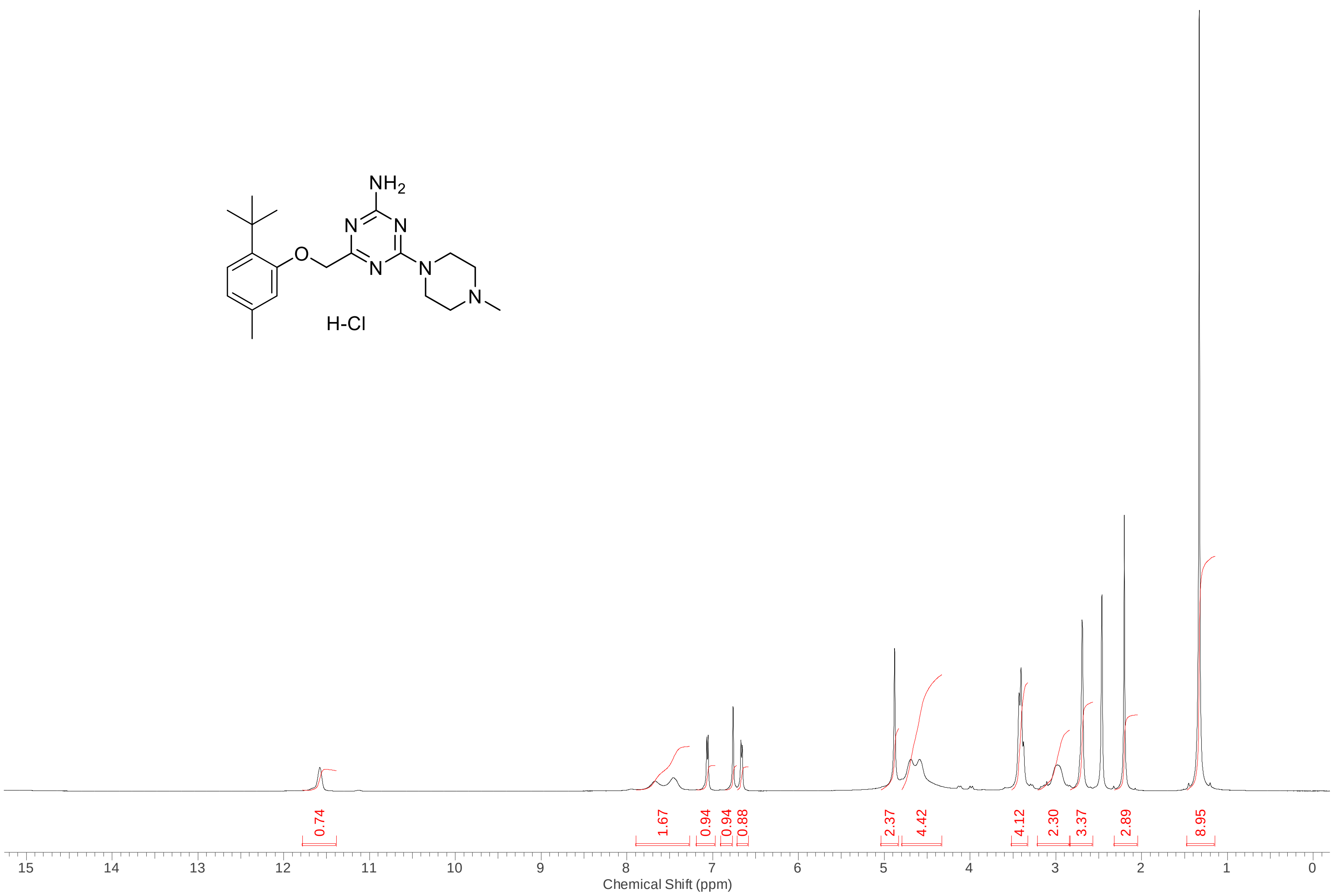

^{1}H NMR spectrum of 4-((2-*tert*-butyl-5-methylphenoxy)methyl)-6-(4-methylpiperazin-1-yl)-1,3,5-triazin-2-amine hydrochloride (**8**)

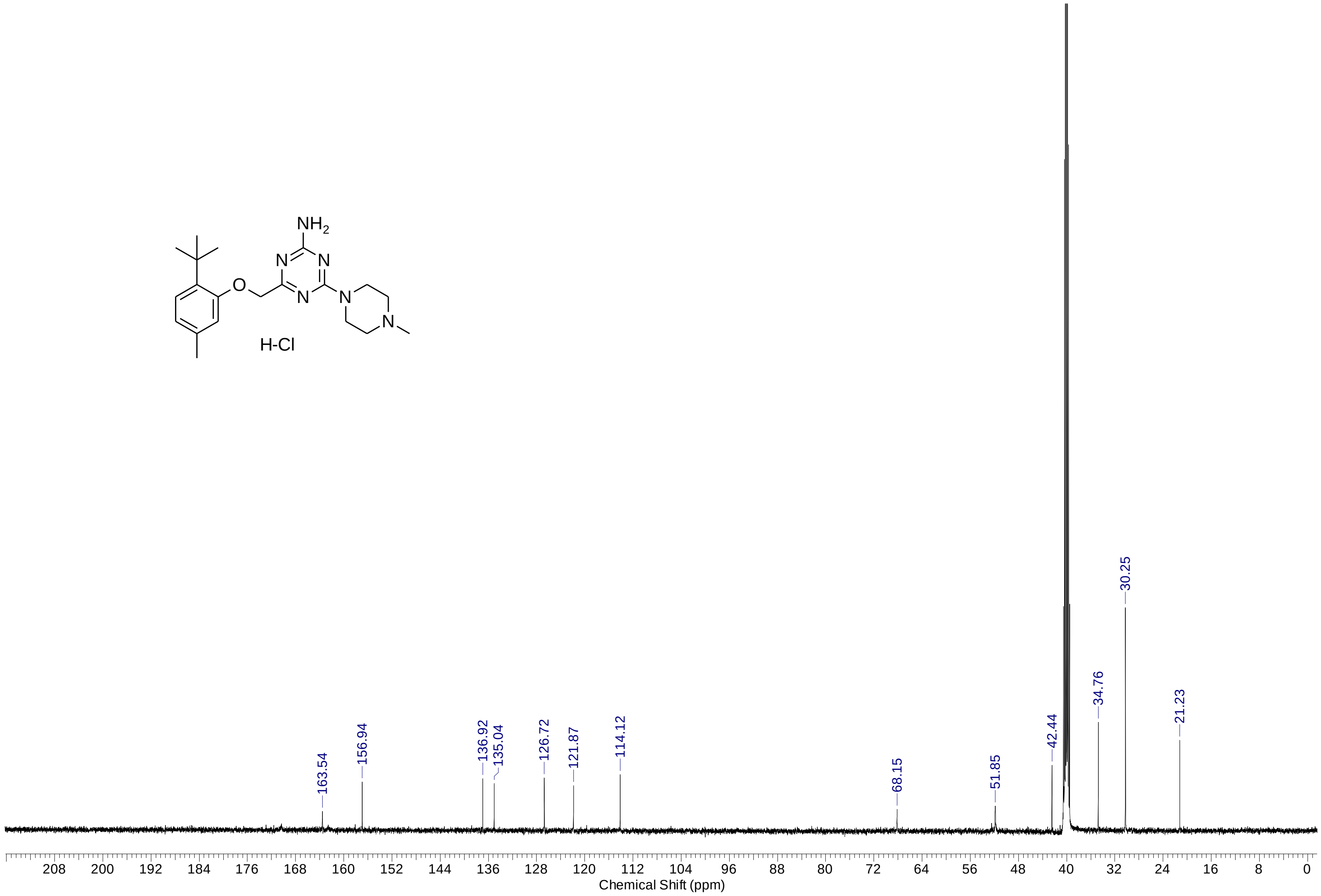

^{13}C NMR spectrum of 4-((2-*tert*-butyl-5-methylphenoxy)methyl)-6-(4-methylpiperazin-1-yl)-1,3,5-triazin-2-amine hydrochloride (**8**)

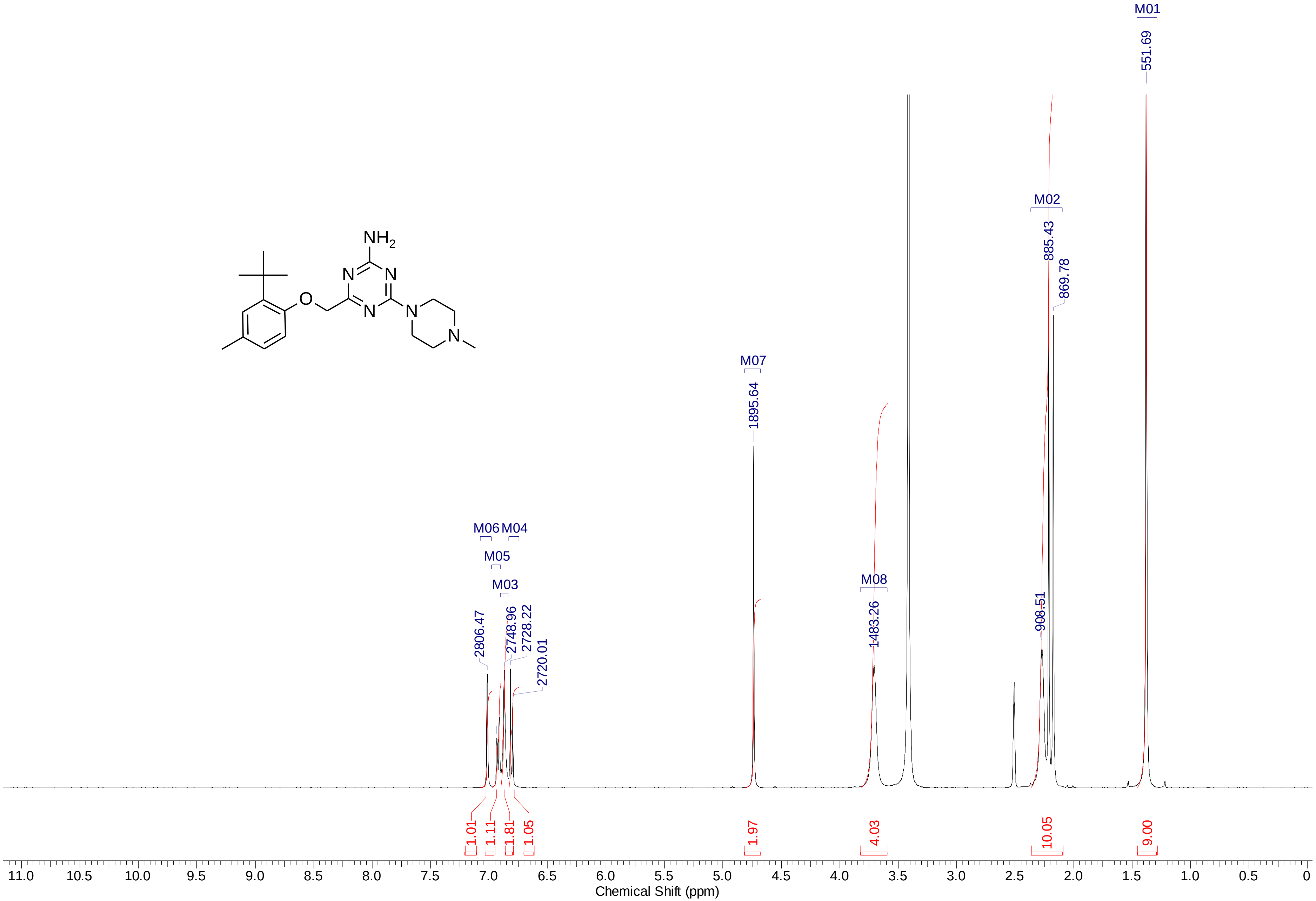

^{1}H NMR spectrum of 4-((2-*tert*-butyl-4-methylphenoxy)methyl)-6-(4-methylpiperazin-1-yl)-1,3,5-triazin-2-amine (**9**)

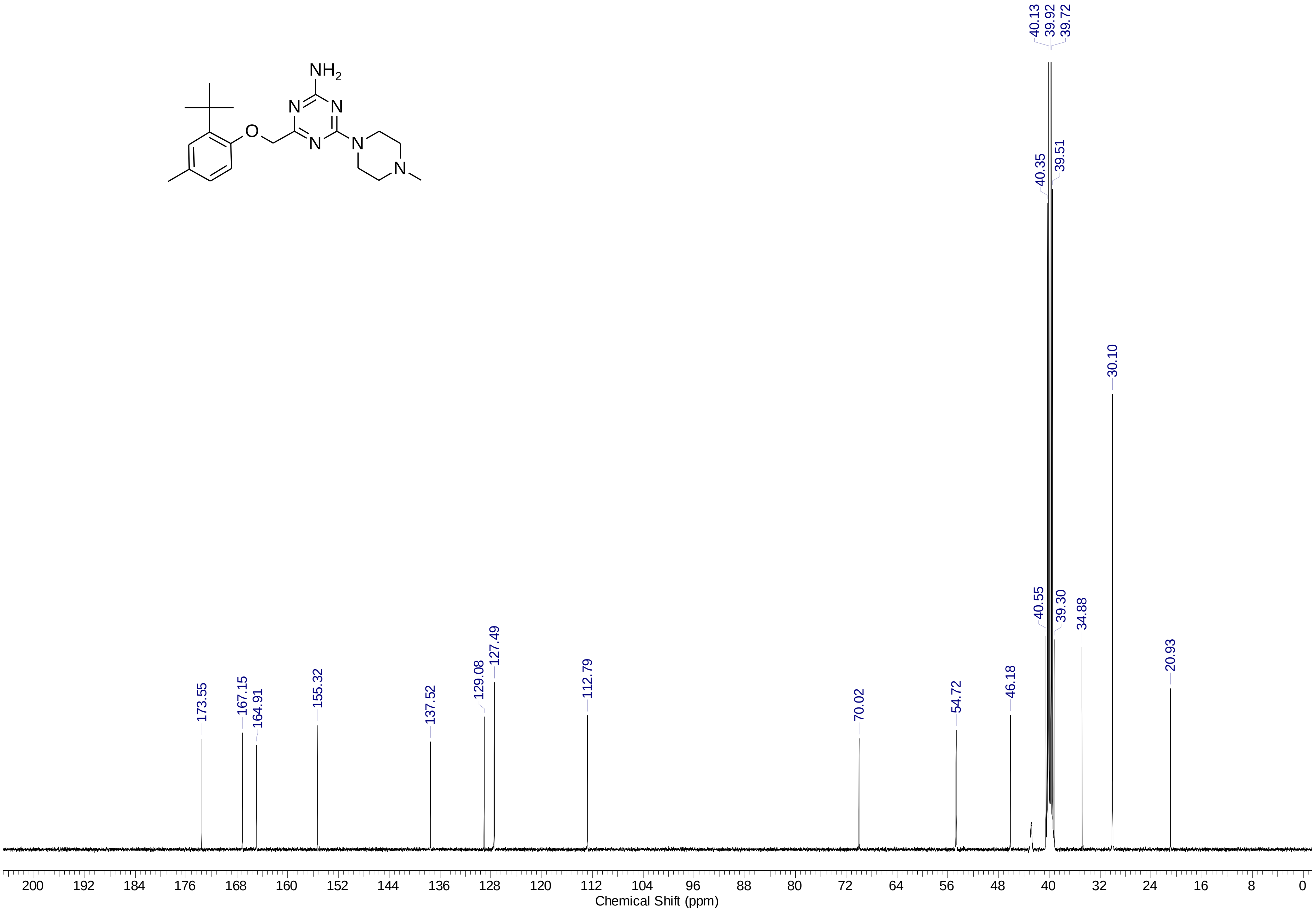

^{13}C NMR spectrum of 4-((2-*tert*-butyl-4-methylphenoxy)methyl)-6-(4-methylpiperazin-1-yl)-1,3,5-triazin-2-amine (**9**)

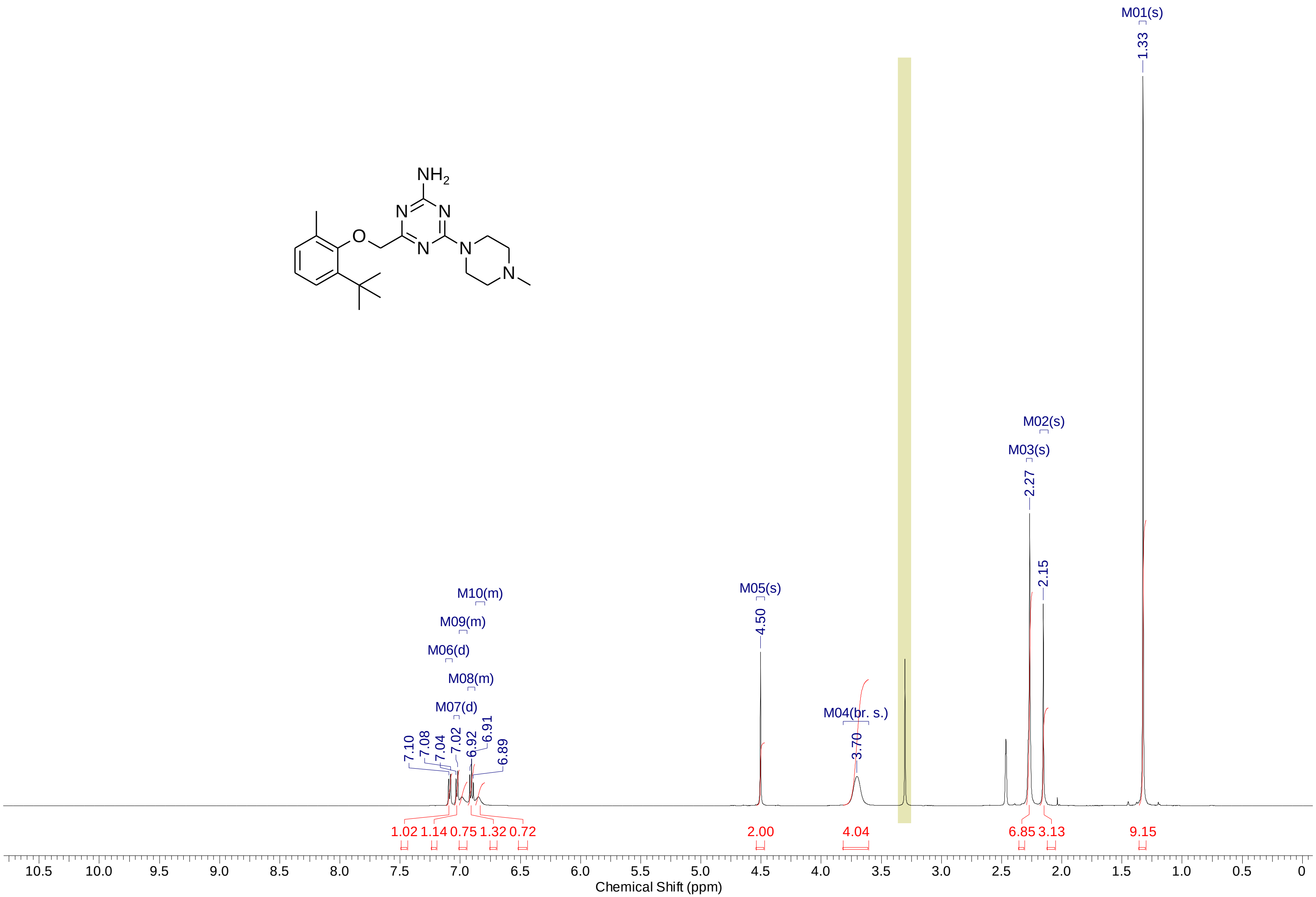

^{1}H NMR spectrum of 4-((2-(*tert*-butyl)-6-methylphenoxy)methyl)-6-(4-methylpiperazin-1-yl)-1,3,5-triazin-2-amine (**10**)

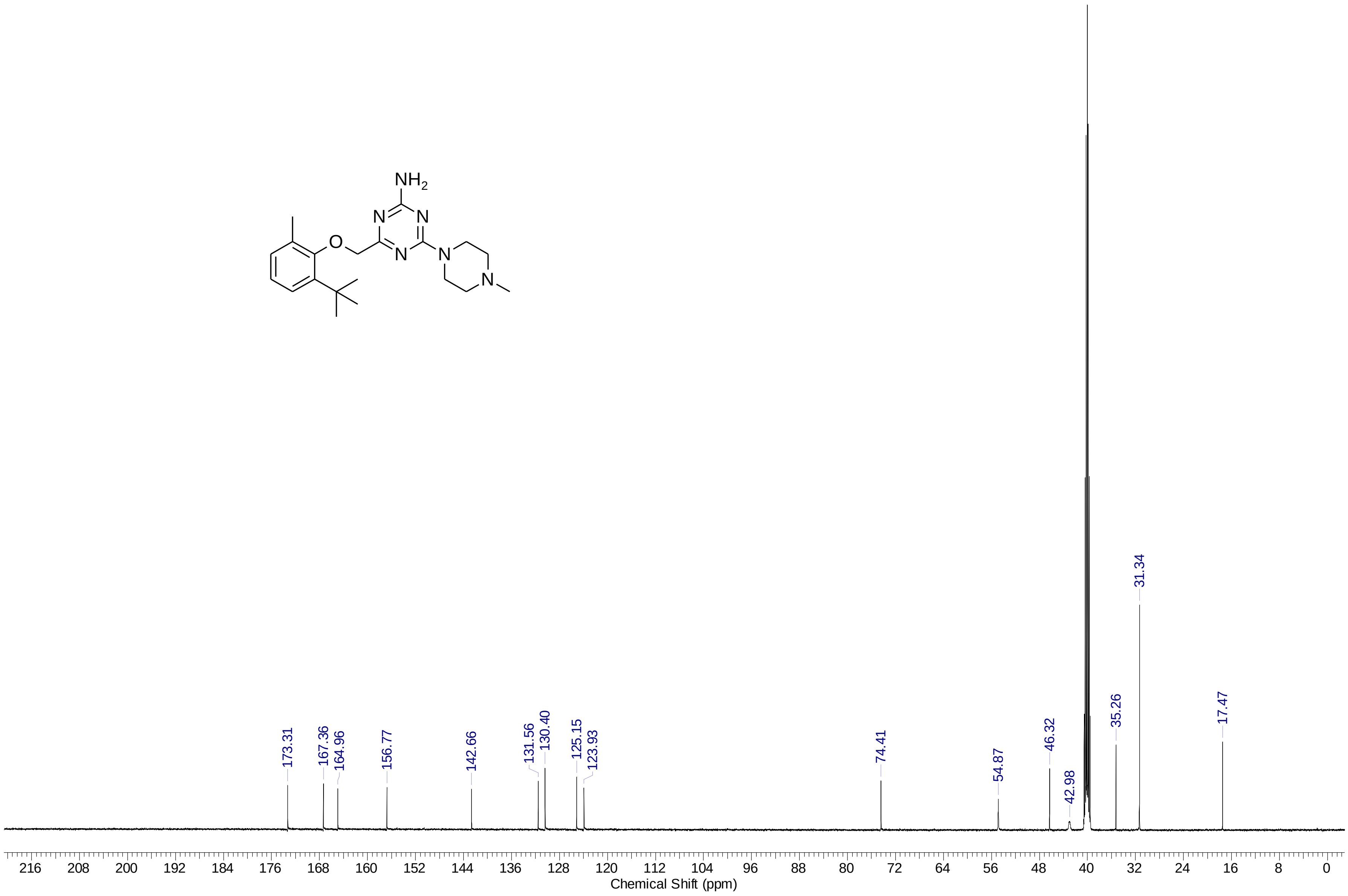

^{13}C NMR spectrum of 4-((2-(*tert*-butyl)-6-methylphenoxy)methyl)-6-(4-methylpiperazin-1-yl)-1,3,5-triazin-2-amine (**10**)

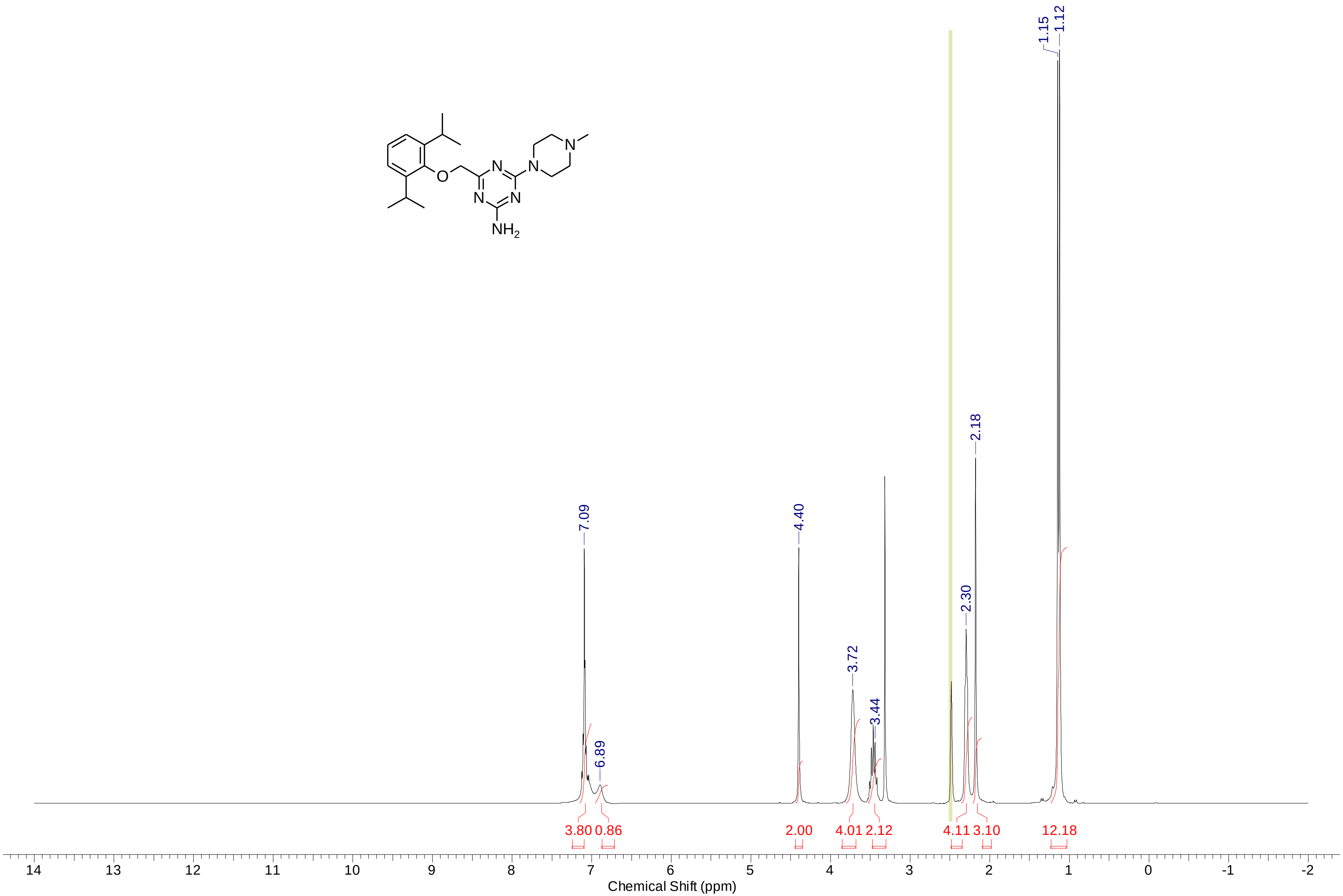

^{1}H NMR spectrum of 4-((2,6-diisopropylphenoxy)methyl)-6-(4-methylpiperazin-1-yl)-1,3,5-triazin-2-amine (**11**)

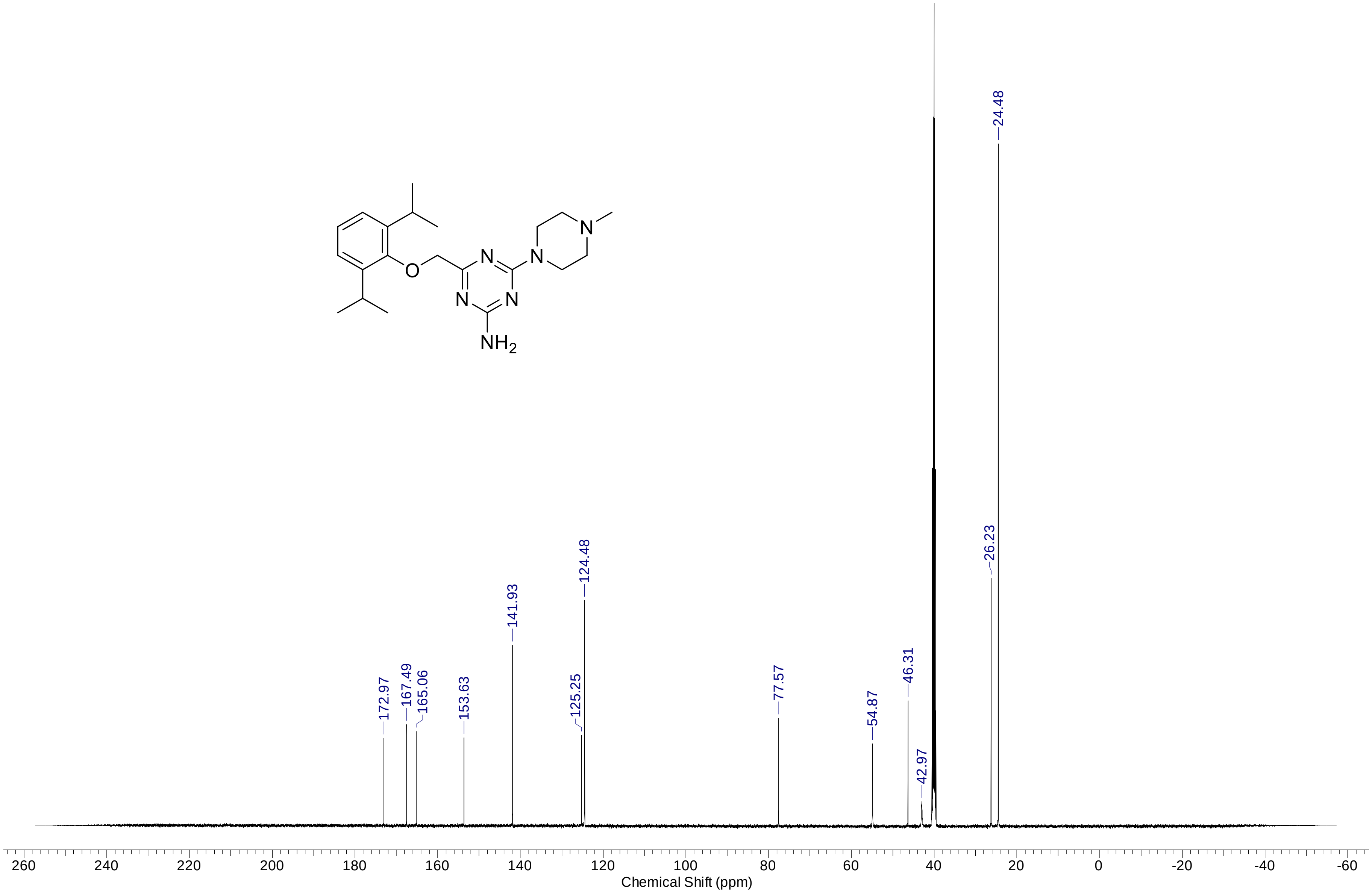

^{13}C NMR spectrum of 4-((2,6-diisopropylphenoxy)methyl)-6-(4-methylpiperazin-1-yl)-1,3,5-triazin-2-amine (**11**)

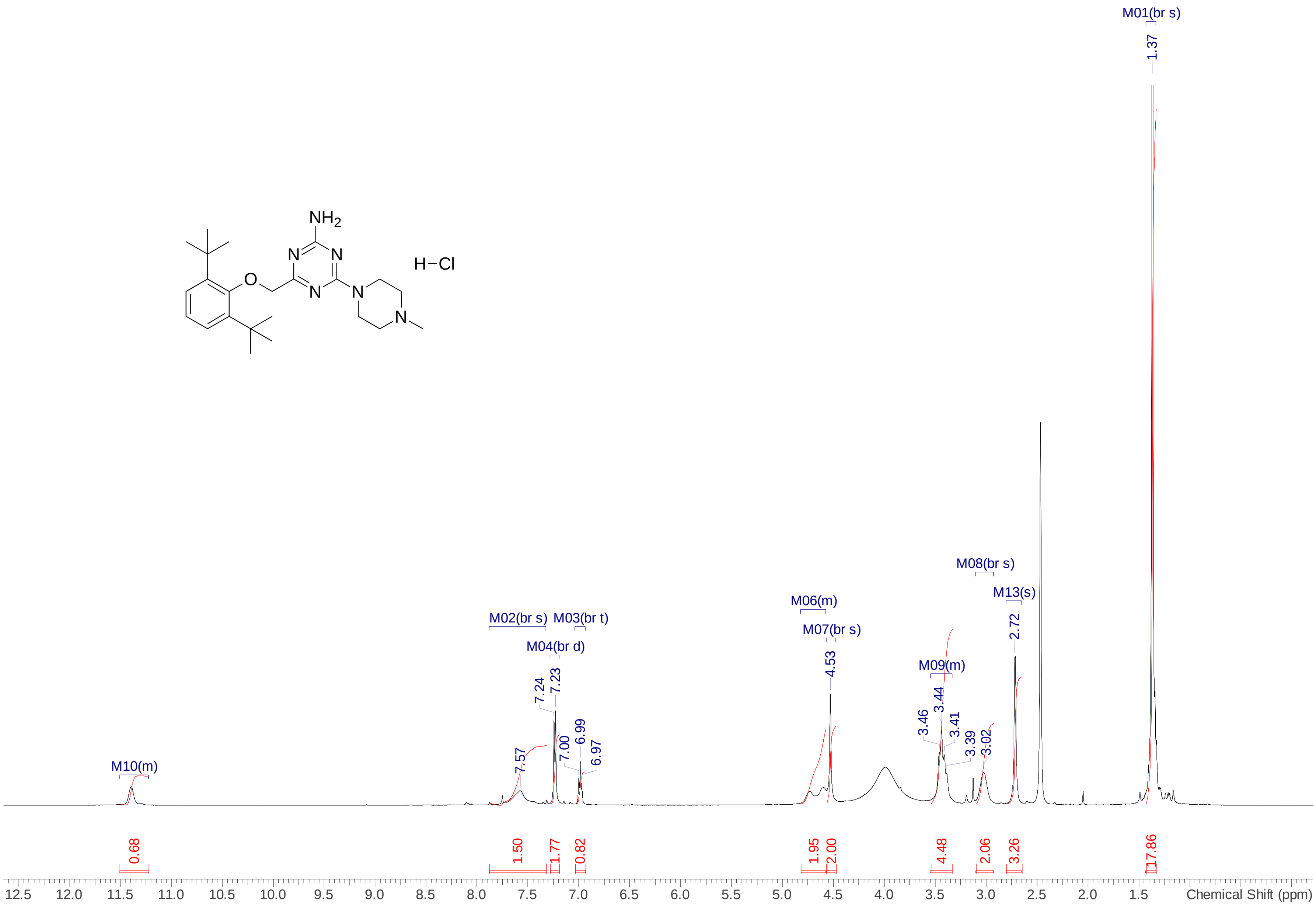

^{1}H NMR spectrum of 4-((2,6-di-*tert*-butylphenoxy)methyl)-6-(4-methylpiperazin-1-yl)-1,3,5-triazin-2-amine hydrochloride (**12**)

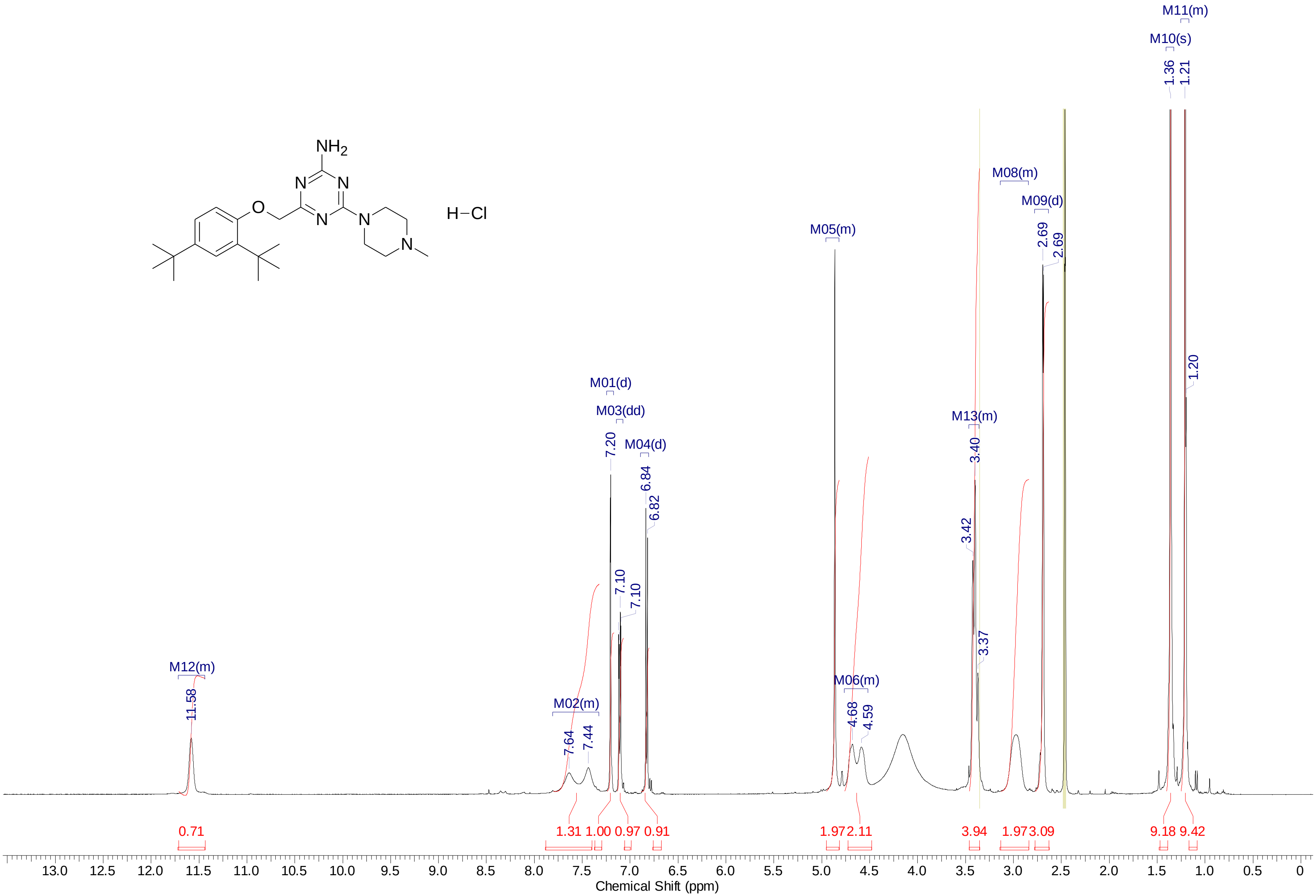

^{1}H NMR spectrum of 4-((2,4-di-*tert*-butylphenoxy)methyl)-6-(4-methylpiperazin-1-yl)-1,3,5-triazin-2-amine hydrochloride (**13**)

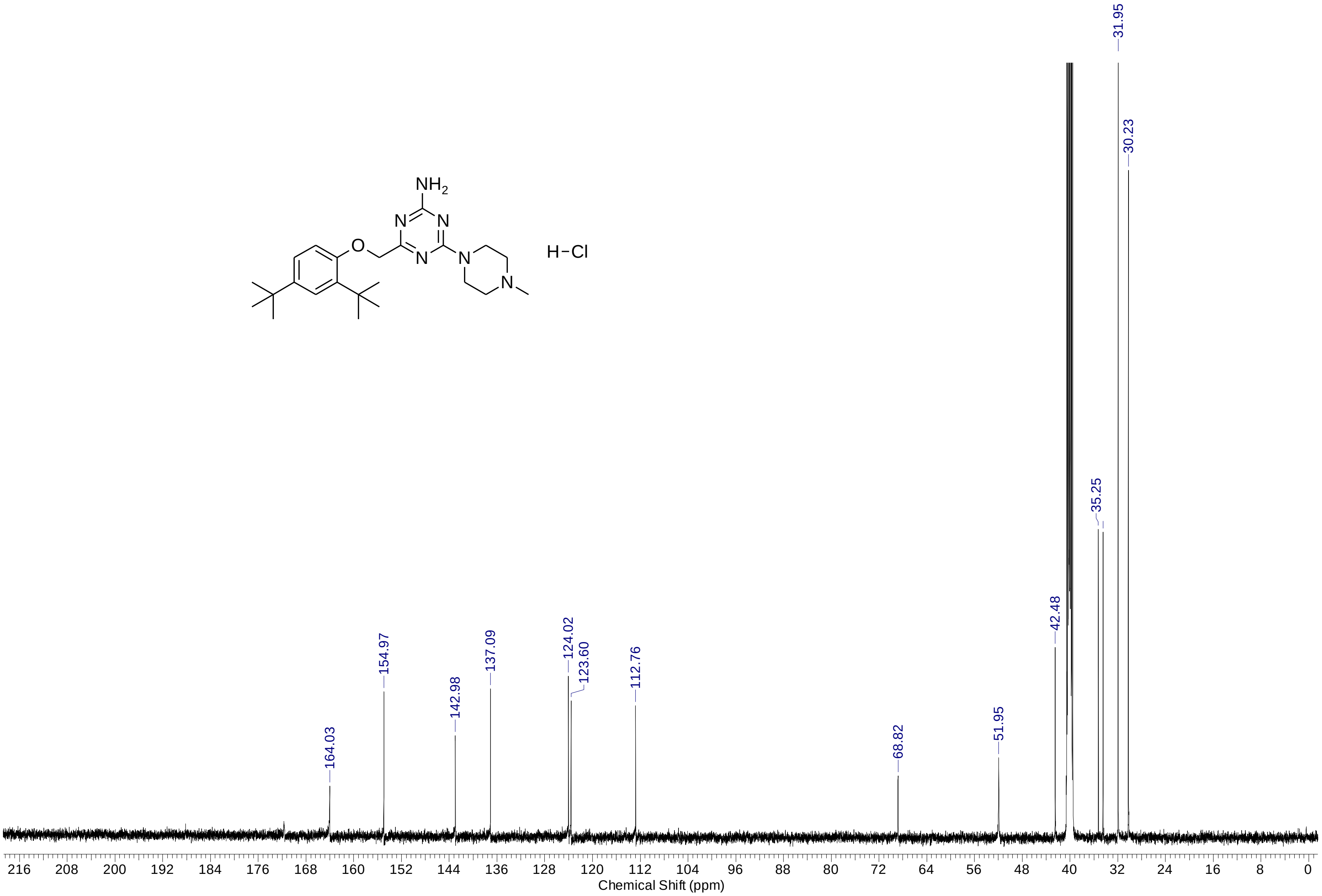

^{13}C NMR spectrum of 4-((2,4-di-*tert*-butylphenoxy)methyl)-6-(4-methylpiperazin-1-yl)-1,3,5-triazin-2-amine hydrochloride (**13**)

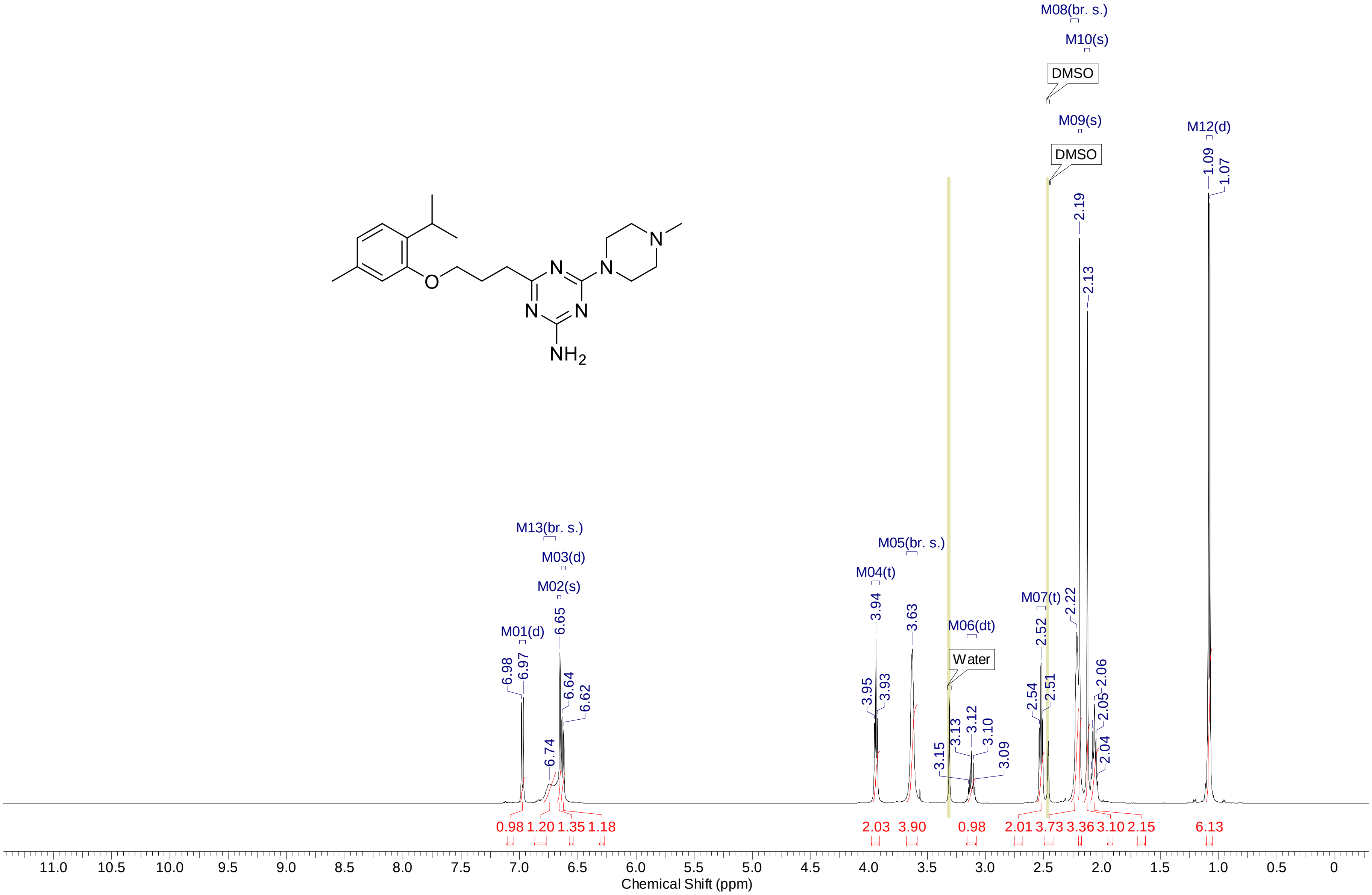

^{1}H NMR spectrum of 4-(3-(2-isopropyl-5-methylphenoxy)propyl)-6-(4-methylpiperazin-1-yl)-1,3,5-triazin-2-amine (**14**)

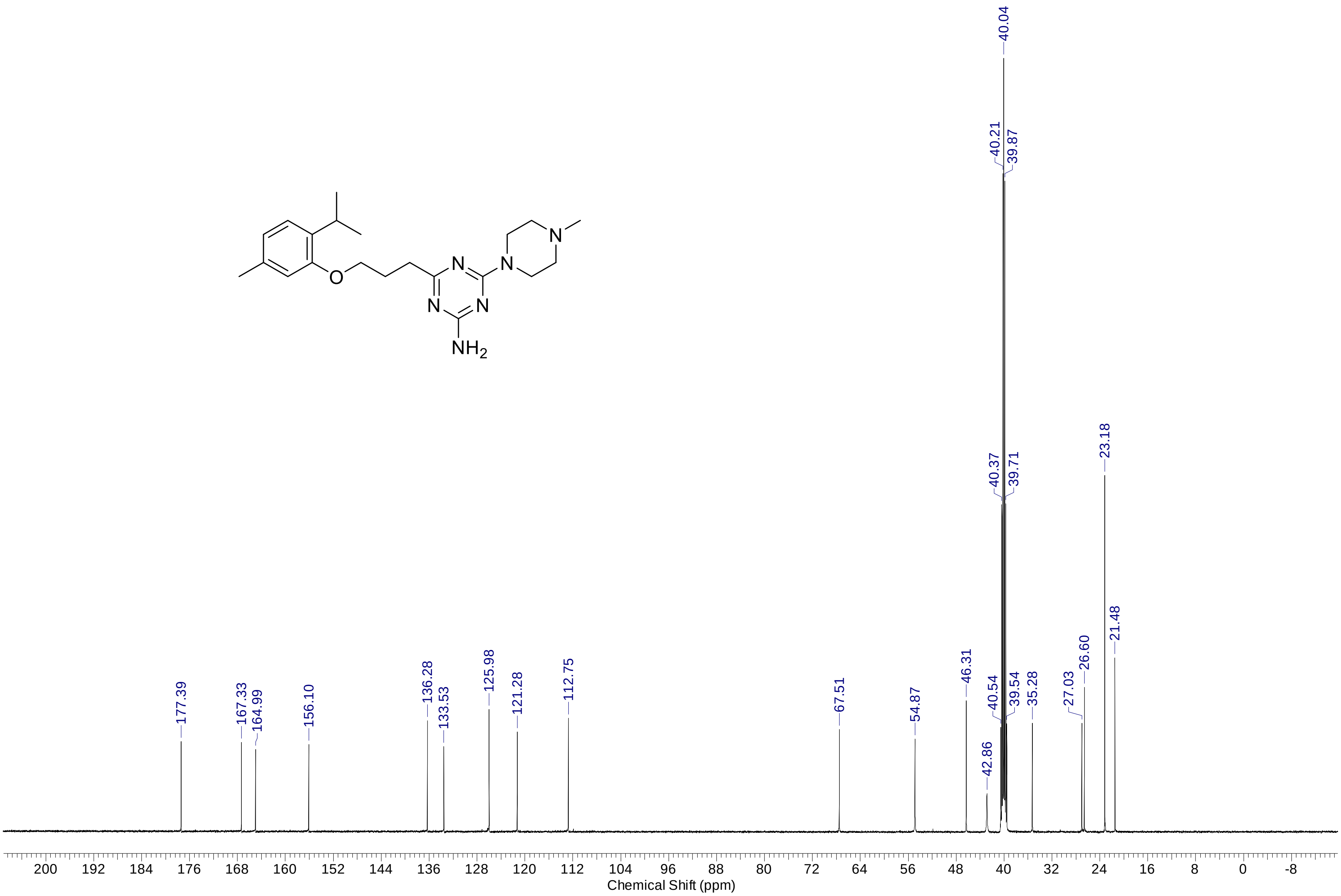

^{13}C NMR spectrum of 4-(3-(2-isopropyl-5-methylphenoxy)propyl)-6-(4-methylpiperazin-1-yl)-1,3,5-triazin-2-amine (**14**)

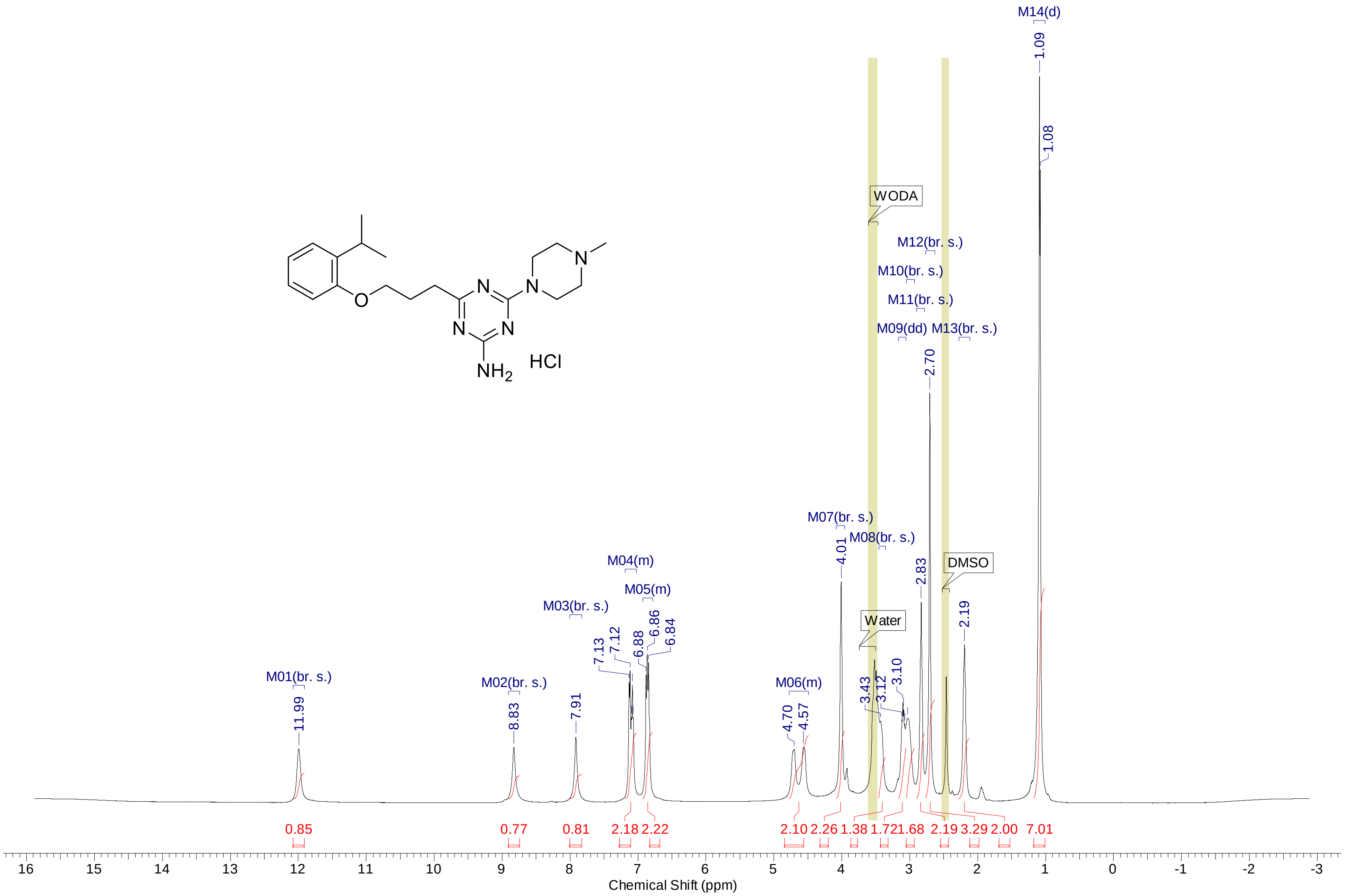

^{1}H NMR spectrum of 4-(3-(2-isopropylphenoxy)propyl)-6-(4-methylpiperazin-1-yl)-1,3,5-triazin-2-amine hydrochloride (**15**)

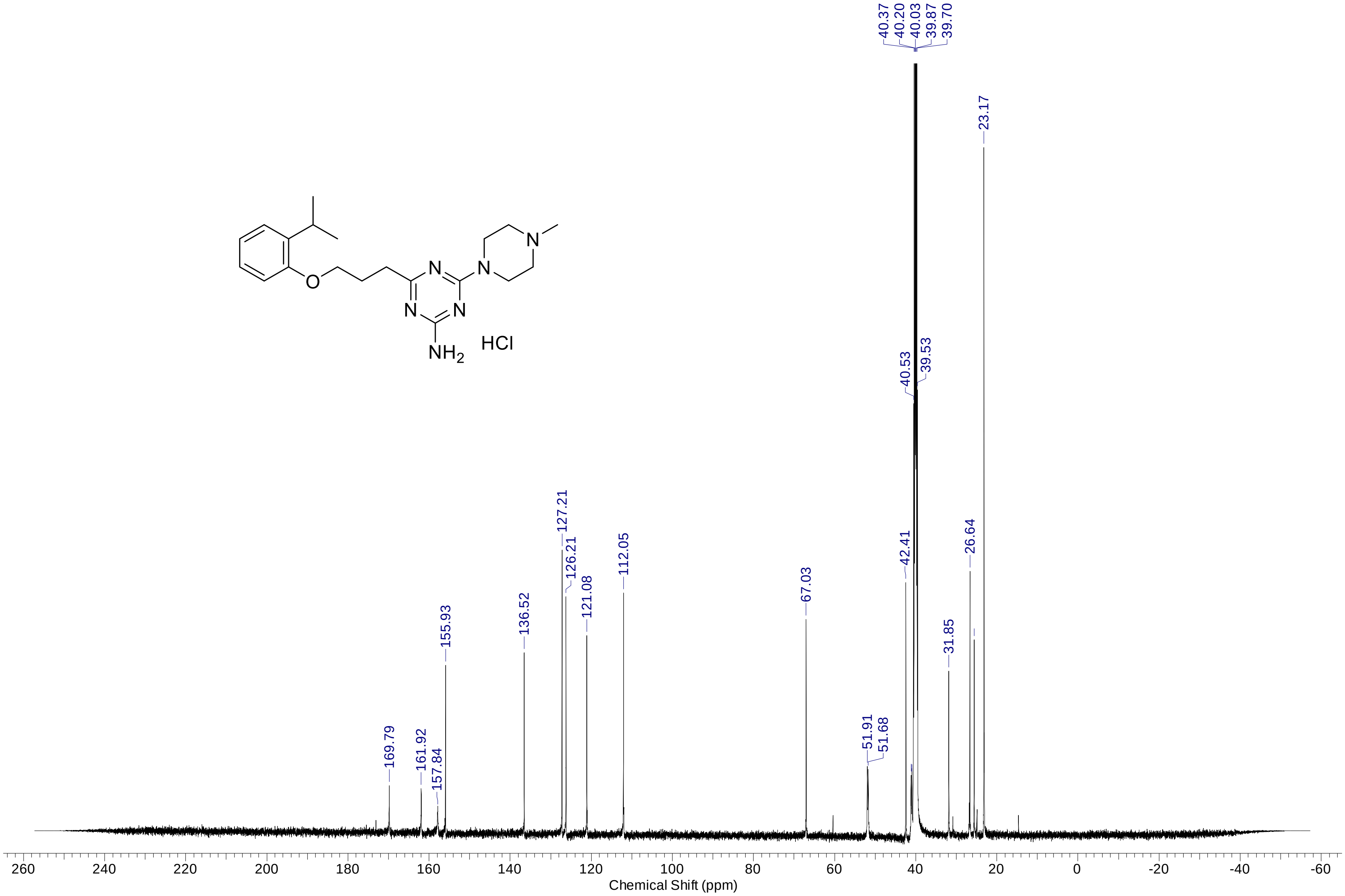

^{13}C NMR spectrum of 4-(3-(2-isopropylphenoxy)propyl)-6-(4-methylpiperazin-1-yl)-1,3,5-triazin-2-amine hydrochloride (**15**)

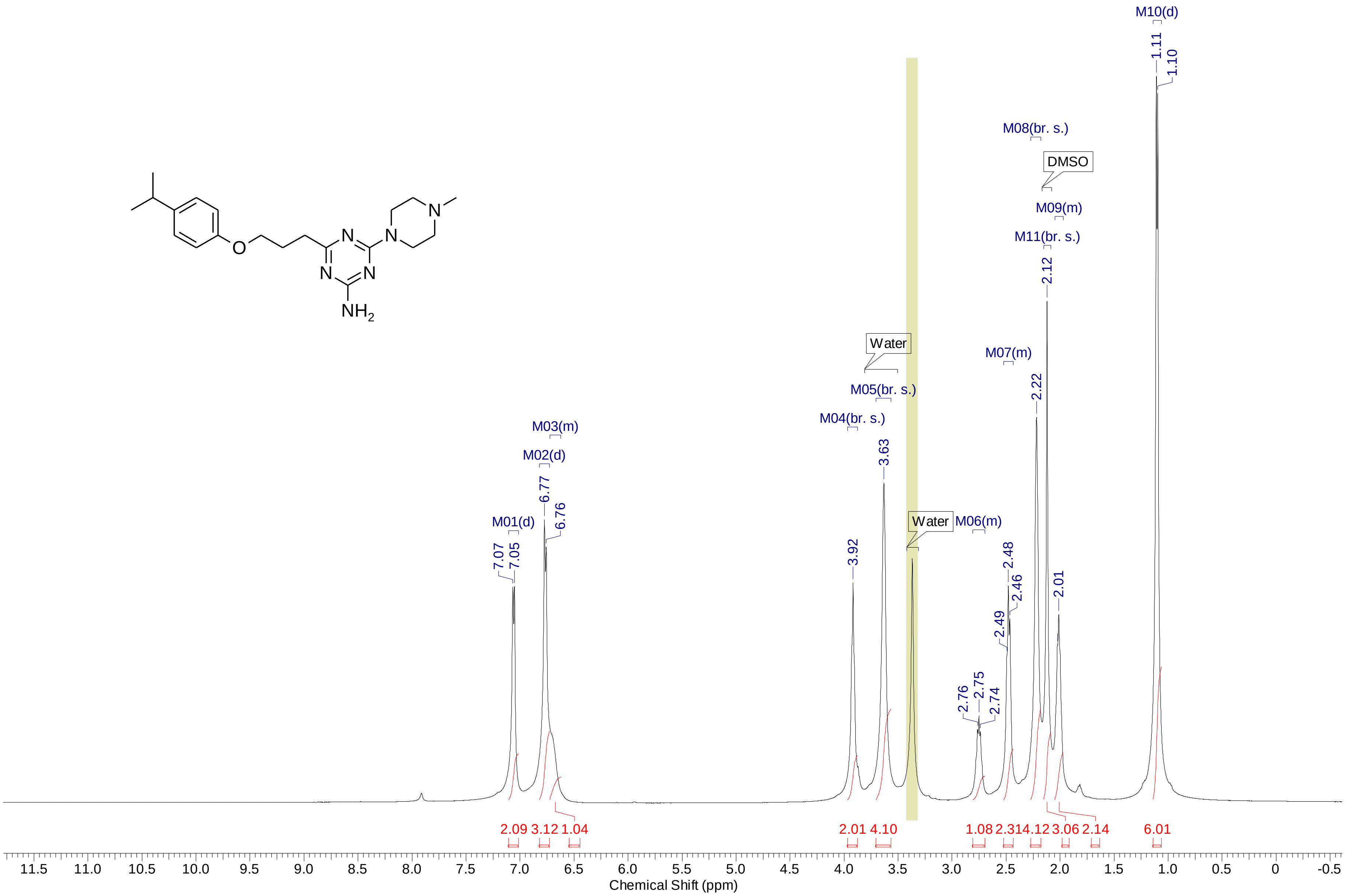

^{1}H NMR spectrum of 4-(3-(4-isopropylphenoxy)propyl)-6-(4-methylpiperazin-1-yl)-1,3,5-triazin-2-amine (**16**)

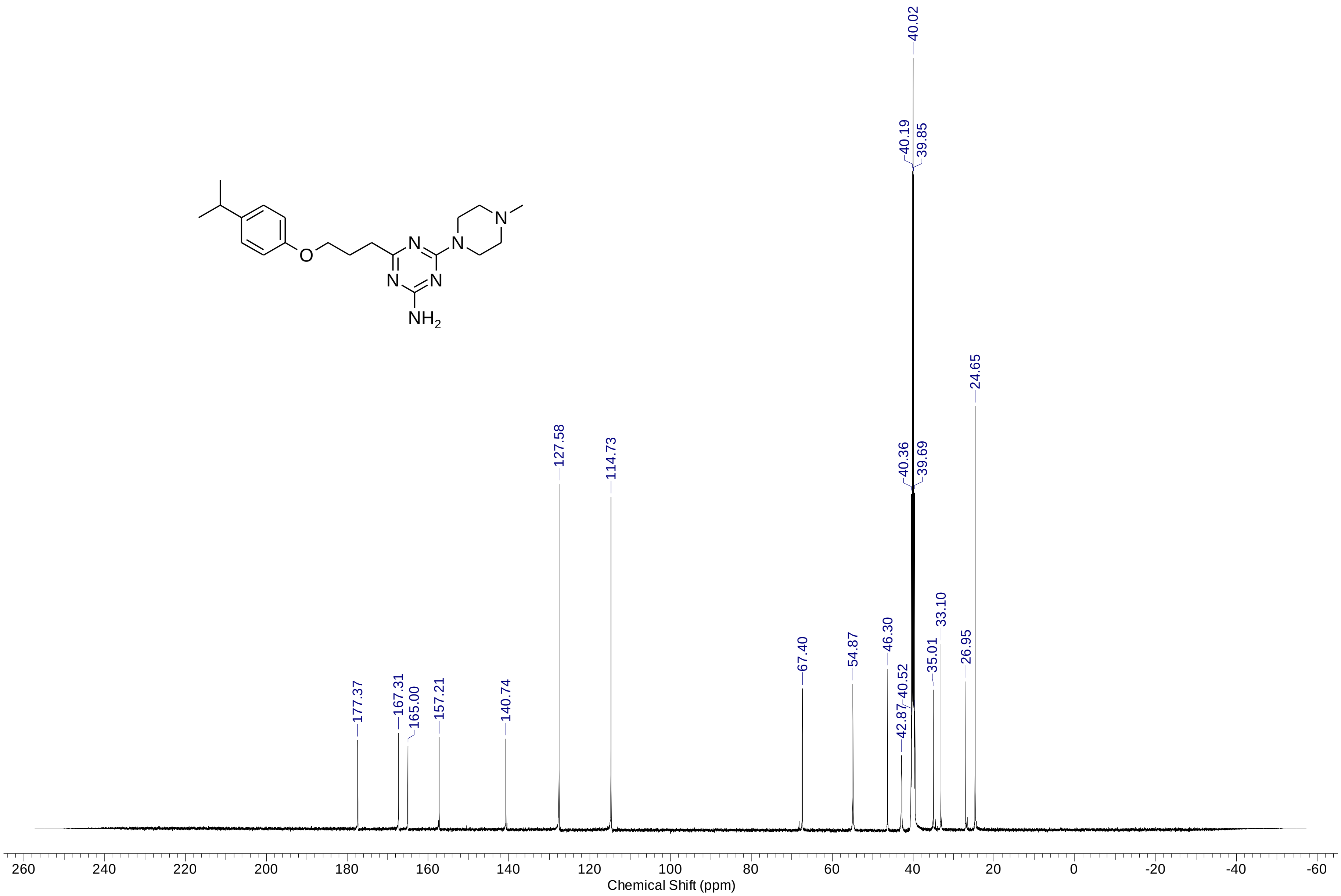

^{13}C NMR spectrum of 4-(3-(4-isopropylphenoxy)propyl)-6-(4-methylpiperazin-1-yl)-1,3,5-triazin-2-amine (**16**)

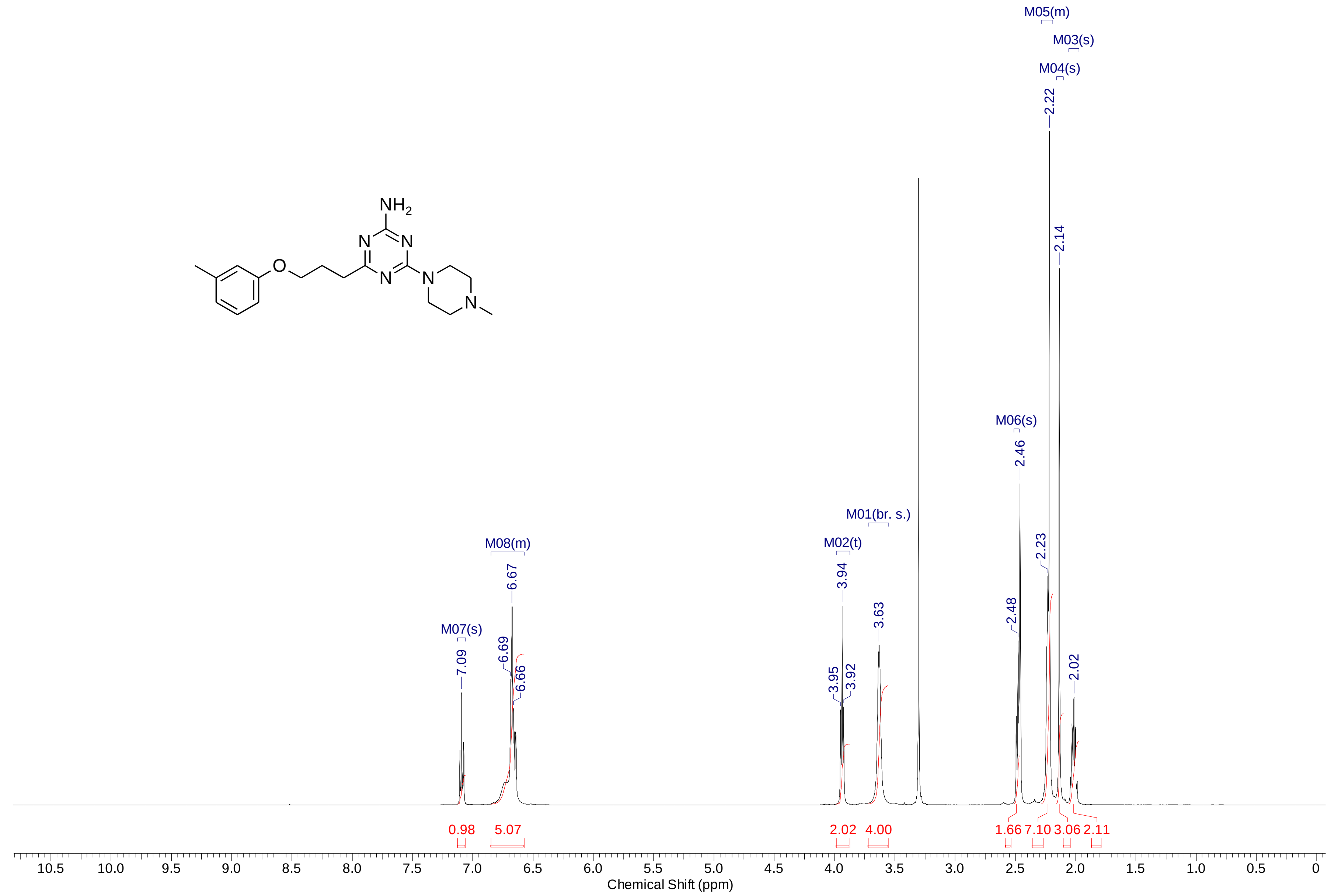

^{1}H NMR spectrum of 4-(4-methylpiperazin-1-yl)-6-(3-(*m*-tolyloxy)propyl)-1,3,5-triazin-2-amine (**17**)

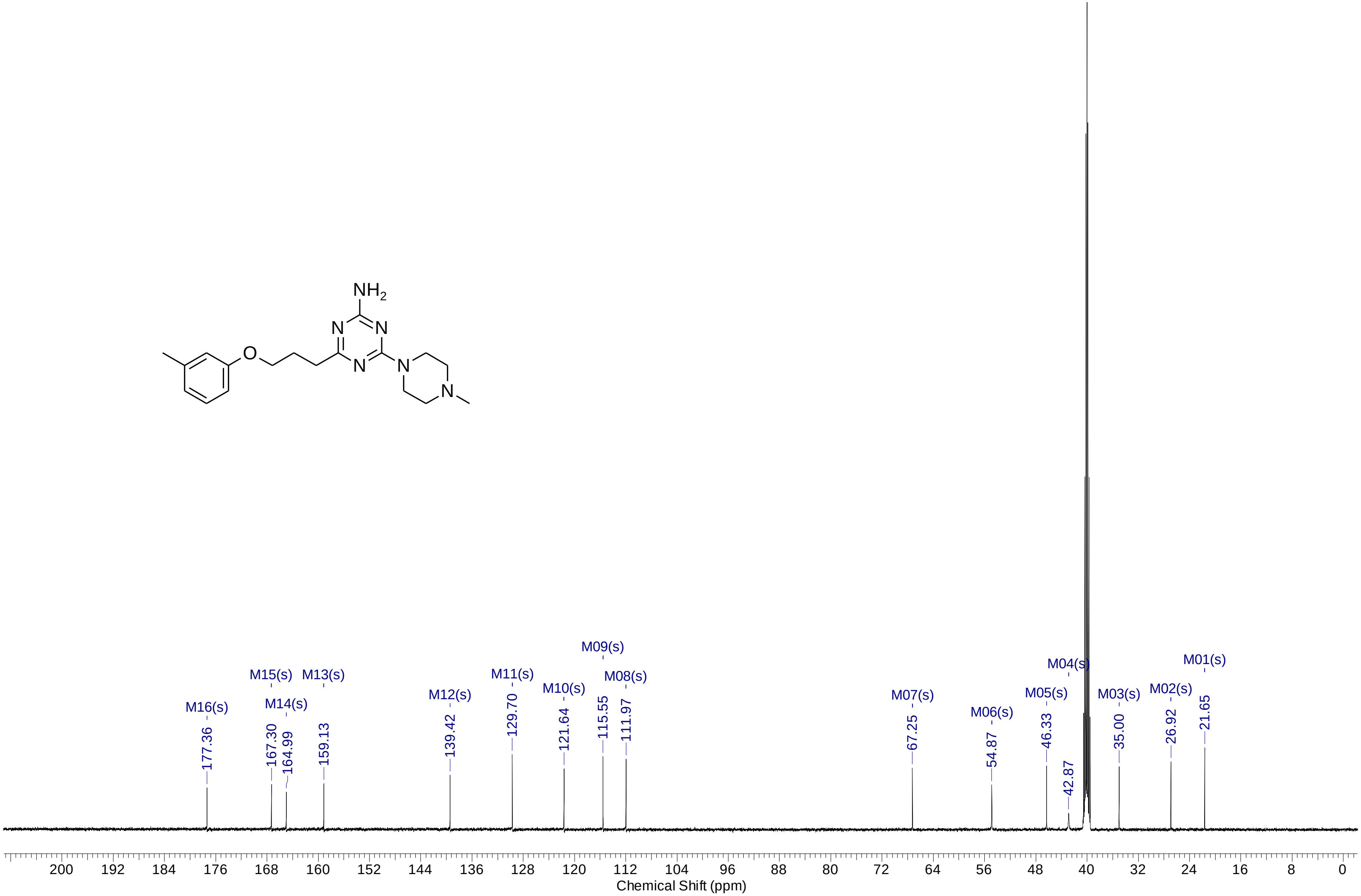

^{13}C NMR spectrum of 4-(4-methylpiperazin-1-yl)-6-(3-(*m*-tolyloxy)propyl)-1,3,5-triazin-2-amine (**17**)

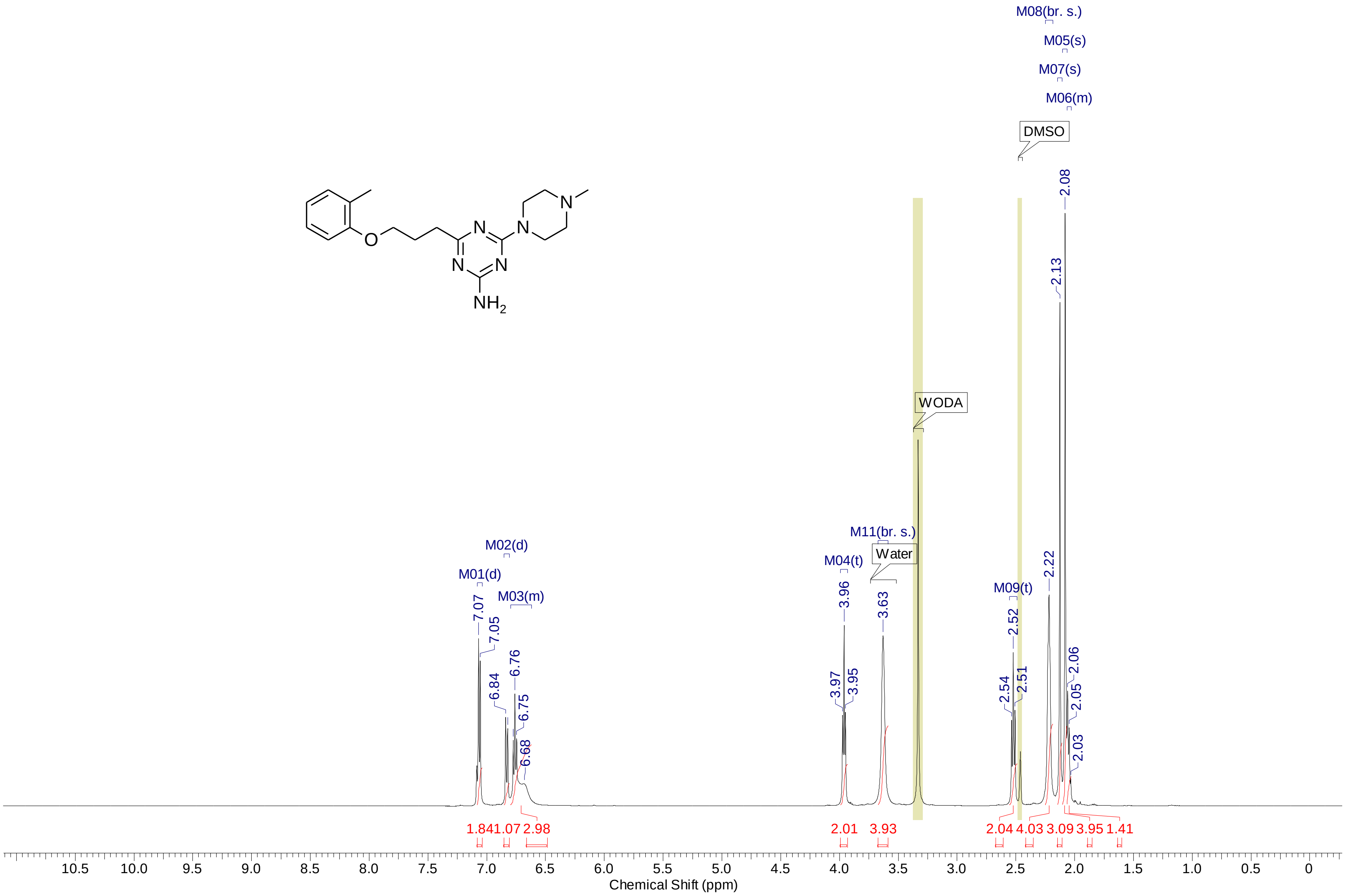

^{1}H NMR spectrum of 4-(4-methylpiperazin-1-yl)-6-(3-(*o*-tolyloxy)propyl)-1,3,5-triazin-2-amine (**18**)

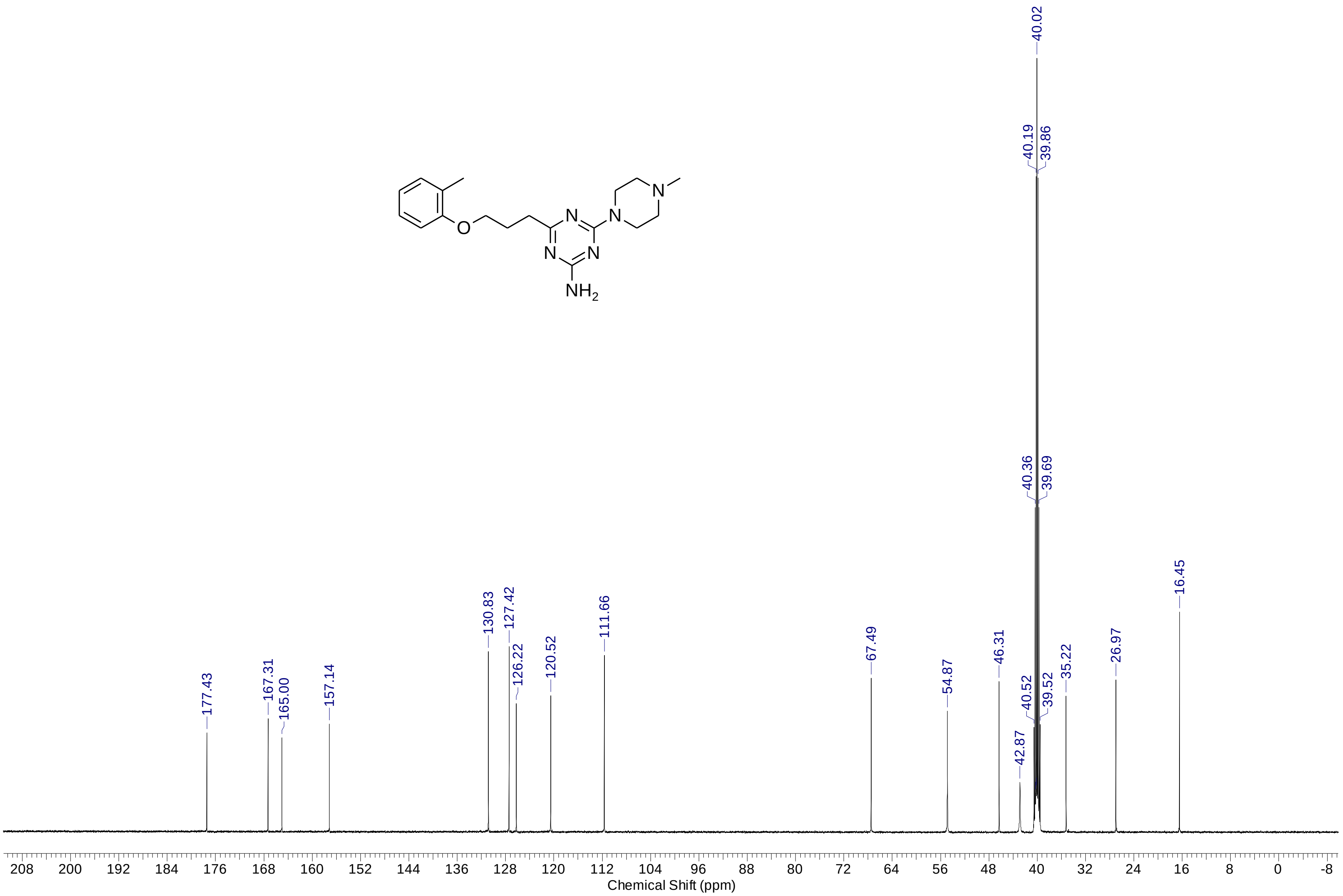

^{13}C NMR spectrum of 4-(4-methylpiperazin-1-yl)-6-(3-*o*-tolyloxy)propyl)-1,3,5-triazin-2-amine (**18**)

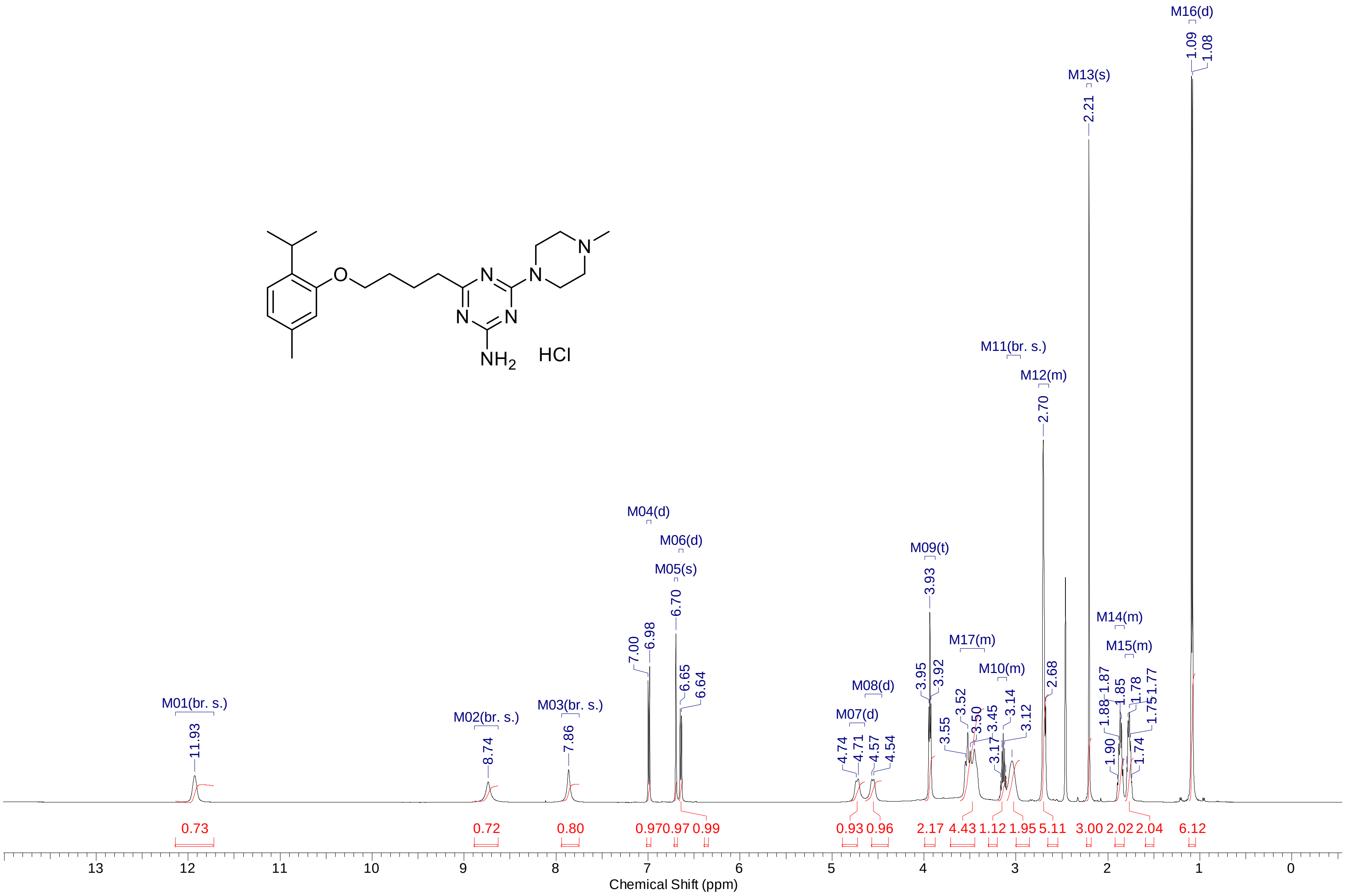

^{1}H NMR spectrum of 4-(4-(2-isopropyl-5-methylphenoxy)butyl)-6-(4-methylpiperazin-1-yl)-1,3,5-triazin-2-amine hydrochloride (**19**)

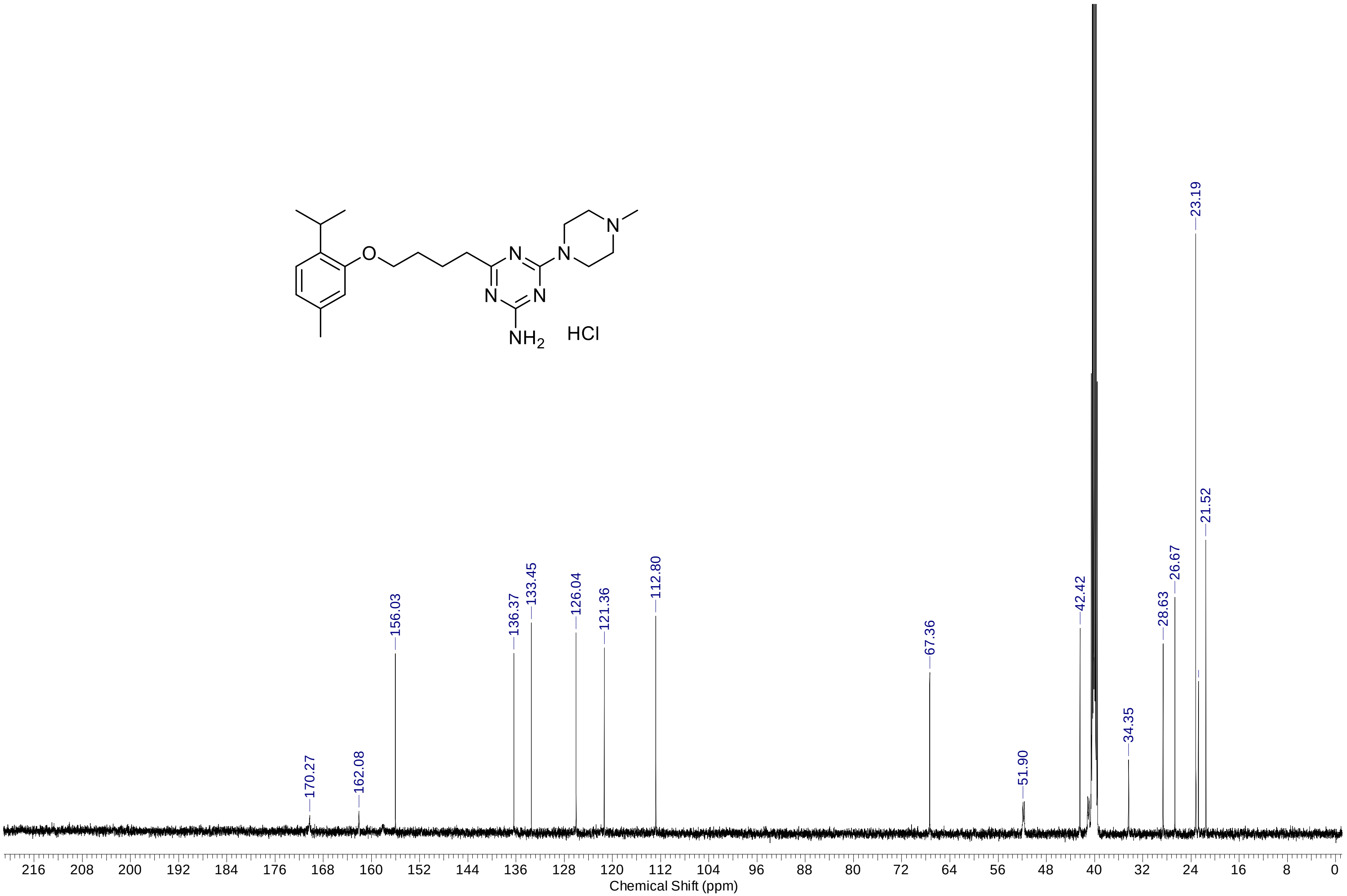

^{13}C NMR spectrum of 4-(4-(2-isopropyl-5-methylphenoxy)butyl)-6-(4-methylpiperazin-1-yl)-1,3,5-triazin-2-amine hydrochloride (**19**)

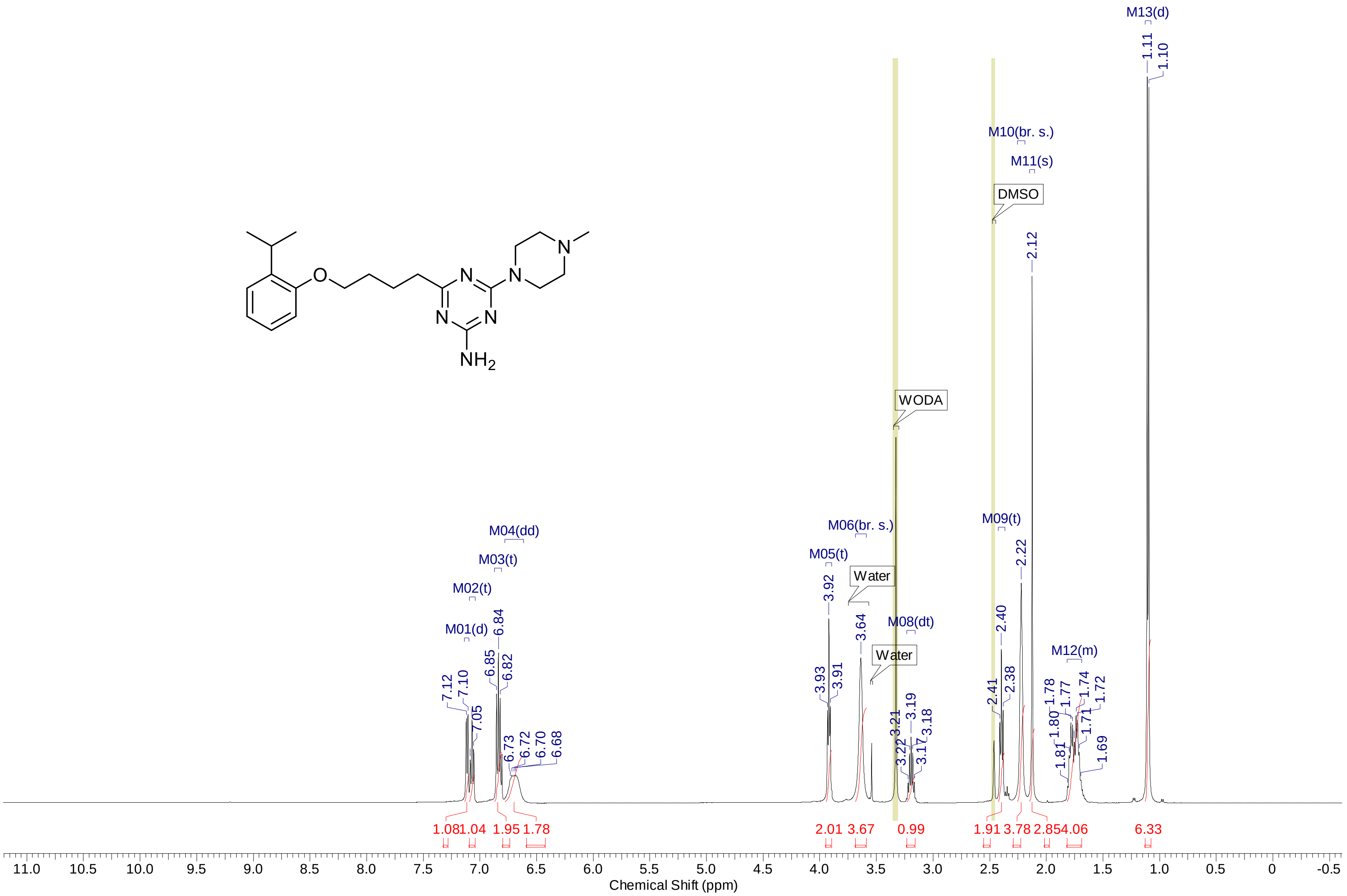

^{1}H NMR spectrum of 4-(4-(2-isopropylphenoxy)butyl)-6-(4-methylpiperazin-1-yl)-1,3,5-triazin-2-amine (**20**)

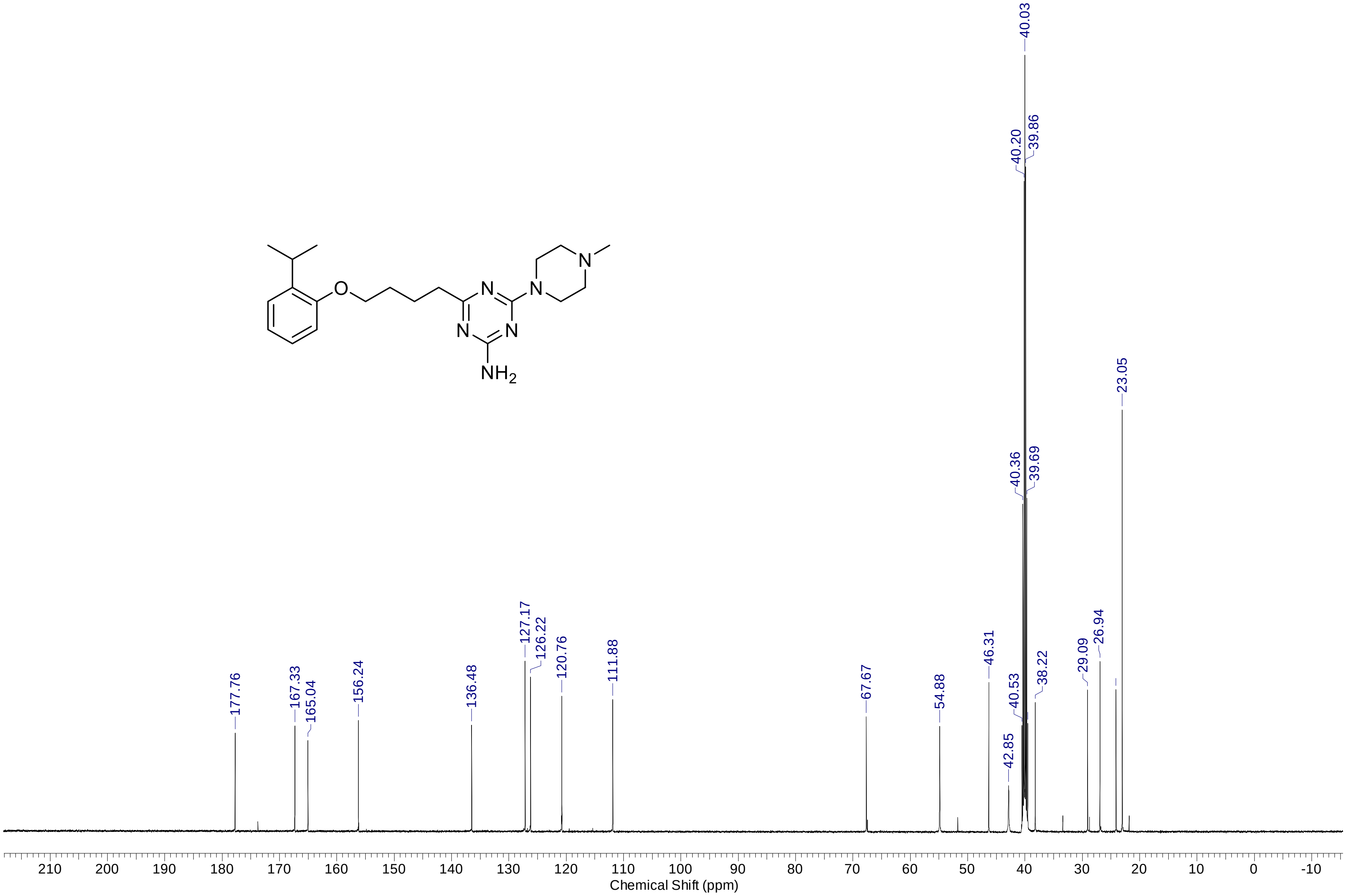

^{13}C NMR spectrum of 4-(4-(2-isopropylphenoxy)butyl)-6-(4-methylpiperazin-1-yl)-1,3,5-triazin-2-amine (**20**)

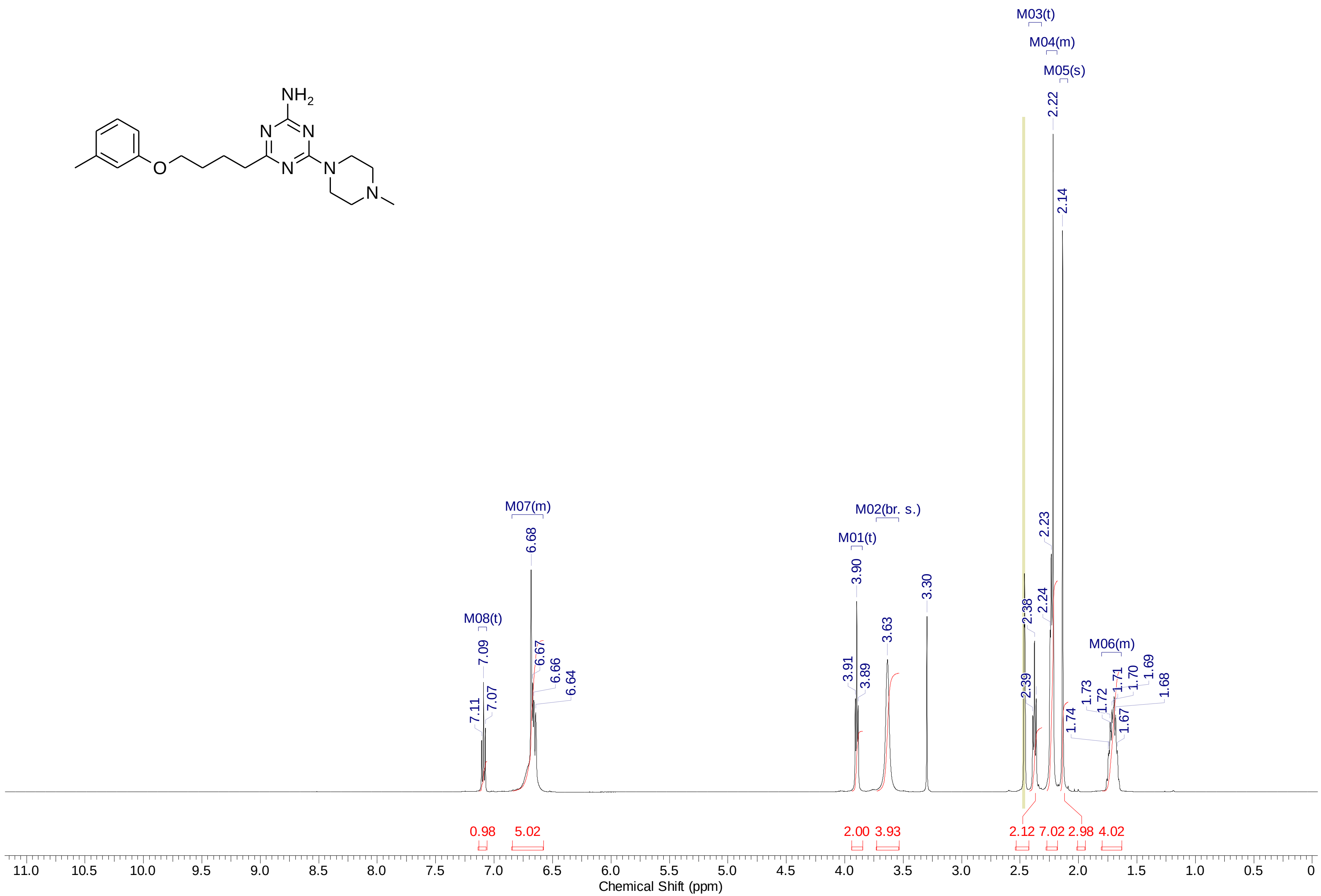

^{1}H NMR spectrum of 4-(4-methylpiperazin-1-yl)-6-(4-(*m*-tolyloxy)butyl)-1,3,5-triazin-2-amine (**21**)

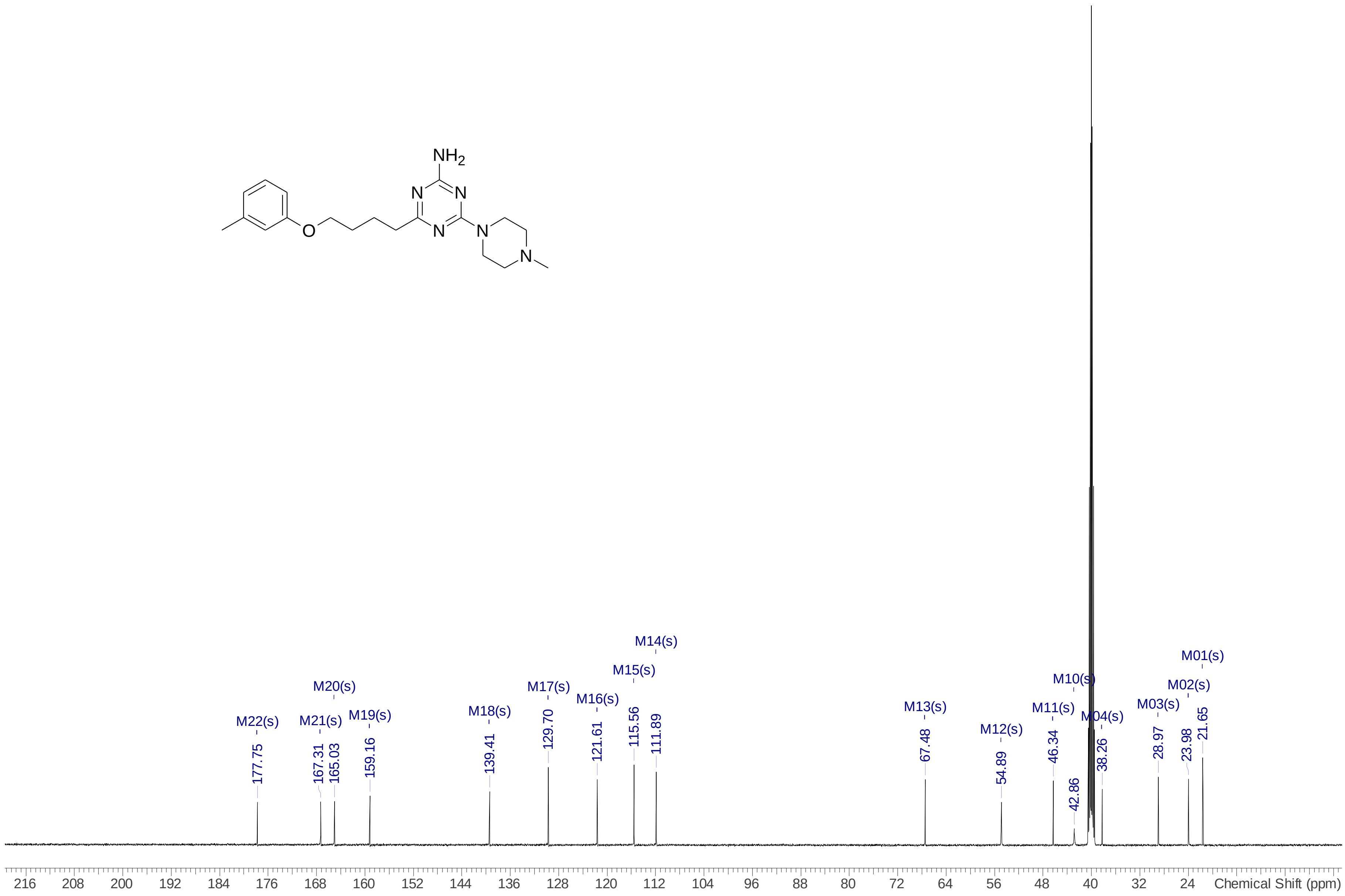

^{13}C NMR spectrum of 4-(4-methylpiperazin-1-yl)-6-(4-(*m*-tolyloxy)butyl)-1,3,5-triazin-2-amine (**21**)

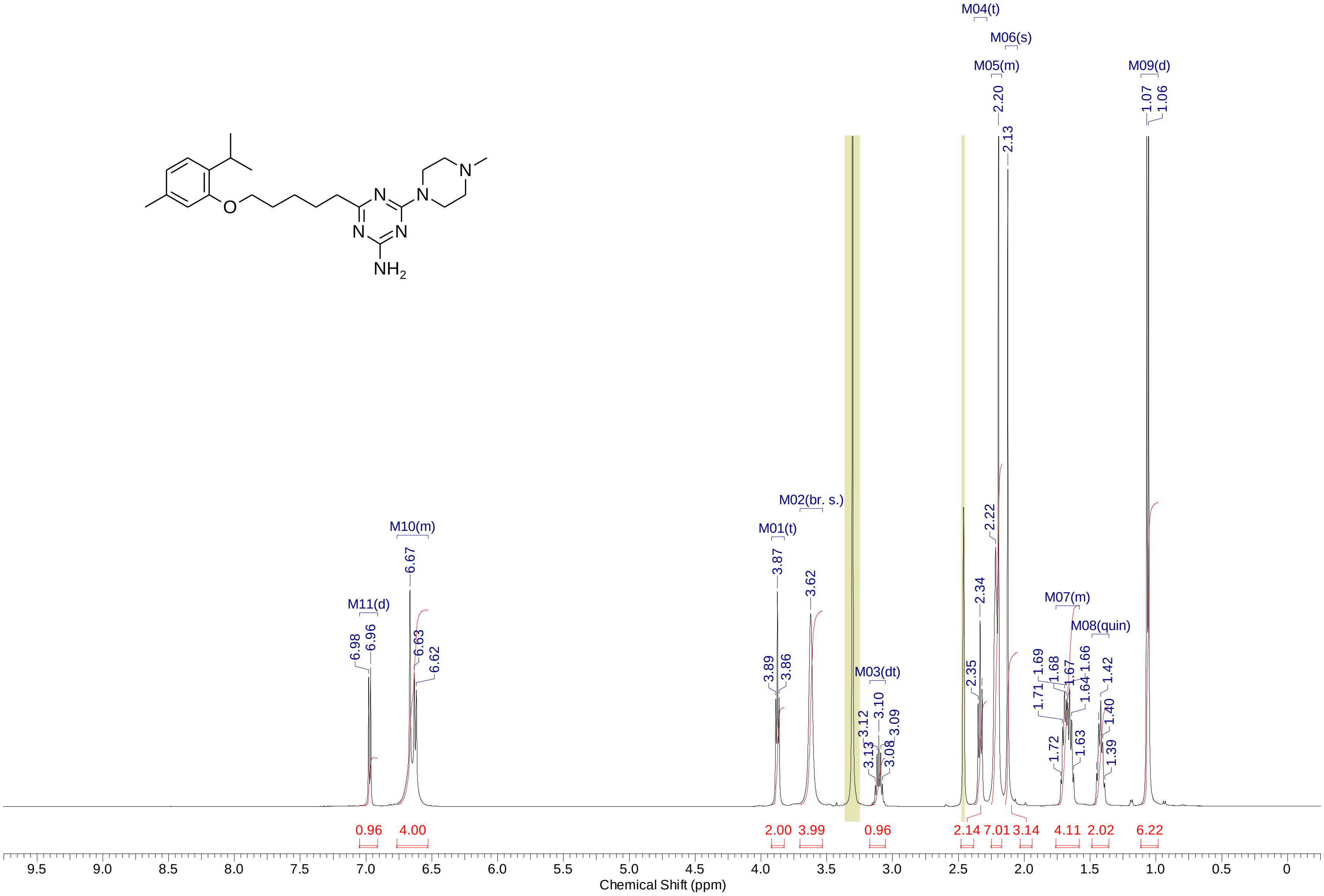

^{1}H NMR spectrum of 4-(5-(2-isopropyl-5-methylphenoxy)pentyl)-6-(4-methylpiperazin-1-yl)-1,3,5-triazin-2-amine (**22**)

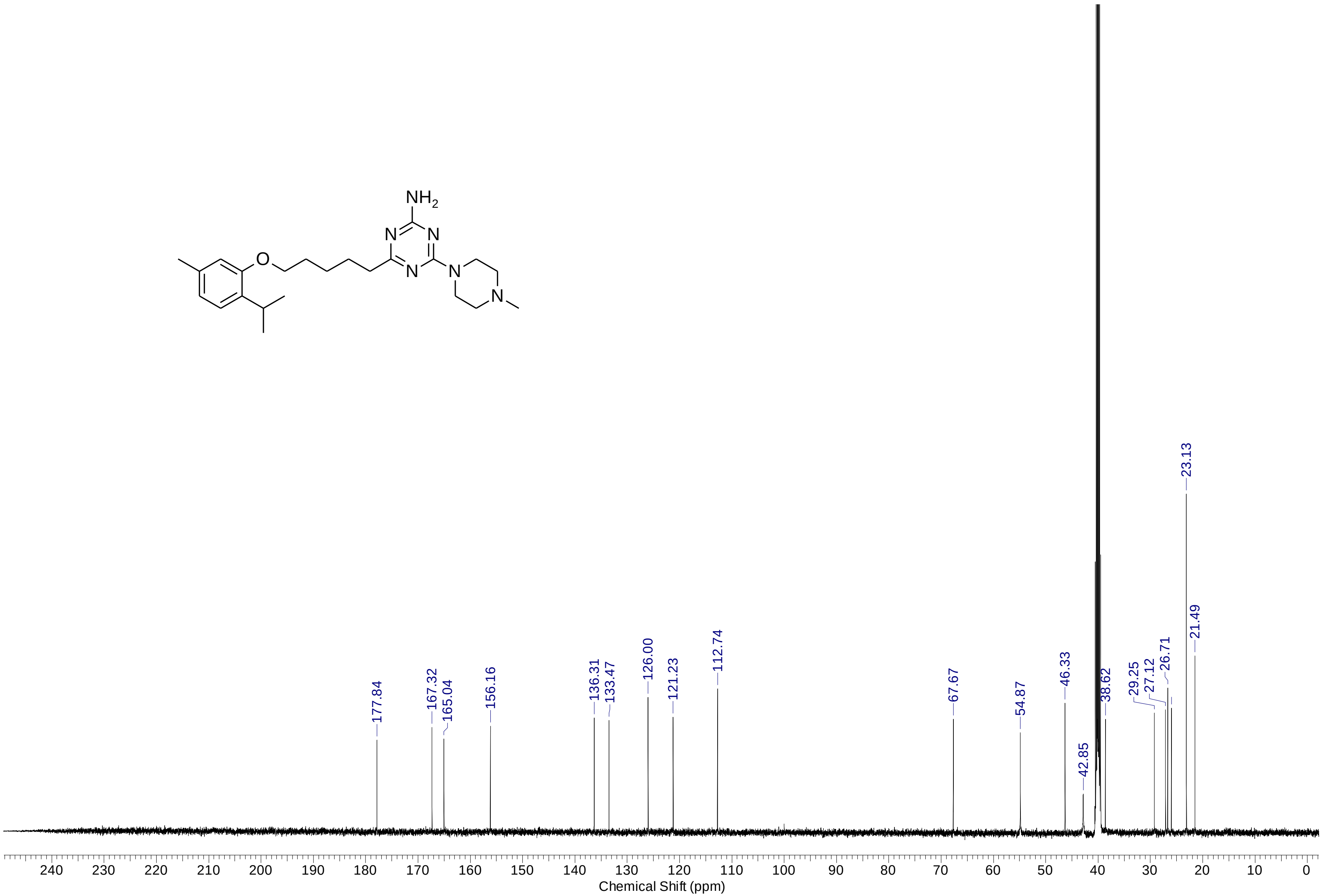

^{13}C NMR spectrum of 4-(5-(2-isopropyl-5-methylphenoxy)pentyl)-6-(4-methylpiperazin-1-yl)-1,3,5-triazin-2-amine (**22**)

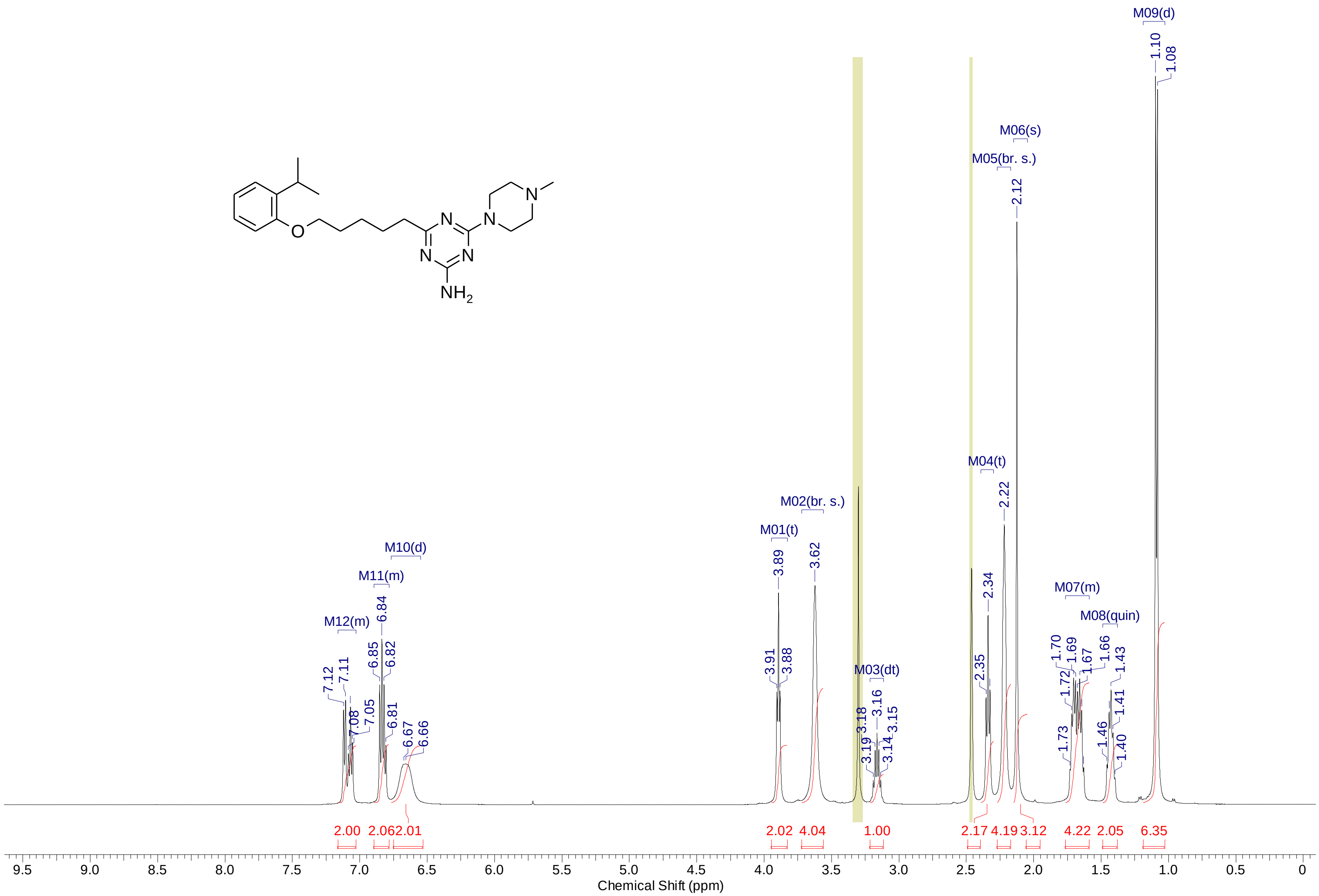

^{1}H NMR spectrum of 4-(5-(2-isopropylphenoxy)pentyl)-6-(4-methylpiperazin-1-yl)-1,3,5-triazin-2-amine (**23**)

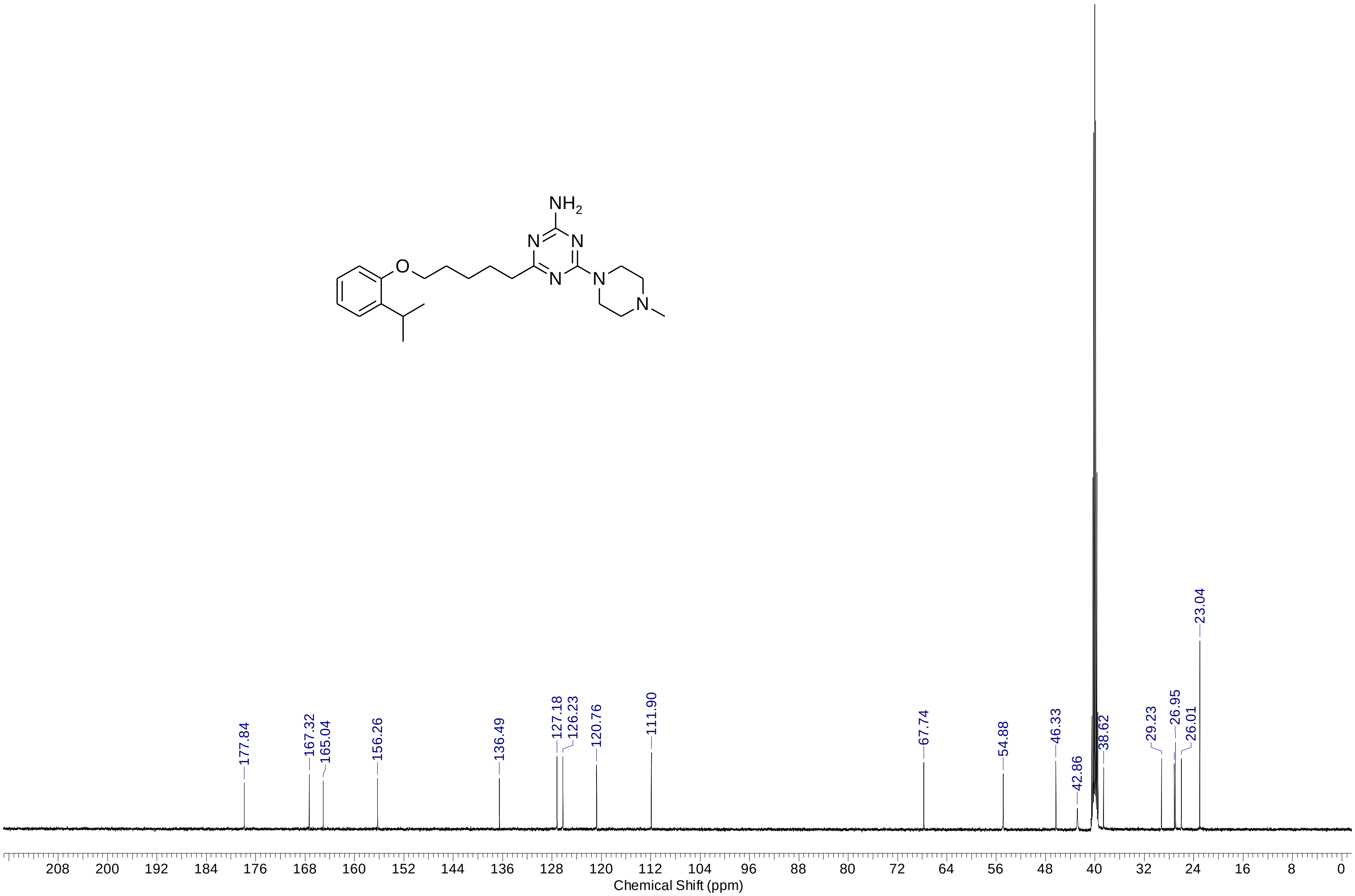

^{13}C NMR spectrum of 4-(5-(2-isopropylphenoxy)pentyl)-6-(4-methylpiperazin-1-yl)-1,3,5-triazin-2-amine (**23**)

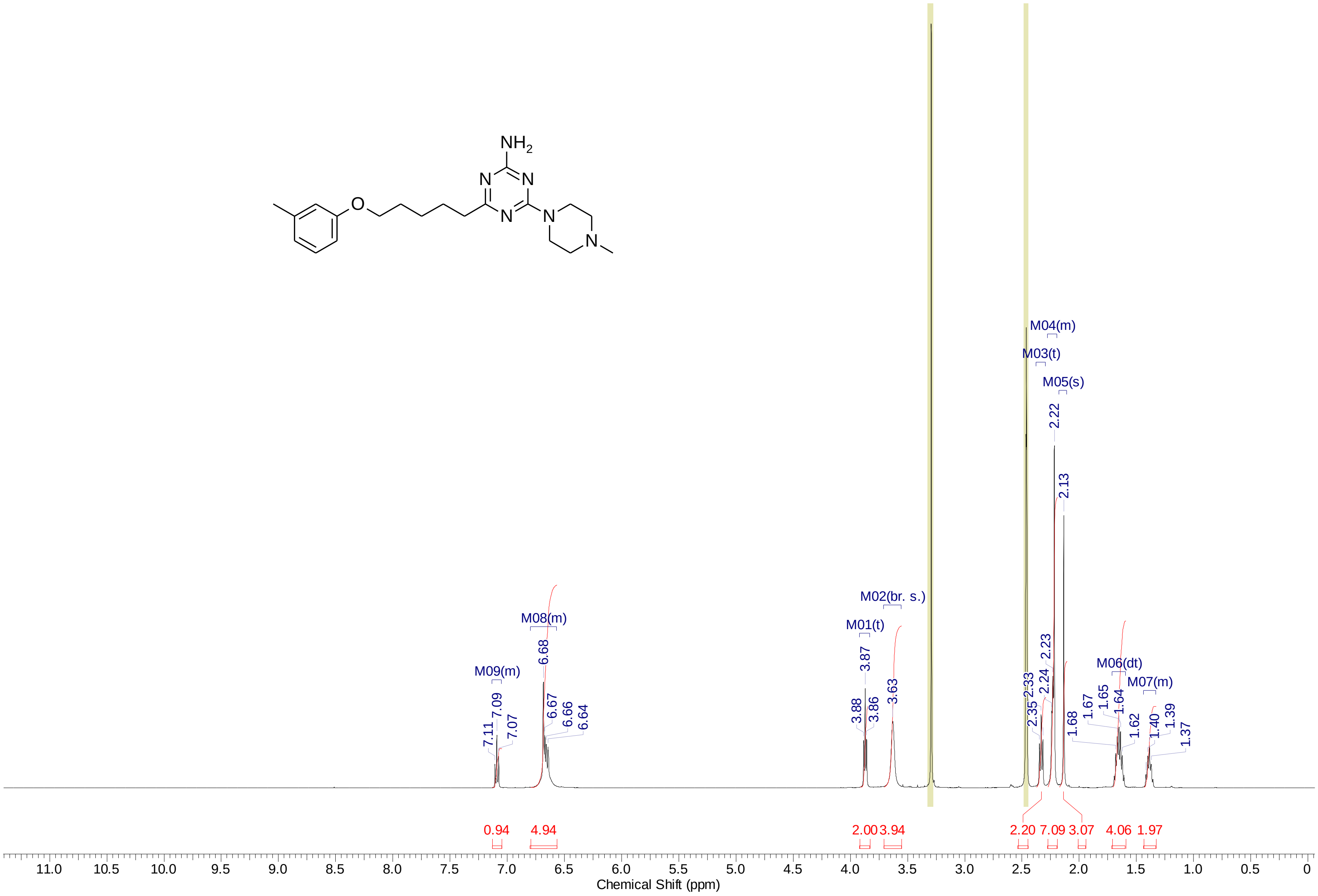

^{1}H NMR spectrum of 4-(4-methylpiperazin-1-yl)-6-(5-(*m*-tolyloxy)pentyl) 1,3,5-triazin-2-amine (**24**)

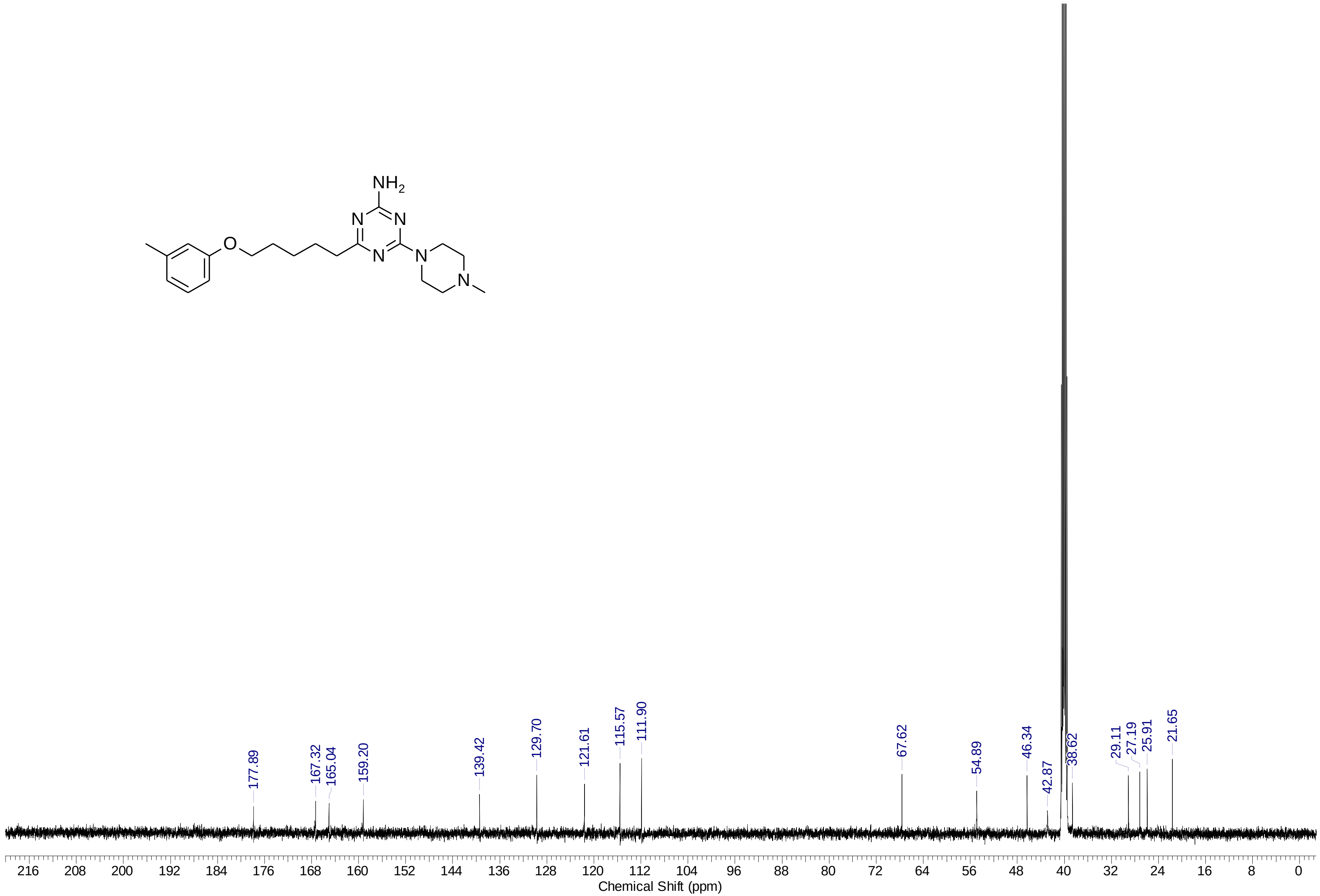

^{13}C NMR spectrum of 4-(4-methylpiperazin-1-yl)-6-(5-(*m*-tolyloxy)pentyl) 1,3,5-triazin-2-amine (**24**)

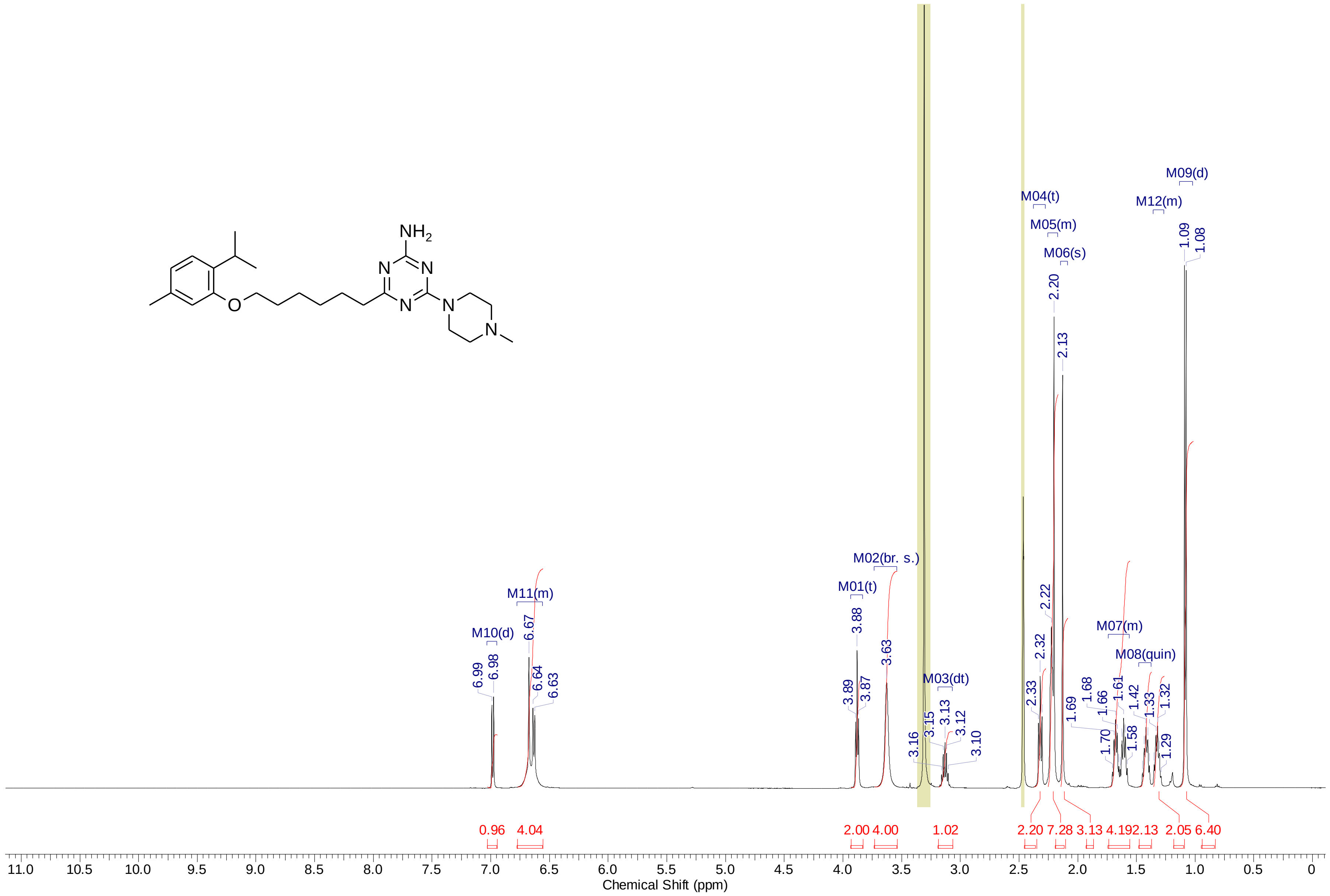

^{1}H NMR spectrum of 4-(6-(2-isopropyl-5-methylphenoxy)hexyl)-6-(4-methylpiperazin-1-yl)-1,3,5-triazin-2-amine (**25**)

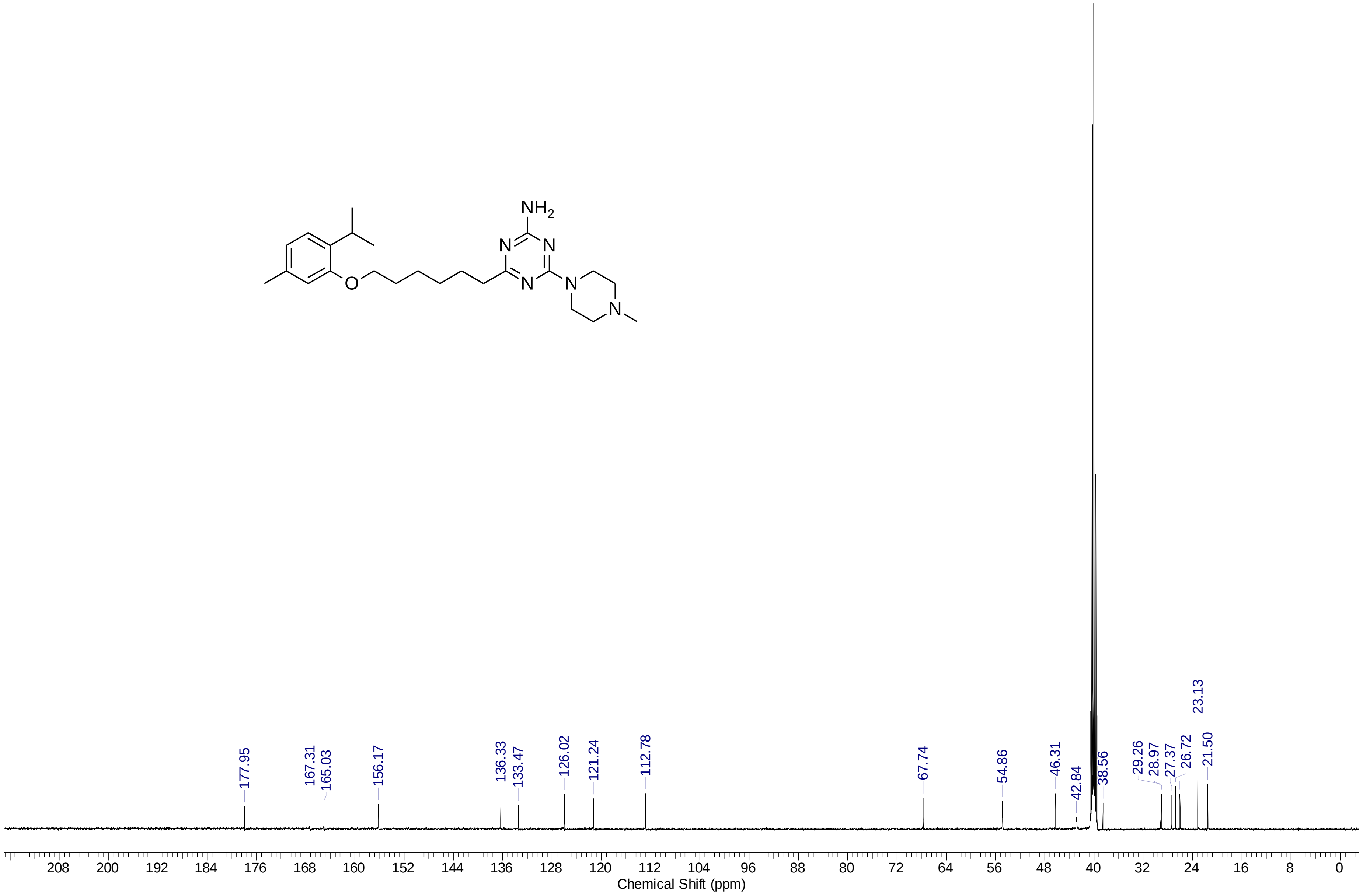

^{13}C NMR spectrum of 4-(6-(2-isopropyl-5-methylphenoxy)hexyl)-6-(4-methylpiperazin-1-yl)-1,3,5-triazin-2-amine (**25**)
