# Supplementary material for: New Triazine Derivatives as Serotonin 5-HT6 Receptor Ligands
Source: Molecules. 2023 Jan 22;28(3):1108. doi: 10.3390/molecules28031108 (PMC9919591; doi:10.3390/molecules28031108)
Supplement: Supplementary file 1 [file molecules-28-01108-s001.zip › Supplementary Materials S1.pdf]

Supplementary S1

New triazine derivatives as serotonin 5-HT$_6$ receptor ligands

Dorota Łażewska[1]*, Małgorzata Więcek[1], Grzegorz Satała[2], Paulina Chałupnik[1], Ewa Żesławska[3], Ewelina Honkisz-Orzechowska[1], Monika Tarasek[1], Gniewomir Latacz[1], Wojciech Nitek[4], Ewa Szymańska[1] and Jadwiga Handzlik[1]*

[1]Department of Technology and Biotechnology of Drugs, Faculty of Pharmacy, Jagiellonian University Medical College in Kraków, Medyczna 9, 30-688 Krakow, Poland;
[2]Department of Medicinal Chemistry, Maj Institute of Pharmacology, Polish Academy of Sciences, Smętna 12, 31-343 Kraków, Poland;
[3] Institute of Biology, Pedagogical University of Kraków, Podchorążych 2, 30-084 Kraków, Poland;
[4]Faculty of Chemistry, Jagiellonian University in Kraków, Gronostajowa 2, 30-387 Kraków, Poland

* Correspondence: dorota.lazewska@uj.edu.pl (D.Ł.); j.handzlik@uj.edu.pl (J.H.)

1. Synthesis of intermediates
2. ^{1}H NMR spectra of selected intermediates (Fig. S1- Fig. S11)
3. Water solubility determination (calibration curves and Marvin prediction of logD and pK$_a$ for compound **3**; Fig. S12-Fig. S16)

1. Synthesis of intermediates:

1.1. 4-Methylpiperazin-1-yl biguanidine dihydrochloride (**TR-1**) was obtained as described previously [1].
1.2. General synthetic procedure for substituted phenoxyalkylic acid esters (**2a-25a**)

Esters were obtained and purified as described previously [2] with minor modifications.
General procedure:
A mixture of substituted phenol (0.01 mol), suitable bromoester (0.012 mol), anhydrous potassium or cesium carbonate (0.02 mol) and acetone or acetonitrile (30 ml) were refluxed for 10–24 h. The reaction mixture was filtered and solvent was removed by distillation. The residual mass was diluted in ethyl acetate or dichloromethane (30 mL) and washed with 1% NaOH, distilled water and brine. The organic layer was dried over Na_2SO_4, filtered and evaporated to afford the crude product which, in most cases, was used without further purification for the next step.
For selected compounds ^{1}H NMR spectral analysis was performed and compared with available literature data

Ethyl 2-(2-isopropylphenoxy)acetate (2a)

CAS Number: 444613-15-4. Yield 49% (colourless oil). $C_{13}H_{18}O_3$ (MW 222.28). ^{1}H NMR (500 MHz, $CDCl_3$) δ: 7.24 (d, *J* = 9.38 Hz, 1H, Ph-3-*H*), 7.13 (t, *J* = 7.03 Hz, 1H, Ph-4-*H*), 6.96 (t, *J* = 7.04, 1H, Ph-5-*H*), 6.72 (d, *J* = 8.20 Hz, 1H, Ph-6-*H*), 4.64 (s, 2H, OC*H*$_2$), 4.26 (q, *J* = 7.03 Hz, 2H, OC*H*$_2$CH$_3$), 3.42 (spt, *J* = 7.03 Hz, 1H, C*H*(CH$_3$)$_2$), 1.30 (t, *J* = 7.03 Hz, 3H, OCH$_2$C*H*$_3$). 1.32 - 1.24 (m, 9H, CH(C*H*$_3$)$_2$, OCH$_2$C*H*$_3$)
Synthesis and spectral data are available also in lit [3].

Ethyl 2-(2-tert-butylphenoxy)acetate (3a)

CAS Number: 93893-53-9. Yield 75% (yellow oil). $C_{14}H_{20}O_3$ (MW 236.31).^{1}H NMR (300 MHz, $CDCl_3$) δ: 7.33 (dd, *J* = 1.17, 7.62 Hz, 1H, Ph-3-*H*), 7.11 - 7.22 (m, 1H, Ph-5-*H*), 6.95 (def t, *J* = 6.44,

7.62 Hz, 1H, Ph-4-*H*), 6.74 (d, *J* = 8.21 Hz, 1H, Ph-6-*H*), 4.65 (s, 2H, -OCH_2), 4.29 (q, *J* = 7.03 Hz, 2H, OC$H_2$$CH_3$), 1.39 - 1.48 (m, 9H, 3x CH_3), 1.31 (t, *J* = 7.03 Hz, 3H, OCH_2CH_3)

Methyl 2-(3-isopropylphenoxy)acetate **(4a)**
CAS Number: 1040311-77-0. Yield 65% (colourless oil). $C_{13}H_{18}O_3$ (MW 222.28). ^{1}H NMR (500 MHz, $CDCl_3$) δ: 7.20 (t, *J* = 7.88 Hz, 1H, Ph-5-*H*), 6.87 (d, *J* = 7.73 Hz, 1H, Ph-4-*H*), 6.82 (s, 1H, Ph-2-*H*), 6.68 (dd, *J* = 8.31, 2.29 Hz, 1H, Ph-6-*H*), 4.64 (s, 2H, OCH_2), 4.26 (q, *J* = 7.03 Hz, 2H, OC$H_2$$CH_3$), 3.42 (spt, *J* = 7.03 Hz, 1H, C*H*$(CH_3)_2$), 1.30 (t, *J* = 7.03 Hz, 3H, OCH_2CH_3). 1.32 - 1.24 (m, 9H, CH(CH_3)$_2$, OCH_2CH_3)

Ethyl 2-(3-tert-butylphenoxy)acetate **(5a)**
CAS Number: 1094561-07-5. $C_{14}H_{20}O_3$ (MW 236.31) (crude product, yellow oil)

Ethyl 2-(4-isopropylphenoxy)acetate **(6a)**
CAS Number: 184878-99-7. Yield 63% (Colorless oil). $C_{13}H_{18}O_3$ (MW 222.28). ^{1}H NMR (300 MHz, $CDCl_3$) δ: 7.15 (d, *J* = 8.79 Hz, 2H, Ph-3,5-*H*), 6.85 (d, *J* = 8.21 Hz, 2H, Ph-2,6-*H*), 4.59 (s, 2H, OCH_2), 4.25 (q, *J* = 7.03 Hz, 2H, OC$H_2$$CH_3$), 2.85 (spt, *J* = 7.04 Hz, 1H, C*H*$(CH_3)_2$), 1.30 (t, *J* = 7.03 Hz, 3H, OCH_2CH_3). 1.22 (d, *J*= 7.04 Hz, 6H, CH(CH_3)$_2$)
Synthesis and spectral data are available also in lit [4].

Ethyl 2-(4-tert-butylphenoxy)acetate **(7a)**
CAS Number: 3344-19-2. $C_{14}H_{20}O_3$ (MW 236.31). (crude product; oily white solid)
Synthesis and spectral data are available in lit [5].

Methyl 2-(2-tert-butyl-5-methylphenoxy)acetate **(8a)**
CAS Number 1011532-01-6. $C_{15}H_{22}O_3$ (MW 250.33) (crude product, yellow oil)

Ethyl 2-(2-tert-butyl-4-methylphenoxy)acetate **(9a)**
CAS Number: 831199-72-5. Yield 50% (oily solid). $C_{15}H_{22}O_3$ (MW 250.33). ^{1}H NMR (300 MHz, DMSO-d_6) δ: 7.01 (s, 1H, Ph-3-*H*), 6.92 (d, *J* = 9.38 Hz, 1H, Ph-5-*H*), 6.72 (d, *J* = 8.21 Hz, 1H, Ph-6-*H*), 4.71 (s, 2H, -OCH_2), 4.14 (q, *J* = 1.00 Hz, 2H, OCH_2), 2.16 - 2.25 (m, 3H, CH_3), 1.25 - 1.44 (m, 9H, 3 x CH_3), 1.20 (t, *J* = 7.03 Hz, 3H, CH_2CH_3)

Ethyl 2-(2-tert-butyl-6-methylphenoxy)acetate **(10a)**
CAS Number: none. $C_{15}H_{22}O_3$ (MW 250.34) (crude product, colorless oil)

Ethyl 2-(2,6-diisopropylphenoxy)acetate **(11a)**
CAS Number: 145546-92-5. Yield 73% (colorless oil). $C_{16}H_{24}O_3$ (MW 264.36). ^{1}H NMR (500 MHz, $CDCl_3$) δ: 7.09 - 7.12 (m, 3 H, Ph-*3,4,5-H*), 4.38 (s, 2 H, OCH_2), 4.31 (q, *J*=6.87 Hz, 2 H, OC$H_2$$CH_3$), 3.33 (spt, *J*=6.90 Hz, 2 H, 2x C*H*$(CH_3)_2$), 1.34 (t, *J*=7.16 Hz, 3 H, OCH_2CH_3), 1.22 (d, *J*=6.87 Hz, 12 H, 2x CH(CH_3)$_2$)

Ethyl 2-(2,6-di-tert-butylphenoxy)acetate **(12a)**
CAS Number: 27955-32-4. $C_{18}H_{18}O_3$ (MW=292.42) (crude product, colorless oil)

Ethyl 2-(2,4-di-tert-butylphenoxy)acetate **(13a)**
CAS Number: none $C_{18}H_{18}O_3$ (MW 292.42) (crude product, colorless oil)

Ethyl 4-(2-isopropyl-5-methylphenoxy)butanoate **(14a)**
CAS Number: none $C_{16}H_{24}O_3$ (MW 264.37) (crude product, pale yellow oil)

Ethyl 4-(2-isopropylphenoxy)butanoate **(15a)**
CAS Number: none. $C_{15}H_{22}O_3$ (MW 250.34) (crude product, pale yellow oil)

***Ethyl 4-(4-isopropylphenoxy)butanoate* (16a)**
CAS Number: none. $C_{15}H_{22}O_3$ (MW 250.34) (crude product, pale yellow oil)

***Ethyl 4-(m-tolyloxy)butanoate* (17a)**
CAS Nr 56359-12-7. $C_{13}H_{18}O_3$ (MW 222.28) (crude product, colorless oil). ^{1}H NMR (500 MHz, $CDCl_3$) d: 7.14 (t, *J*=7.88 Hz, 1H, Ph-*5-H*), 6.74 (d, *J*=7.45 Hz, 1H, Ph-*4-H*), 6.65-6.71 (m, 2H, Ph-*2,6-H*), 4.13 (q, *J*=7.88 Hz, 2H, OCH_2CH_3), 3.98 (t, *J*=6.01 Hz, 2H, OCH_2), 2.50 (t, *J*=7.30 Hz, 2H, CH_2CO), 2.31 (s, 3H, Ph-CH_3), 2.09 (quin, *J*=6.73 Hz, 2H, $CH_2CH_2CH_2$), 1.24 (t, 3H, *J*=7.44 Hz, OCH_2CH_3)
Synthetic and spectral data are available also in lit [6].

***Ethyl 4-(o-tolyloxy)butanoate* (18a)**
CAS Number: 56359-11-6. $C_{13}H_{18}O_3$ (MW 222.28) (crude product, colorless oil)
Synthesis and spectral data are available in lit [7].

***Ethyl 5-(2-isopropyl-5-methylphenoxy)pentanoate* (19a)**
CAS Number: none. $C_{17}H_{26}O_3$ (MW 278.39) (crude product, pale yellow oil)

***Ethyl 5-(2-isopropylphenoxy)pentanoate* (20a)**
CAS Number: none. $C_{16}H_{24}O_3$ (MW 264.37) (crude product, pale yellow oil)

***Ethyl 5-(m-tolyloxy)pentanoate* (21a)**
CAS Number: none. $C_{14}H_{20}O_3$ (MW 236.31) (crude product, colorless oil). ^{1}H NMR (500 MHz, $CDCl_3$) d: 7.14 (t, *J*=7.59 Hz, 1H, Ph-*5-H*), 6.74 (d, *J*=6.87 Hz, 1H, Ph-*4-H*), 6.65-6.72 (m, 2H, Ph-*2,6-H*), 4.08-4.16 (m, 2H, OCH_2CH_3), 3.94 (br.s., 2H, OCH_2), 2.36 (br.s., 2H, CH_2CO), 2.31 (s, 3H, Ph-CH_3), 1.80 (def q, 4H, $CH_2CH_2CH_2CH_2$), 1.20 - 1.29 (t, 3H, *J*=7.16 Hz, OCH_2CH_3)

***Ethyl 6-(2-isopropyl-5-methylphenoxy)hexanoate* (22a)**
CAS 1410584-62-1. $C_{18}H_{28}O_3$ (MW 292.42) (crude product, pale yellow oil)

***Ethyl 6-(2-isopropylphenoxy)hexanoate* (23a)**
CAS 1468436-17-0. $C_{17}H_{26}O_3$ (MW 278.39) (crude product, colorless oil). ^{1}H NMR (500 MHz, $CDCl_3$) d: 7.19 (dd, *J*=7.45, 1.43 Hz, 1H, Ph-6-*H*), 7.10 - 7.15 (m, 1H, Ph-6-H), 6.90 (t, *J*=7.45 Hz, 1H, Ph-3-H), 6.80 (d, *J*=8.02 Hz, 1H, Ph-2-H), 4.12 (q, *J*=7.16 Hz, 2H, OCH_2CH_3), 3.95 (t, *J*=6.30 Hz, 2H, CH_2CO), 3.26 - 3.35 (m, 1H, $CH(CH_3)_2$), 2.33 (t, J = 7.4 Hz, 2H, CH_2CO), 1.78 - 1.86 (m, 2H, OCH_2CH_2), 1.71 (quin, *J*=7.59 Hz, 2H, $CH_2CH_2CH_2$), 1.48 - 1.57 (m, 2H, CH_2CH_2CO), 1.22 - 1.27 (t, 3H, OCH_2CH_3), 1.20 (d, *J*=6.87 Hz, 6H, $CH(CH_3)_2$)

***Ethyl 6-(m-tolyloxy)hexanoate* (24a)**
CAS Number (14695119-65-7). $C_{15}H_{22}O_3$ (MW 250.34) (crude product, colorless oil). ^{1}H NMR (500 MHz, $CDCl_3$) d: 7.13 (t, *J*=7.88 Hz, 1H, Ph-*5-H*), 6.73 (d, *J*=7.45 Hz, 1H, Ph-*4-H*), 6.65-6.71 (m, 2H, Ph-*2,6-H*), 4.11 (q, *J*=6.97 Hz, 2H, OCH_2CH_3), 3.92 (t, *J*=6.44 Hz, 2H, OCH_2), 2.33 (m, 5H, Ph-CH_3+CH_2CO), 1.77 (quin, J=7.73 Hz, 2H, OCH_2CH_2), 1.68 (quin, *J*=7.59 Hz, 2H, CH_2CH_2CO), 1.47 - 1.51 (m, 2H, $CH_2CH_2CH_2$,), 1.24 (t, 3H, *J*=7.45 Hz, OCH_2CH_3)

***Ethyl 7-(2-isopropyl-5-methylphenoxy)heptanoate* (25a)**
CAS Number: none. $C_{19}H_{30}O_3$ (MW 306.45) (crude product, colorless oil). ^{1}H NMR (500 MHz, $CDCl_3$) d: 7.07 (d, *J*=7.73 Hz, 1H, Ph-3-*H*), 6.71 (d, *J*=7.73 Hz, 1H, Ph-*4-H*), 6.63 (s, 1H, Ph-*6-H*), 4.12 (q, *J*=7.16 Hz, 2H, OCH_2CH_3), 3.93 (t, *J*=6.30 Hz, 2H, OCH_2), 3.27 (spt, J=6.95 Hz, 1H, $CH(CH_3)_2$), 2.25-2.35 (m, 5H, Ph-CH_3+CH_2CO), 1.75-1.83 (m, 2H, OCH_2CH_2), 1.61-1.70 (m, 2H, CH_2CH_2CO), 1.46-1.55 (m, 2H, $OCH_2CH_2CH_2$), 1.37-1.44 (m, 2H, $CH_2CH_2CH_2CO$), 1.22 - 1.27 (t, J=6.88 Hz, 3H, OCH_2CH_3), 1.19 (d, *J*=7.16 Hz, 6H, $CH(CH_3)_2$)

References

1. Łażewska D.; Więcek M. Ner J.; Kamińska K., Kottke T.,; Schwed J.S.; Zygmunt M.; Karcz T.;Olejarz A.; Kuder K.; Latacz G.; Grosicki M.; Sapa J.; Karolak-Wojciechowska J.; Stark

H.; Kieć-Kononowicz K. Aryl-1,3,5-triazine derivatives as histamine H4 receptor ligands. *Eur J Med Chem* **2014**, 83, 534-46. Doi:10.1016/j.ejmech.2014.06.032

2. Ali, W.; Więcek, M.; Łażewska, D.; Kurczab, R.; Jastrzębska-Więsek, M.; Satała, G.; Kucwaj-Brysz, K.; Lubelska, A.; Głuch-Lutwin, M.; Mordyl, B.; Siwek, A.; Nasim, M.J.; Partyka, A.; Sudoł, S.; Latacz, G.; Wesołowska, A.; Kieć-Kononowicz, K.; Handzlik, J. Synthesis and computer-aided SAR studies for derivatives of phenoxyalkyl-1,3,5-triazine as the new potent ligands for serotonin receptors 5-HT_6. *Eur J Med Chem* **2019**, *178*, 740-751. doi: 10.1016/j.ejmech.2019.06.022.
3. Mohammed Y.H.E.; Malojirao V.H.; Thirusangu P.; Al-Ghorbani M.; Prabhakar B.T.; Khanum S.A. The Novel 4-Phenyl-2-Phenoxyacetamide Thiazoles modulates the tumor hypoxia leading to the crackdown of neoangiogenesis and evoking the cell death. *Eur J Med Chem* **2018**, 143, 1826-39. Doi:10.1016/j.ejmech.2017.10.082.
4. Bagley S.W.; Brandt T.; Dugger R.W.; Hada W. A.; Hayward Ch.M.;Liu Z. *PCT Int. Appl., 2004048334*, 10 Jun 2004
5. Lee, Kyeong; Goo, Ja-Il; Jung, Hwa Young; Kim, Minkyoung; Boovanahalli, Shanthaveerappa K.; Park, Hye Ran; Kim, Mun-Ock; Kim, Dong-Hyun; Lee, Hyun Sun; Choi, Yongseok. Discovery of a novel series of benzimidazole derivatives as diacylglycerol acyltransferase inhibitors. *Bioorg Med Chem Lett* **2008**, *22(24)*, 7456-7460. doi: 10.1016/j.bmcl.2012.10.046
6. Manuel Amézquita-Valencia and Howard Alper. Regioselective Alkoxycarbonylation of Allyl Phenyl Ethers Catalyzed by Pd/dppb Under Syngas Conditions. *J Org Chem* **2016**, 81, 3860-3867. doi: 10.1021/acs.joc.6b00522
7. Mohammed Al-Ghorbani; V. Vigneshwaran; V. Lakshmi Ranganatha;, B.T. Prabhakar; Shaukath Ara Khanum. Synthesis of oxadiazole–morpholine derivatives and manifestation of the repressed CD31 Microvessel Density (MVD) as tumoral angiogenic parameters in Dalton’s Lymphoma. *Bioorganic Chemistry* **2015**, *60*, 136–146. doi.10.1016/j.bioorg.2015.04.008

2. ^{1}H NMR spectra of selected compounds

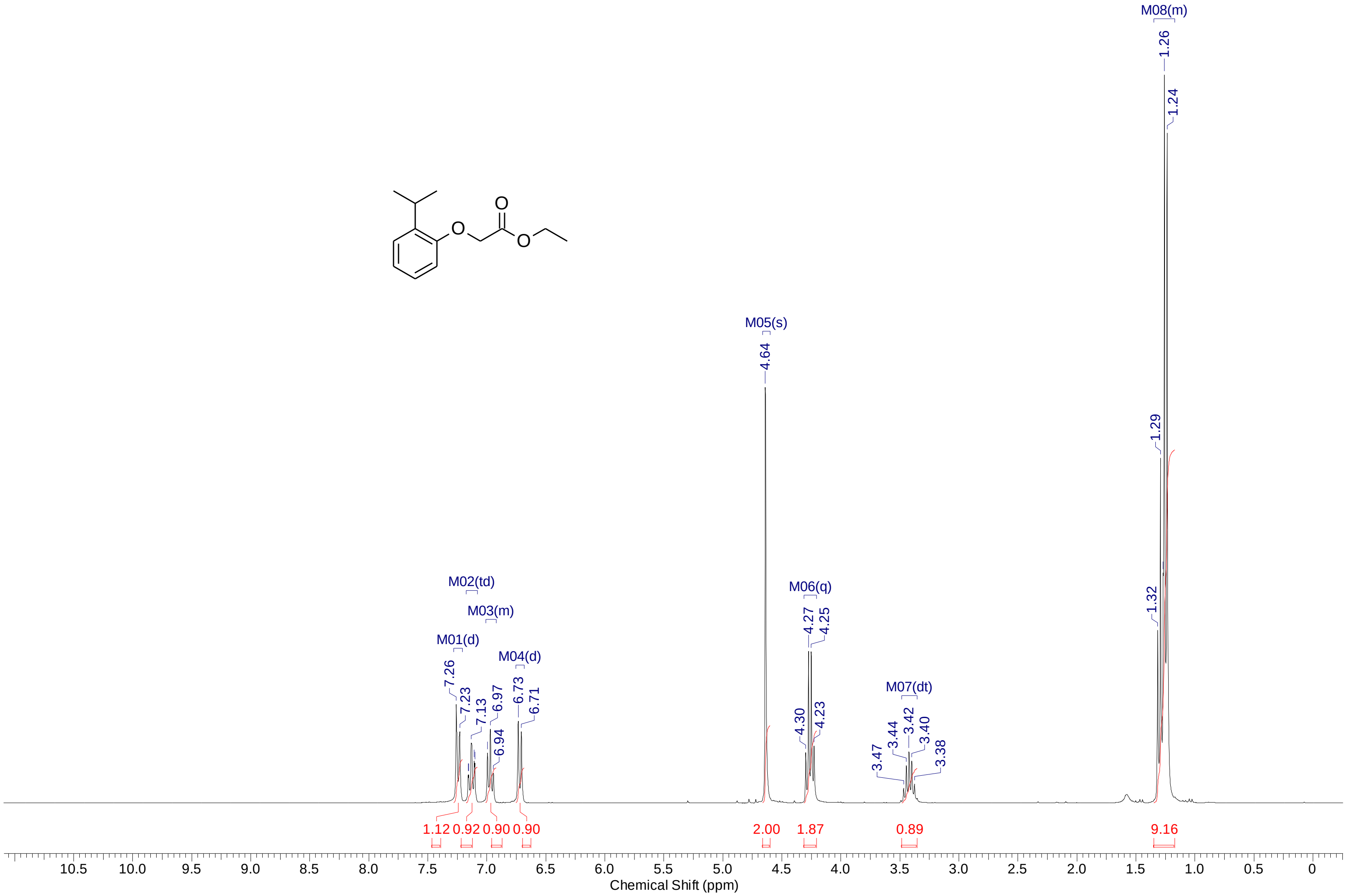

Fig. S1. ^{1}H NMR spectrum of **2a**

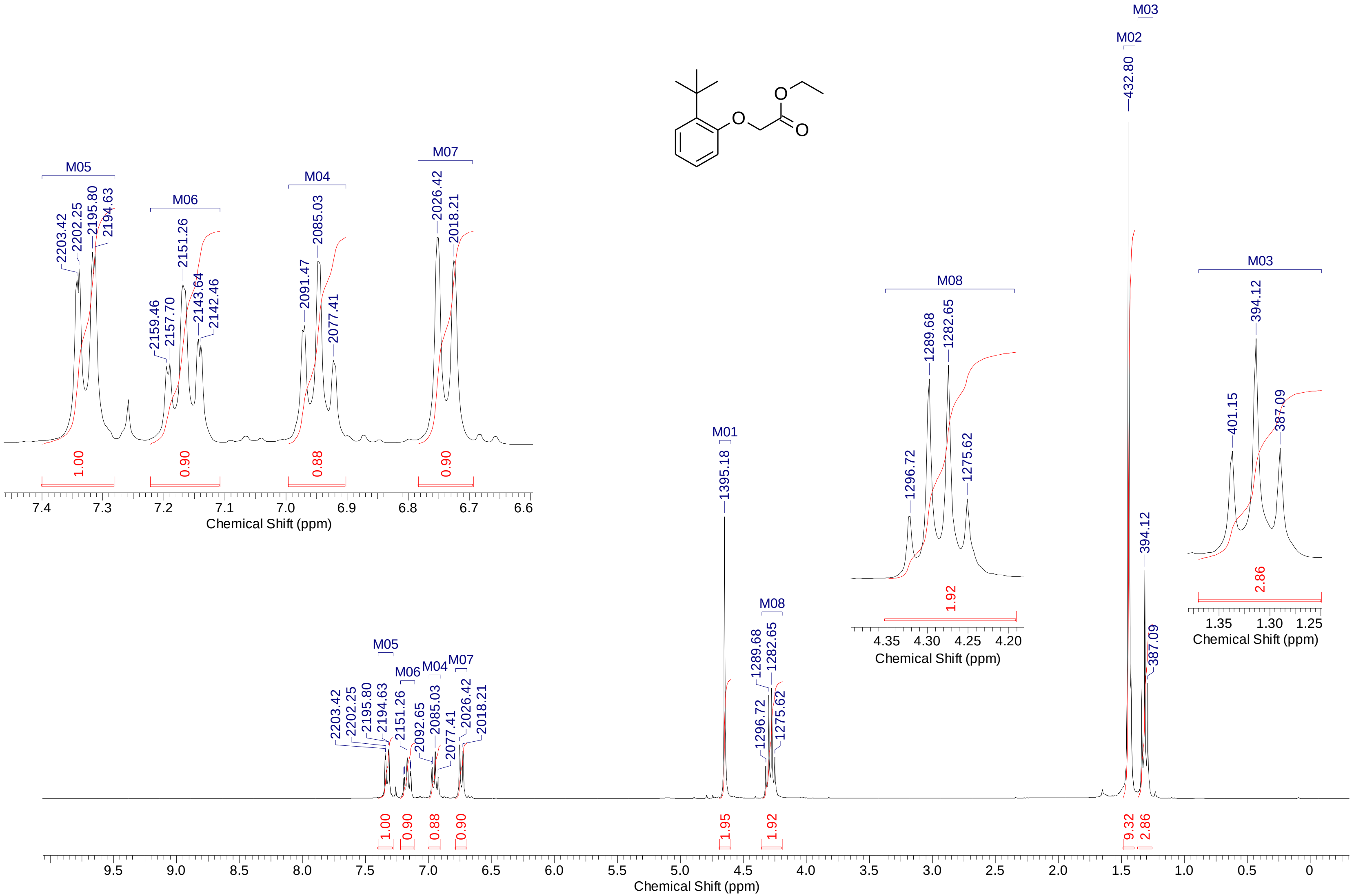

Fig. S2. ^{1}H NMR spectrum of **3a**

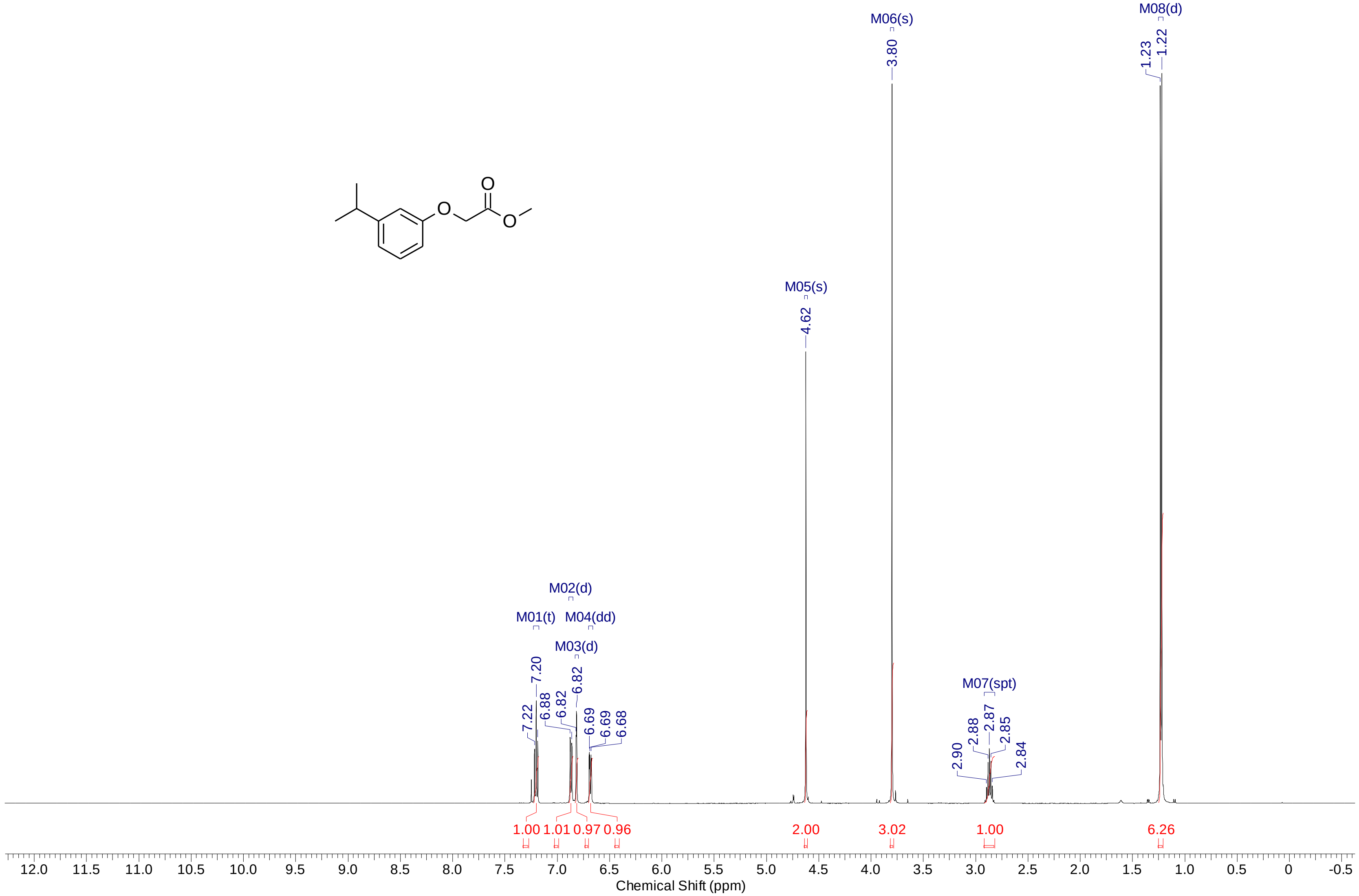

Fig. S3. ^{1}H NMR spectrum of **4a**

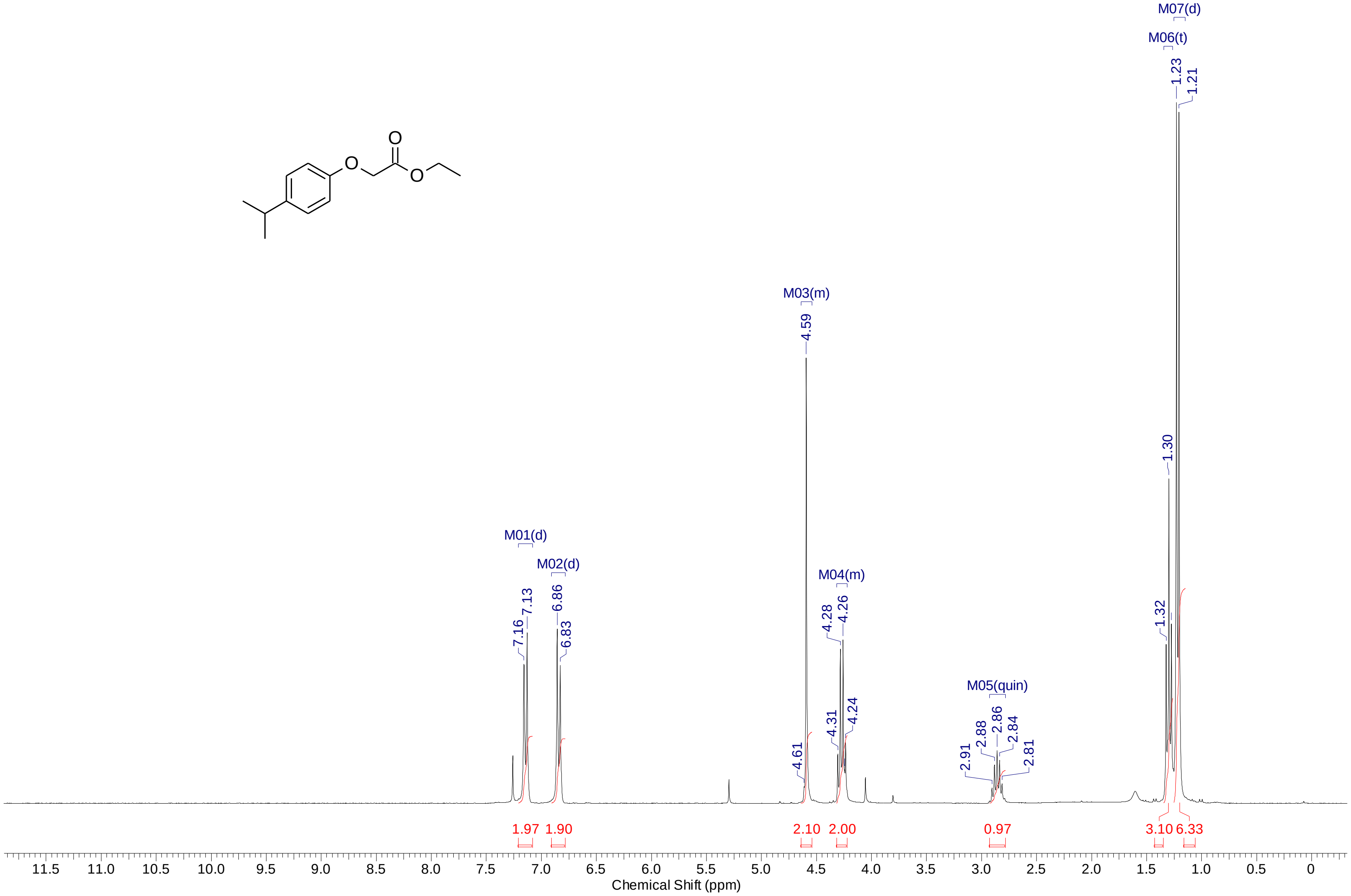

Fig. S4. ^{1}H NMR spectrum of **6a**

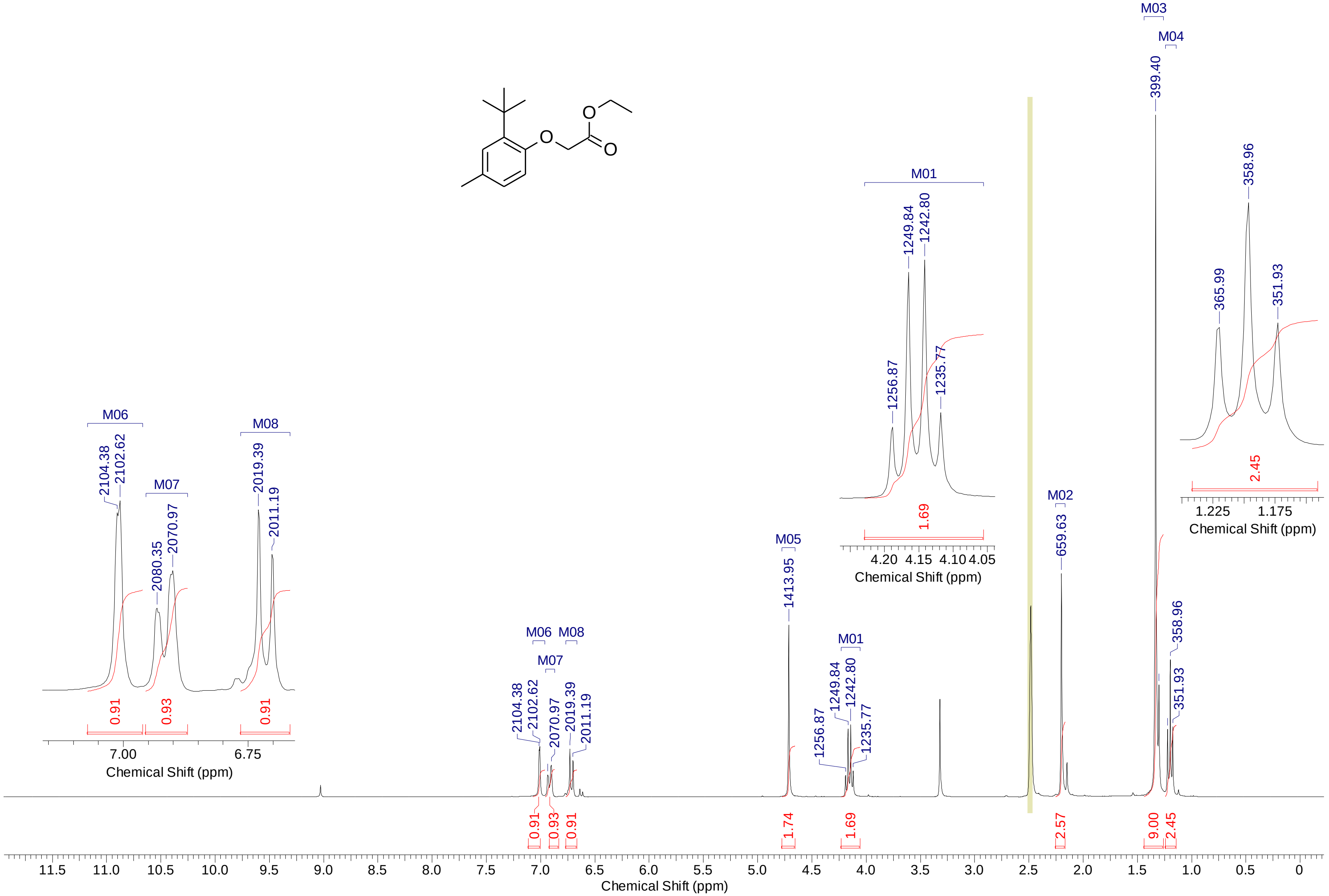

Fig. S5. ¹H NMR spectrum of **9a**

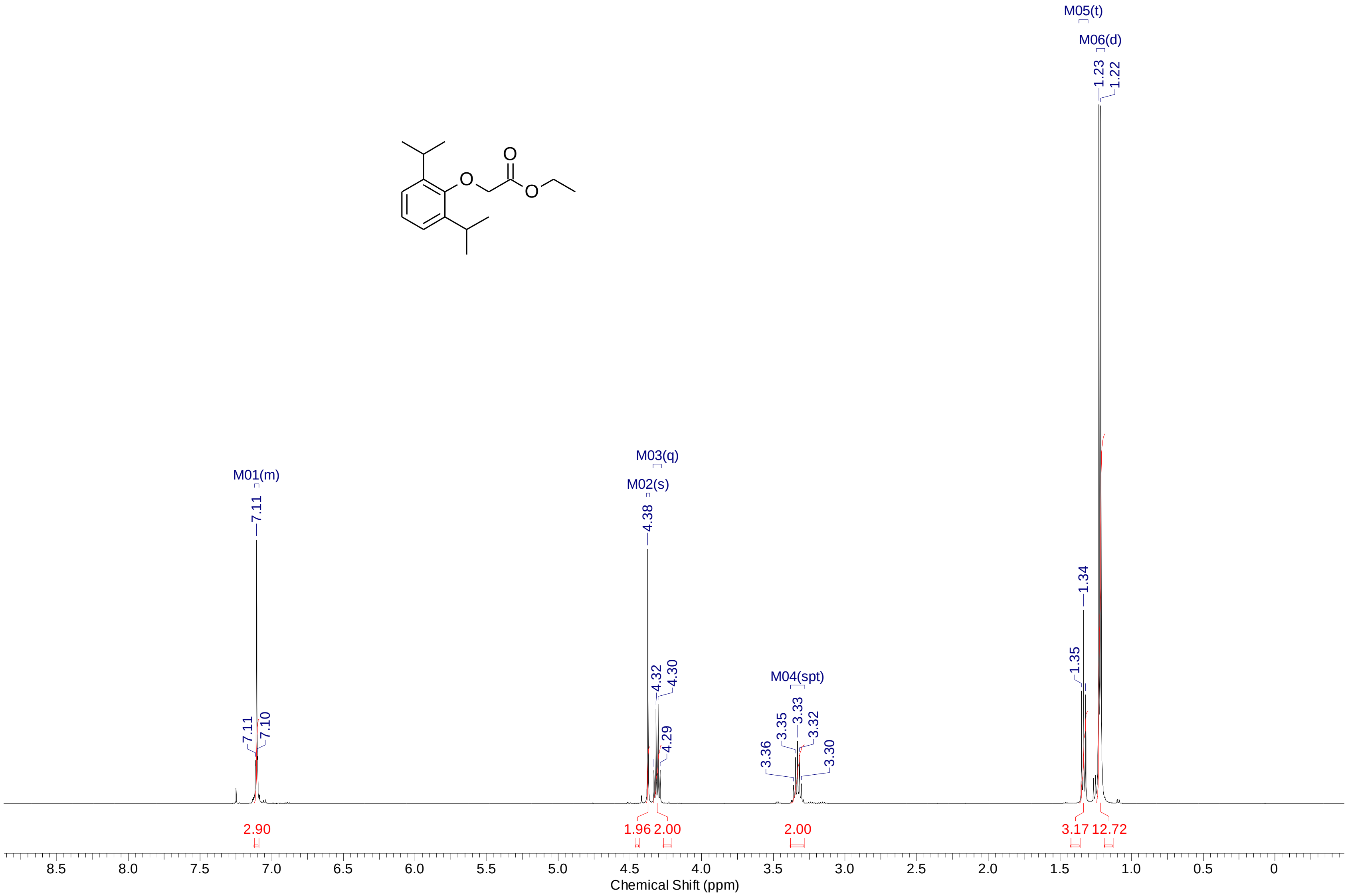

Fig. S6. ^{1}H NMR spectrum of **11a**

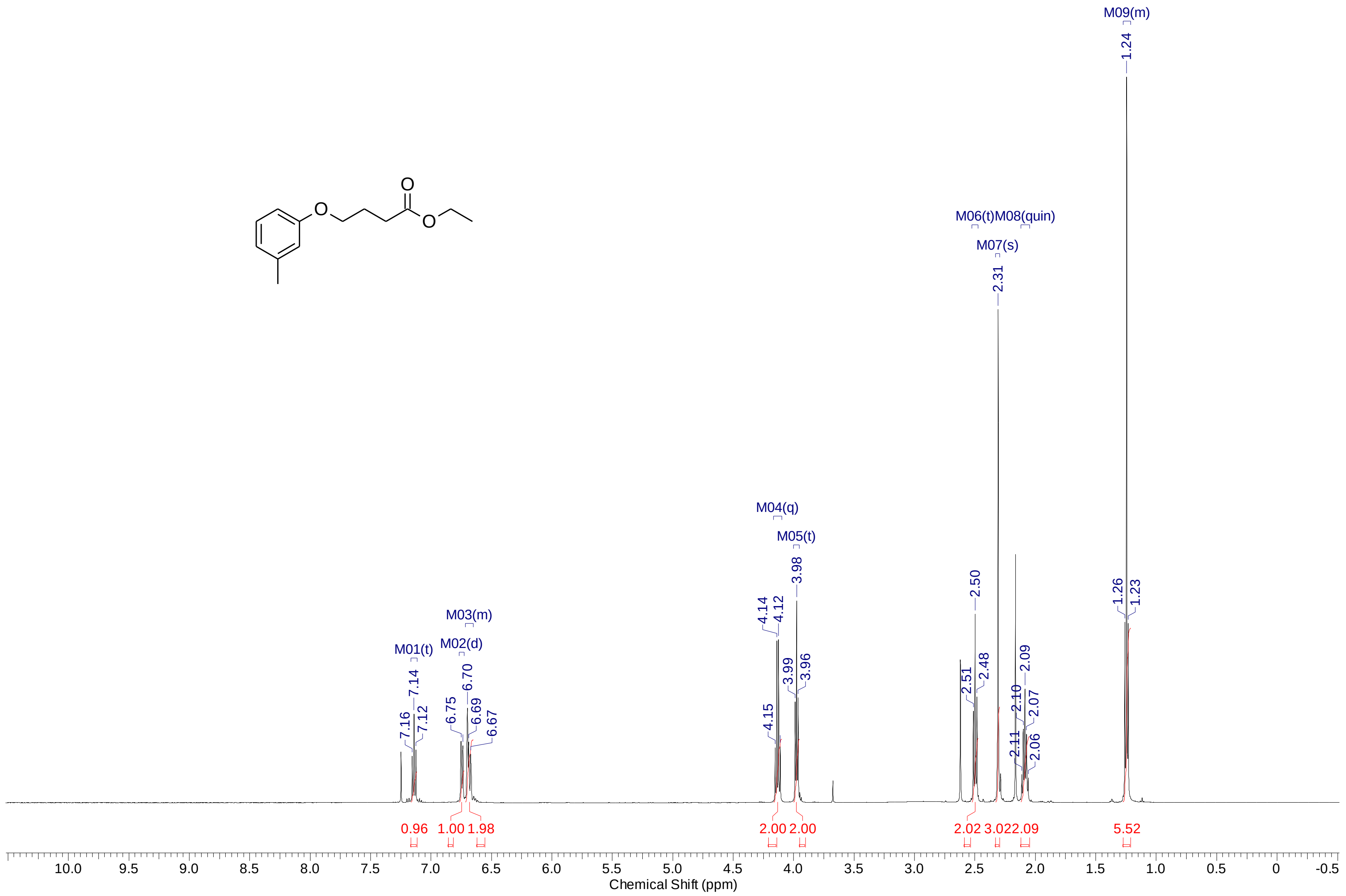

Fig. S7. ^{1}H NMR spectrum of **17a**

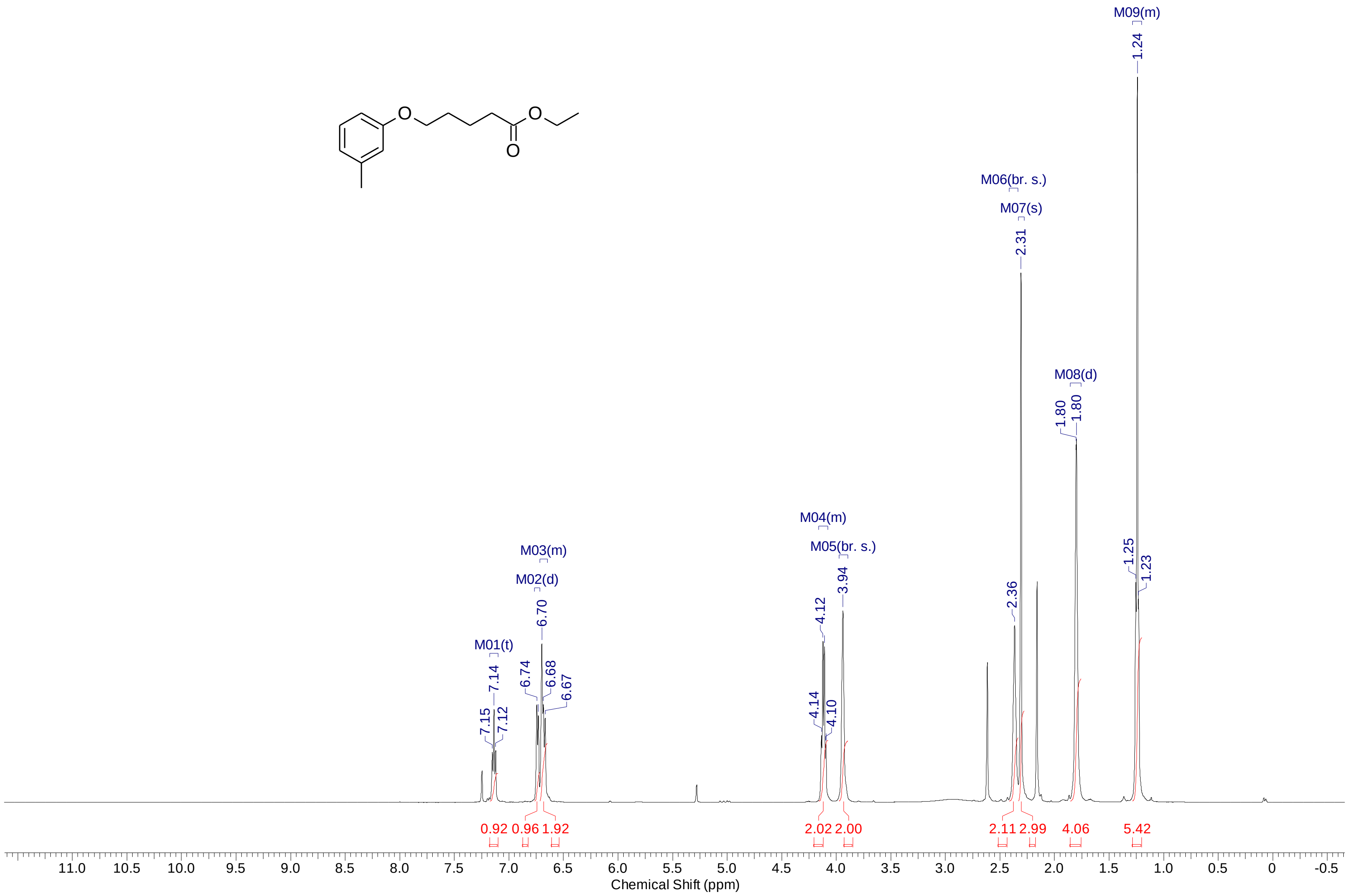

Fig. S8. ^{1}H NMR spectrum of **21a**

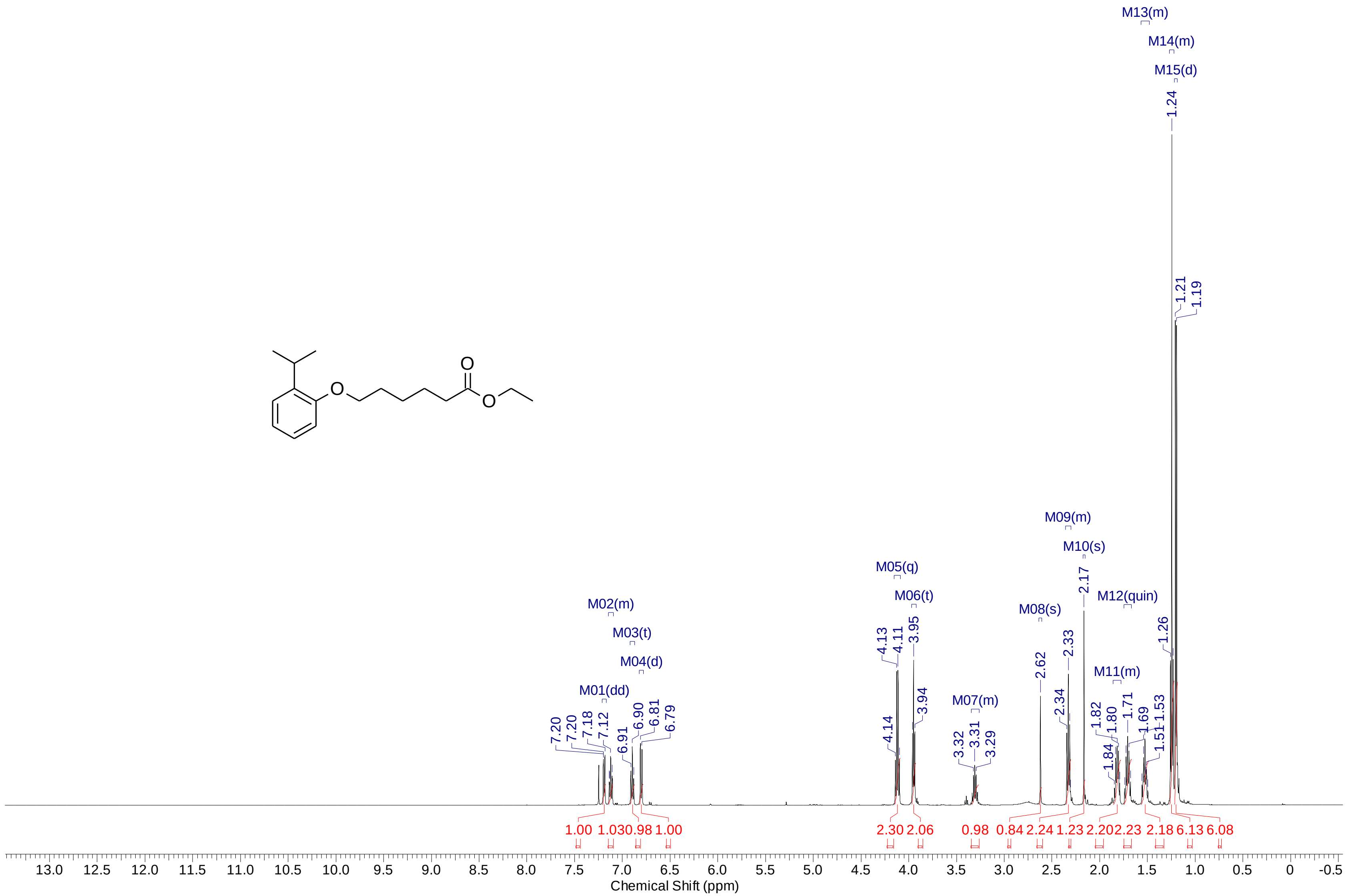

Fig. S9. ^{1}H NMR spectrum of **23a**

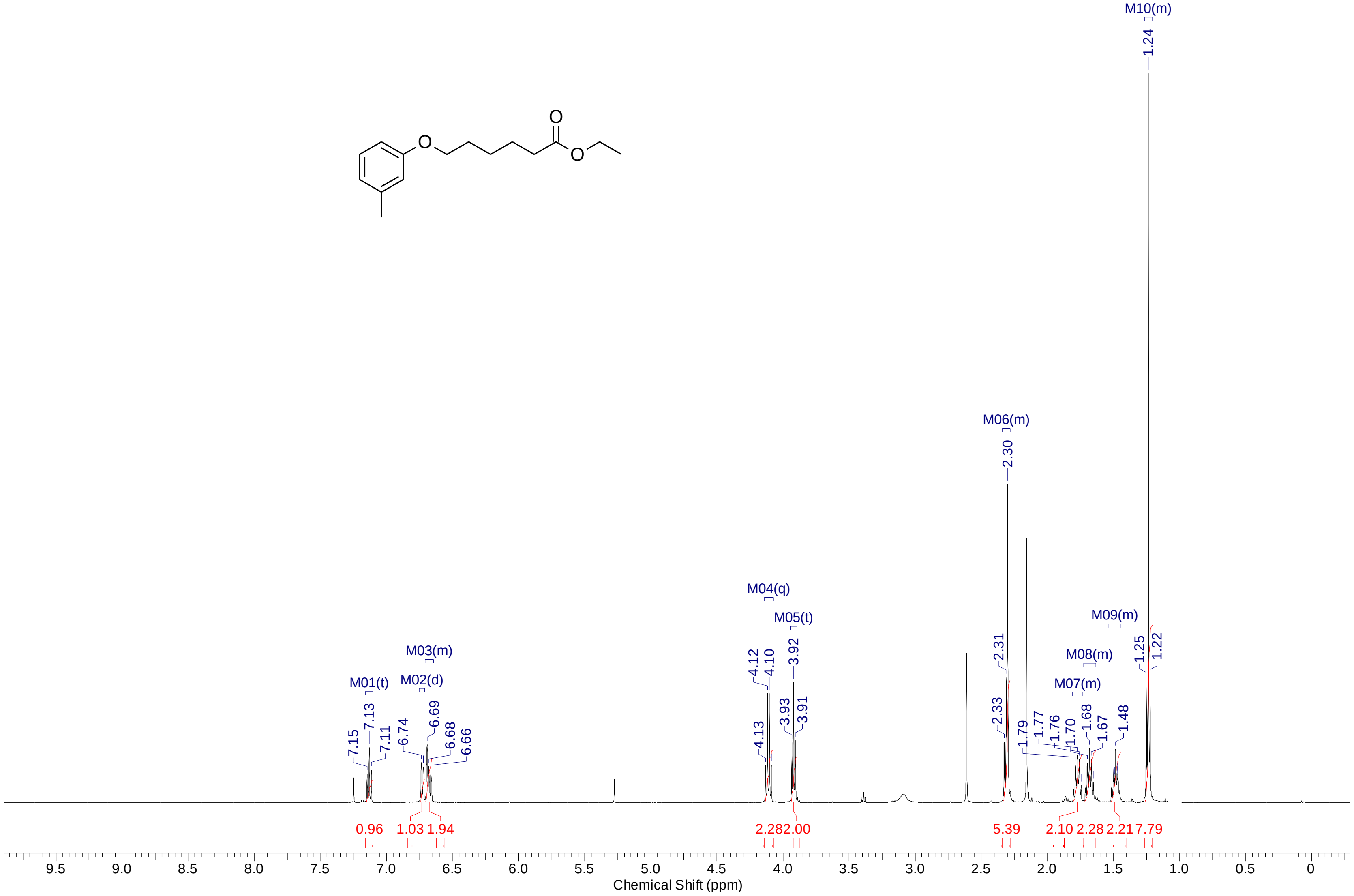

Fig. S10. ^{1}H NMR spectrum of **24a**

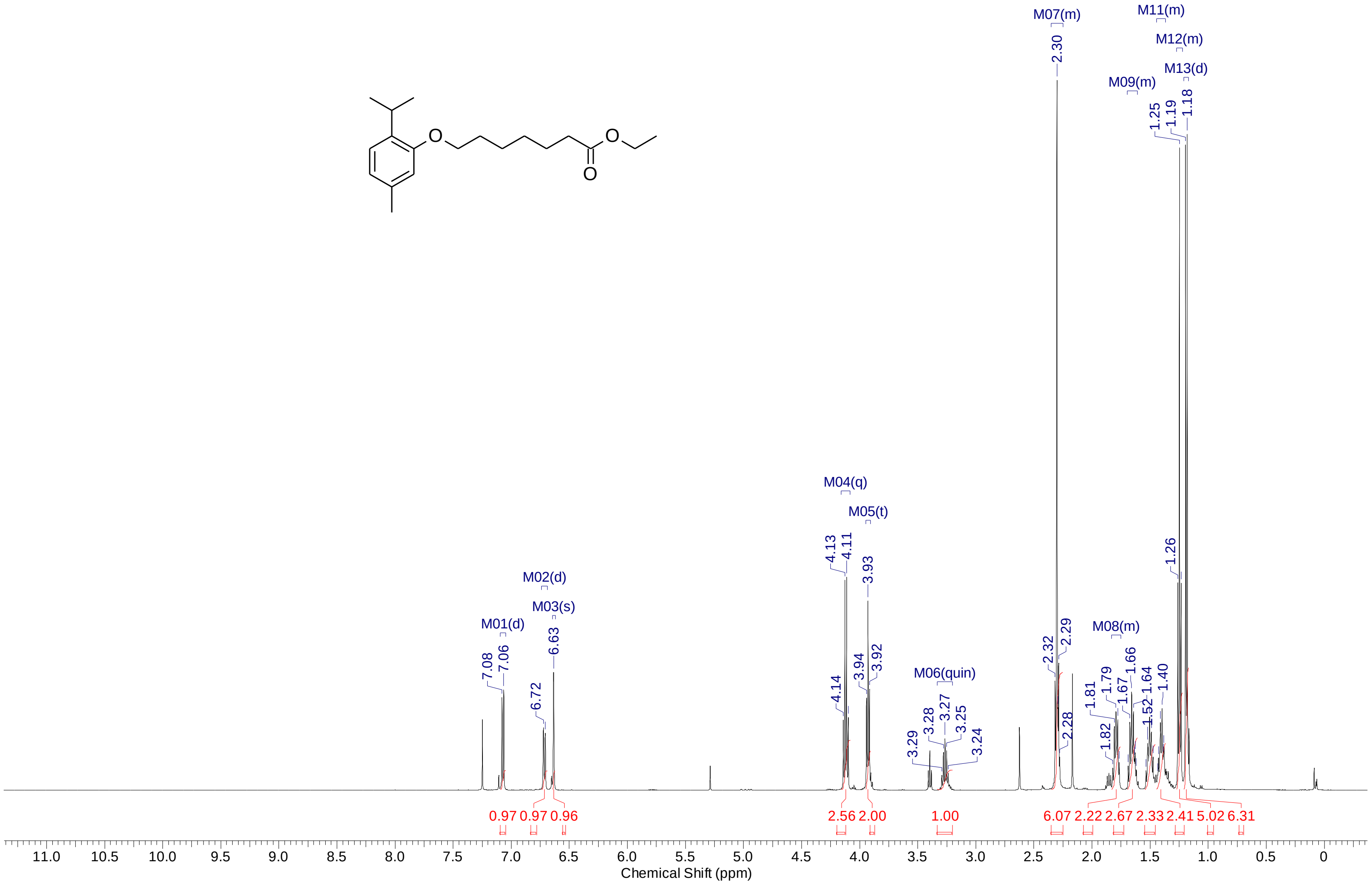

Fig. S11. ^{1}H NMR spectrum of **25a**

3. Water solubility determination

The water solubility of the selected compounds was determined using UV spectroscopy following previously described methods [8,9]. The calibration curves were determined using a series of dilutions for each compound. Stock solutions with concentrations of 1 mg/ml were obtained by dissolving 2.5 mg of each compound in methanol (90% v/v), followed by dilution to obtain solutions with concentrations in the range of 10^{-3}-10^{-1} mg/ml. For each sample the calibration curves were constructed based on absorbance measurements at 268 nm. Saturated solutions of tested compounds were prepared by suspending each compound (10 mg) in H_2O (2 mL). The suspensions were refluxed for 5 minutes, then left overnight at 20 ºC and filtered off using *Macherey-Nagel MN 619 de* filter. Individual filtrates were diluted in methanol (from 10 to 160 times), starting with the preparation of a stock solution containing 0.8 ml of filtrate and 7.2 ml of MeOH, and analyzed by UV spectroscopy as a solution in methanol/water (90% v/v). The concentrations of saturated solutions were calculated using MS Excel by linear regression of the two vicinal points of the calibration curves and multiplication by the dilution rate.

9. Załuski, M.; Stanuch, K.; Karcz, T.; Hinz, S.; Latacz, G.; Szymańska, E.; Schabikowski, J.; Doroz-Płonka, A.; Handzlik, J.; Drabczyńska, A.; Müller, C. E.; Kieć-Kononowicz, K. Tricyclic Xanthine Derivatives Containing a Basic Substituent: Adenosine Receptor Affinity and Drug-Related Properties. *MedChemComm* **2018**, *9(6)*, 951–962. https://doi.org/10.1039/c8md00070k.
10. Szymańska, E.; Drabczyńska, A.; Karcz, T.; Müller, C. E.; Köse, M.; Karolak-Wojciechowska, J.; Fruziński, A.; Schabikowski, J.; Doroz-Płonka, A.; Handzlik, J.; Kieć-Kononowicz, K. Similarities and Differences in Affinity and Binding Modes of Tricyclic Pyrimido- and Pyrazinoxanthines at Human and Rat Adenosine Receptors. *Bioorg Med Chem* **2016**, *24(18)*, 4347–4362. https://doi.org/10.1016/j.bmc.2016.07.028.

Calibration curves:

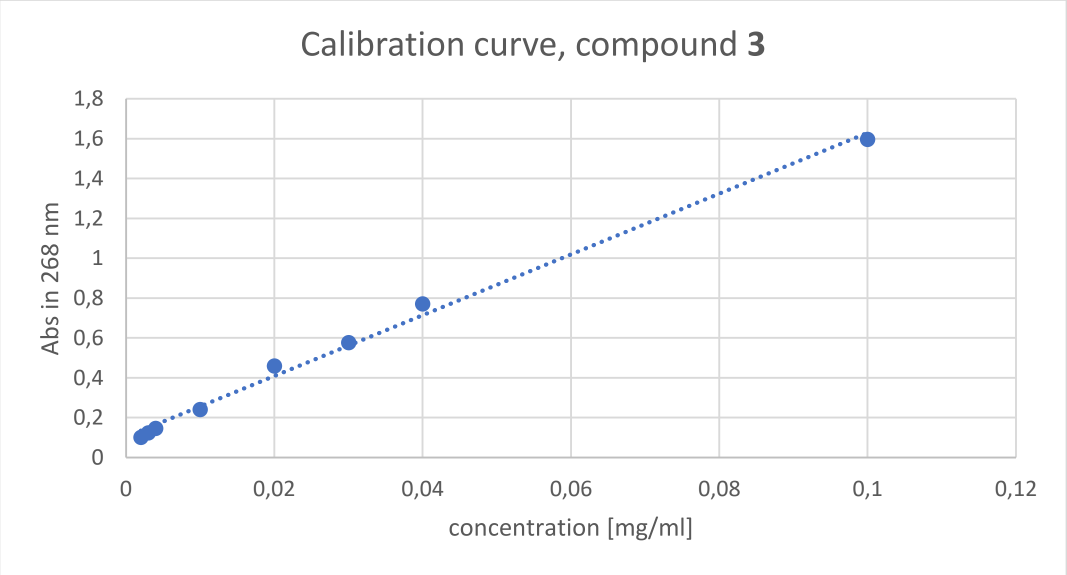

Fig. S12. Calibration curve for compound **3**.

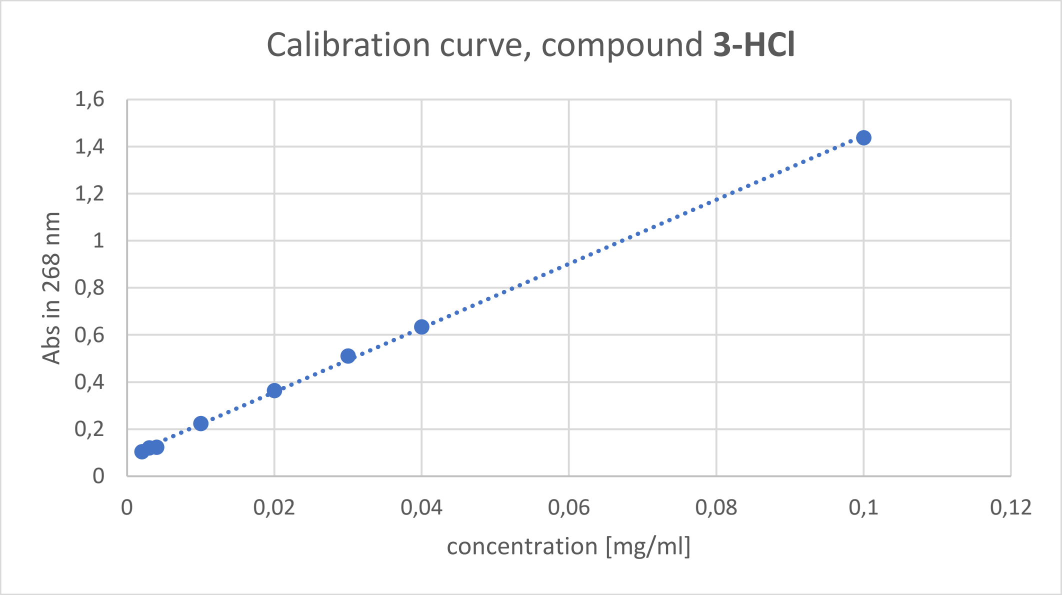

Fig. S13. Calibration curve for compound **3-HCl**.

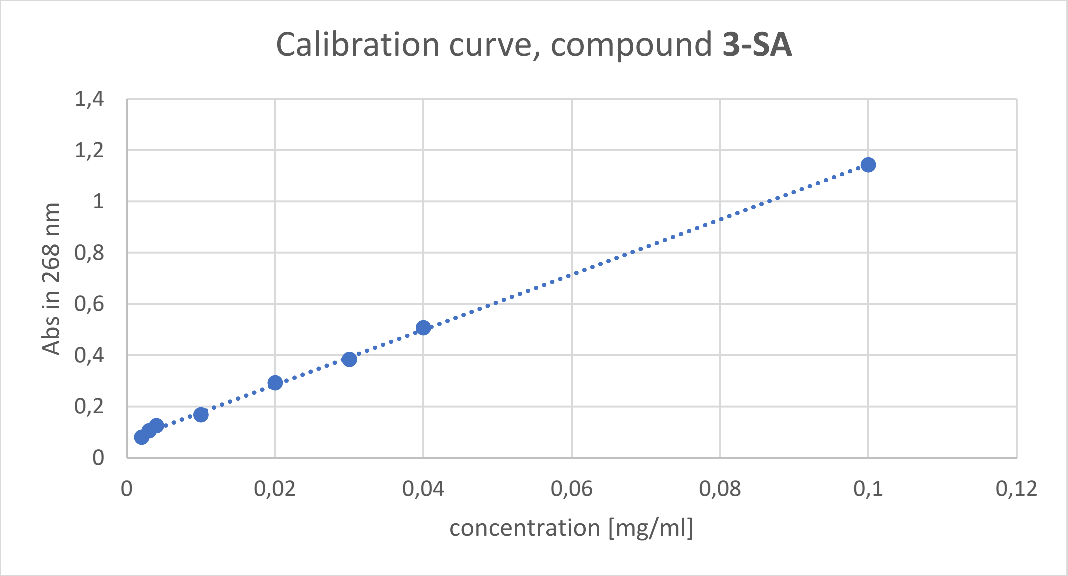

Fig. S14. Calibration curve for compound **3-SA**.

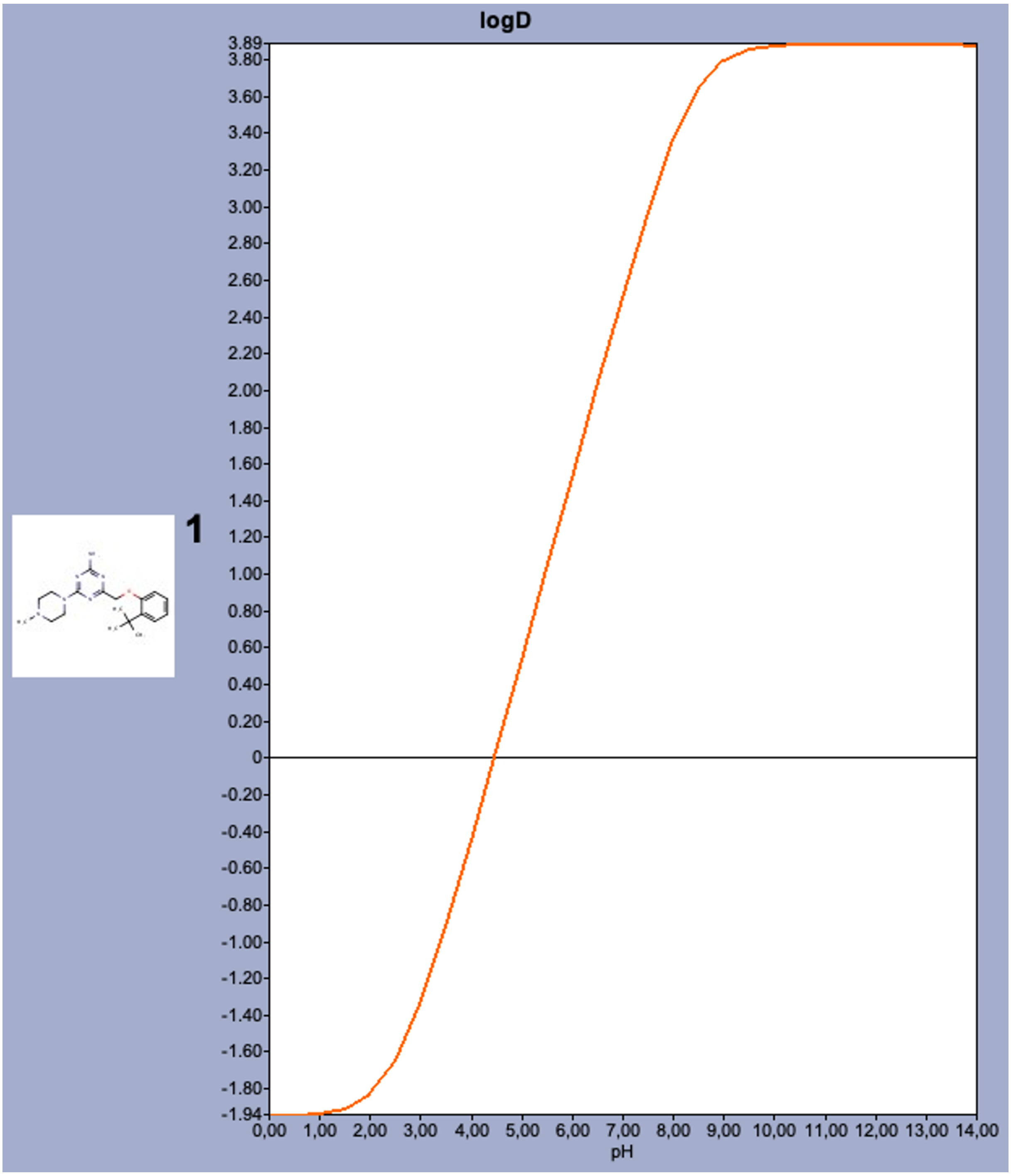

Fig. S15. LogD prediction by Marvin (ChemAxon) for compound **3**.

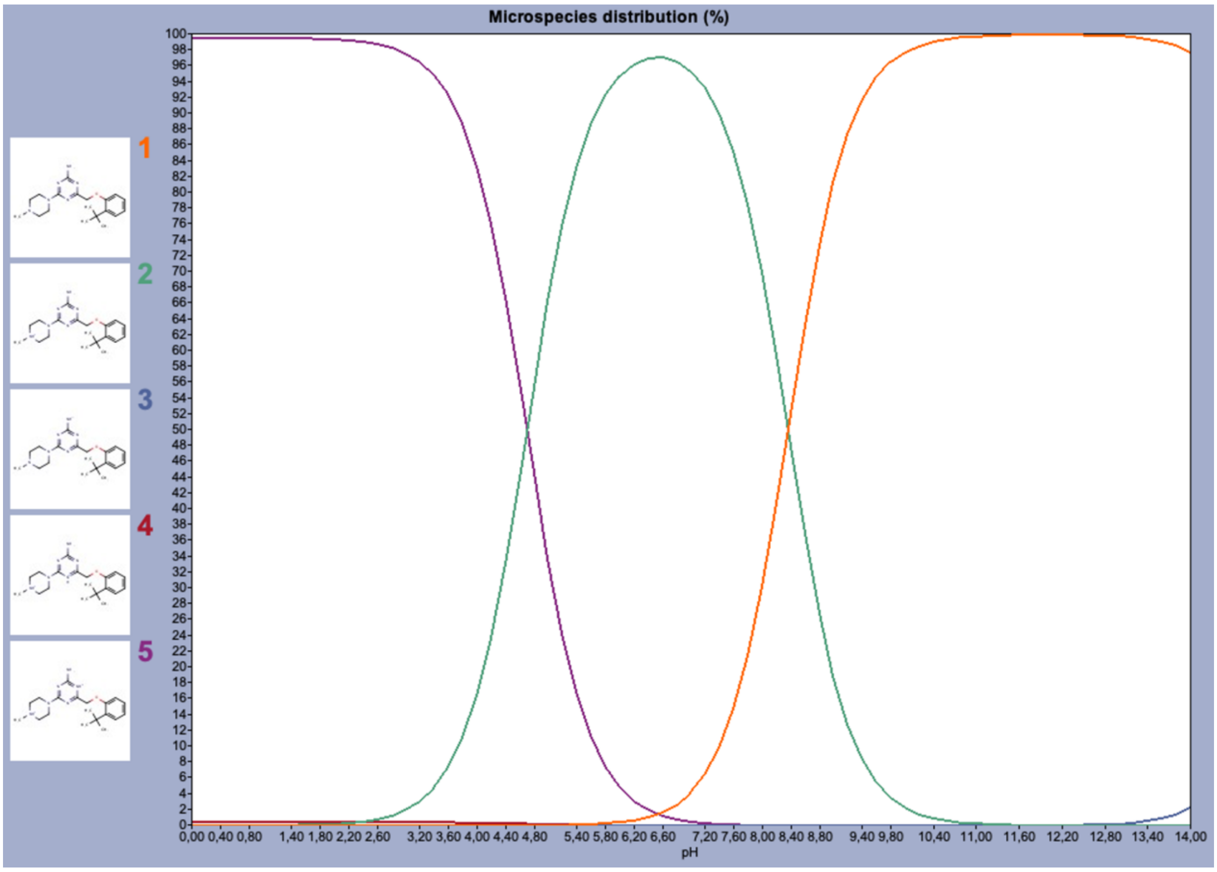

Fig. S16. pK_a prediction by Marvin (ChemAxon) for compound **3**.
